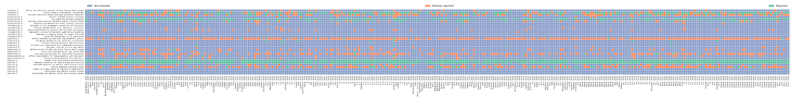# AI in radiological imaging of soft-tissue and bone tumours: a systematic review evaluating against CLAIM and FUTURE-AI guidelines

**DOI:** 10.1016/j.ebiom.2025.105642

**Published:** 2025-03-20

**Authors:** Douwe J. Spaanderman, Matthew Marzetti, Xinyi Wan, Andrew F. Scarsbrook, Philip Robinson, Edwin H.G. Oei, Jacob J. Visser, Robert Hemke, Kirsten van Langevelde, David F. Hanff, Geert J.L.H. van Leenders, Cornelis Verhoef, Dirk J. Grünhagen, Wiro J. Niessen, Stefan Klein, Martijn P.A. Starmans

**Affiliations:** a Department of Radiology and Nuclear Medicine, Erasmus MC Cancer Institute, University Medical Center Rotterdam, Rotterdam, the Netherlands; b Department of Medical Physics, Leeds Teaching Hospitals NHS Trust, UK; c Leeds Biomedical Research Centre, University of Leeds, UK; d Department of Radiology, Leeds Teaching Hospitals NHS Trust, UK; e Leeds Institute of Medical Research, University of Leeds, UK; f Department of Radiology and Nuclear Medicine, Amsterdam UMC, Amsterdam, the Netherlands; g Department of Radiology, Leiden University Medical Center, Leiden, the Netherlands; h Department of Pathology, Erasmus MC Cancer Institute, University Medical Center Rotterdam, Rotterdam, the Netherlands; i Department of Surgical Oncology, Erasmus MC Cancer Institute, University Medical Center Rotterdam, Rotterdam, the Netherlands; j Faculty of Medical Sciences, University of Groningen, Groningen, the Netherlands

**Keywords:** Systematic review, Soft-tissue and bone tumours, Radiological imaging, Artificial intelligence, Medical image analysis, FUTURE-AI, CLAIM

## Abstract

**Background:**

Soft-tissue and bone tumours (STBT) are rare, diagnostically challenging lesions with variable clinical behaviours and treatment approaches. This systematic review aims to provide an overview of Artificial Intelligence (AI) methods using radiological imaging for diagnosis and prognosis of these tumours, highlighting challenges in clinical translation, and evaluating study alignment with the Checklist for AI in Medical Imaging (CLAIM) and the FUTURE-AI international consensus guidelines for trustworthy and deployable AI to promote the clinical translation of AI methods.

**Methods:**

The systematic review identified literature from several bibliographic databases, covering papers published before 17/07/2024. Original research published in peer-reviewed journals, focused on radiology-based AI for diagnosis or prognosis of primary STBT was included. Exclusion criteria were animal, cadaveric, or laboratory studies, and non-English papers. Abstracts were screened by two of three independent reviewers to determine eligibility. Included papers were assessed against the two guidelines by one of three independent reviewers. The review protocol was registered with PROSPERO (CRD42023467970).

**Findings:**

The search identified 15,015 abstracts, from which 325 articles were included for evaluation. Most studies performed moderately on CLAIM, averaging a score of 28.9 ± 7.5 out of 53, but poorly on FUTURE-AI, averaging 5.1 ± 2.1 out of 30.

**Interpretation:**

Imaging-AI tools for STBT remain at the proof-of-concept stage, indicating significant room for improvement. Future efforts by AI developers should focus on design (e.g. defining unmet clinical need, intended clinical setting and how AI would be integrated in clinical workflow), development (e.g. building on previous work, training with data that reflect real-world usage, explainability), evaluation (e.g. ensuring biases are evaluated and addressed, evaluating AI against current best practices), and the awareness of data reproducibility and availability (making documented code and data publicly available). Following these recommendations could improve clinical translation of AI methods.

**Funding:**

10.13039/501100023452Hanarth Fonds, ICAI Lab, 10.13039/501100000272NIHR, EuCanImage.

**Research in context**

**Evidence before this study**

Research on the use of AI in diagnosing and predicting the outcomes of soft-tissue and bone tumours (STBT) is becoming more prevalent. However, the clinical adoption of AI methods in this field remains limited, highlighting a significant gap between AI development and its practical implementation in healthcare settings. Previous reviews focused on the accuracy and performance of published STBT tools, however, did not investigate the quality of research. Recent efforts have introduced guidelines with comprehensive criteria specifically designed for structured reporting and responsible development, deployment, and governance of trustworthy AI in healthcare.

**Added value of this study**

This review examines the methodological quality of published literature by assessing it against two best-practice guidelines, which were chosen to complement each other and cover a wide range of criteria. Aspects related to study quality, study design, and trustworthy and deployable AI, as assessed in this review using the CLAIM and FUTURE-AI guidelines, may be even more important factors than their performance for assessing their potential translation to the clinic. This review highlights what the field is doing well and where future research should focus. The review includes all research using AI methods investigating STBT, giving it a far wider scope than previous reviews. Furthermore, this is a fast-moving field, hence updates on previous reviews are required.

**Implications of all the available evidence**

Currently published AI methods are producing promising proof-of-concept results but are not ready for clinical application. This work highlights opportunities and provides recommendations for AI developers and clinical professionals for future research to drive clinical implementation.

## Introduction

Primary soft-tissue and bone tumours (STBT) are among the rarest neoplasms in humans, comprising both benign and malignant lesions. Malignant STBT, i.e. sarcoma, account for approximately 1% of all neoplasms.^1^ These tumours may occur at any age and almost any anatomical site, arising from cells of the connective tissue, including muscles, fat, blood vessels, cartilage, and bones.^2^ The rarity of STBT, along with their diverse subtypes and varied clinical behaviour, poses substantial challenges in accurate diagnosis and prognosis.

Radiological imaging (including nuclear medicine) is crucial in evaluating and monitoring STBT. Technological advancements in imaging modalities have led to a substantial increase data volume, along with a corresponding growth in the expertise required for its interpretation. The growing utilisation of radiological imaging and complexity of analysis has increased radiologists’ workload. Therefore, developing intelligent computer-aided systems and algorithms for automated image analysis that can achieve faster and more accurate results is crucial.^3^ For STBT, intelligent systems may help non-specialised radiologists in diagnosing rare cancers more effectively. Furthermore, an increased caseload is associated with higher interpretive error, which can be avoided with computer-aided diagnostic tools.^4^^,^^5^

Artificial intelligence (AI) has become increasingly prevalent in medical image analysis. Over the last 7 years, the number of FDA-approved medical imaging AI products for radiology has substantially increased.^6^ However, while medical imaging AI research in STBT has also substantially increased, there are no products developed for STBT among the FDA-approved list.^7^ Hence, instead of purely developing novel technological solutions, more research should focus on aligning with areas of unmet clinical need.

Therefore, a systematic assessment of current published research is necessary to identify the issues required to overcome the translational barrier. This systematic review aims to evaluate the existing literature on AI for diagnosis and prognosis of STBT using radiological imaging against two best practice guidelines; CLAIM and FUTURE-AI.^8^^,^^9^ CLAIM, endorsed by the Radiological Society of North America (RSNA), promotes comprehensive reporting of radiological research that uses AI. FUTURE-AI proposes ethical and technical standards to ensure responsible development, deployment, and governance of trustworthy AI in healthcare. Utilising both guidelines allows for comprehensive coverage of different aspects of AI research.^10^ Additionally, this review discusses opportunities for future research to bridge the identified gap between AI research and clinical use in STBT.

## Methods

This systematic review was prospectively registered with PROSPERO (CRD42023467970) and adheres to the Preferred Reporting Items for Systematic Reviews and Meta-analyses (PRISMA) 2020 guidelines.^11^ The full study protocol can be found online.^12^

### Search strategy and selection criteria

Medline, Embase, Web of Science core collection, Google Scholar, and Cochrane Central Register of Controlled Trials were systematically searched for relevant studies. All papers published before 27/09/2023 were included in the initial search; the starting date depended on the coverage of the respective database searched. The detailed search strategy is listed in Appendix 1. The literature search was conducted by the Medical Library, Erasmus MC, Rotterdam, the Netherlands. The database search was repeated on 17/07/2024 to update publications.

Inclusion criteria were: (1) original research papers published in peer-reviewed journals, and (2) studies focusing on radiology-based AI or radiomics characterisation of primary tumours located in bone and/or soft tissues for tasks related to diagnosis or prognosis, e.g. no pure segmentation studies. Exclusion criteria were: (1) animal, cadaveric, or laboratory studies, and (2) not written in English language.

**Fig. 1:**
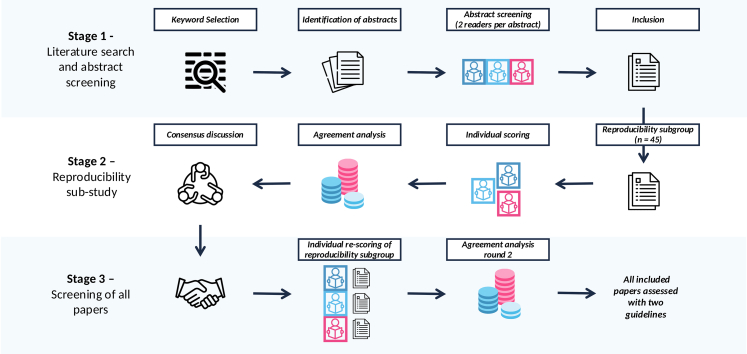
Reviewing methodology.

The complete reviewing methodology is illustrated in Fig. 1. Three independent reviewers participated in title-and-abstract screening (DS, MM, XW). Retrieved papers were randomly divided into three batches. Reviewers 1 and 2 reviewed one batch, Reviewers 1 and 3 reviewed a second batch, and Reviewers 2 and 3 reviewed the final batch. In cases where there were disagreements in the screening of an abstract, the third reviewer who was not initially involved in reviewing the specific abstract, adjudicated any conflicts.

### Data analysis

Each paper was scored according to CLAIM and FUTURE-AI guidelines. Checklists were developed based on each guideline. Blank checklists are available in Appendix 2. These guidelines were chosen for their complimentary nature and comprehensive coverage of clinical AI tool requirements.^10^

The CLAIM checklist was adapted from the checklist implemented by Si et al. to contain more detail in some of the more general checklist items.^8^^,^^13^^,^^14^ CLAIM consists of 44 items, covering the following sections: title, abstract, introduction, methods, results, discussion, and other information. The majority of items focus on the methods (30/44 items). The Methods section is further divided into the following subsections: Study design, Data, Ground truth, Data partition, Testing data, Model, Training, and Evaluation. Similarly, the Results section is divided into Data and Model performance. We further divided three items into twelve sub-items to provide more detailed information. These were: (4) Study objectives and hypotheses (4a and 4b), (7) Data sources (7a–d), and (9) Data preprocessing steps (9a–f). The adapted CLAIM checklist totalled 53 items.

The FUTURE-AI checklist was created from the FUTURE-AI guideline and contains 30 items.^9^ These items are split according to the six FUTURE-AI principles: Fairness (3), Universality (4), Traceability (6), Usability (5), Robustness (3), Explainability (2), and General (7). Additionally, FUTURE-AI specifies guidelines for AI tools at various machine learning technology readiness levels (TRL). It recommends (+) or strongly recommends (++) specific guidelines for tools at the proof-of-concept stage (Research) and for those intended for clinical development (Deployable).

All items in both sets of guidelines were scored between 0 and 1, with 0 meaning the item was not addressed, 0.5 meaning it was partially addressed (where relevant and only in FUTURE-AI) and 1 meaning it was fully addressed.

To ensure consistency between scores among reviewers, a subset of papers (n = 45) was selected for independent review by all three reviewers. The subset was selected by ordering the papers alphabetically based on the first author's name and choosing the first 45 papers from this order in the initial search. The number of disagreements for each item in either guideline was recorded, and inter-reader variability for each guideline was measured by calculating Fleiss' Kappa statistics (*κ)*.^15^ Fleiss kappa statistics were interpreted according to the guidance given by Fleiss et al., with a score 0–0.4 indicating poor agreement, 0.41–0.75 showing good agreement and >0.75 showing excellent agreement.^15^ To construct 95% confidence intervals (95% CI) for the inter-reader variability, 1000× bootstrap resampling was employed. The percentage agreement between all three reviewers was calculated for each item. Following this a consensus discussion was conducted between all three reviewers, allowing discussion and resolution of any systematic differences in interpretation and scoring of specific items. Next, each reviewer re-scored the same subset a second time, several weeks after the first scoring. Kappa statistics and percentage agreements were re-calculated.

After consensus, the remaining included papers were equally divided between the three reviewers and reviewed by a single reviewer. If a reviewer was uncertain how to score a paper, they consulted one or more of the other reviewers for confirmation or discussion. In addition to scoring the CLAIM and FUTURE-AI checklists, the following information was recorded for each paper: (1) year of publication, (2) journal of publication, (3) disease type investigated (soft tissue sarcoma, bone sarcoma, or gastrointestinal stromal tumour—GIST), (4) study design (retrospective or prospective—if a study used both retrospectively and prospectively acquired data it was recorded as being a prospective study), (5) outcome predicted (diagnosis, prognosis, or both), (6) imaging modality (MRI, CT, ultrasound, X-ray, PET-CT, PET-MRI, scintigraphy, or multiple imaging modalities), (7) data source (public, single centre, or multi-centre), and (8) availability of data and AI model source code.

The performance metrics of the corresponding AI models were collected for the top 20 performing papers, as determined by their combined CLAIM and FUTURE-AI scores, that performed external validation. Only the top 20 papers were included for this analysis as reported model performance cannot be reliably reproduced or considered clinically meaningful as low scoring studies lack methodological transparency or do not adhere to best scientific practices. For the same reason, only externally validated papers were selected to ensure robust assessment of model generalisability, reducing the risk of overfitting and dataset-specific bias, thus strengthening the clinical relevance of the reported findings.

### Statistics

The number of papers adhering to each item of CLAIM/FUTURE-AI was calculated. Descriptive statistics of how well papers scored in each (sub)section/principle were calculated, including mean, standard deviation (SD), maximum, and minimum score, as well as the mean and SD of the guideline adherence rate (AR), which is the score divided by the maximum achievable score.

### Role of funders

The funder of the study had no role in study design, data collection, data analysis, data interpretation, or writing of the report.

### Ethics

This study is a systematic review of published work and thus ethical approval was deemed unnecessary.

## Results

**Fig. 2:**
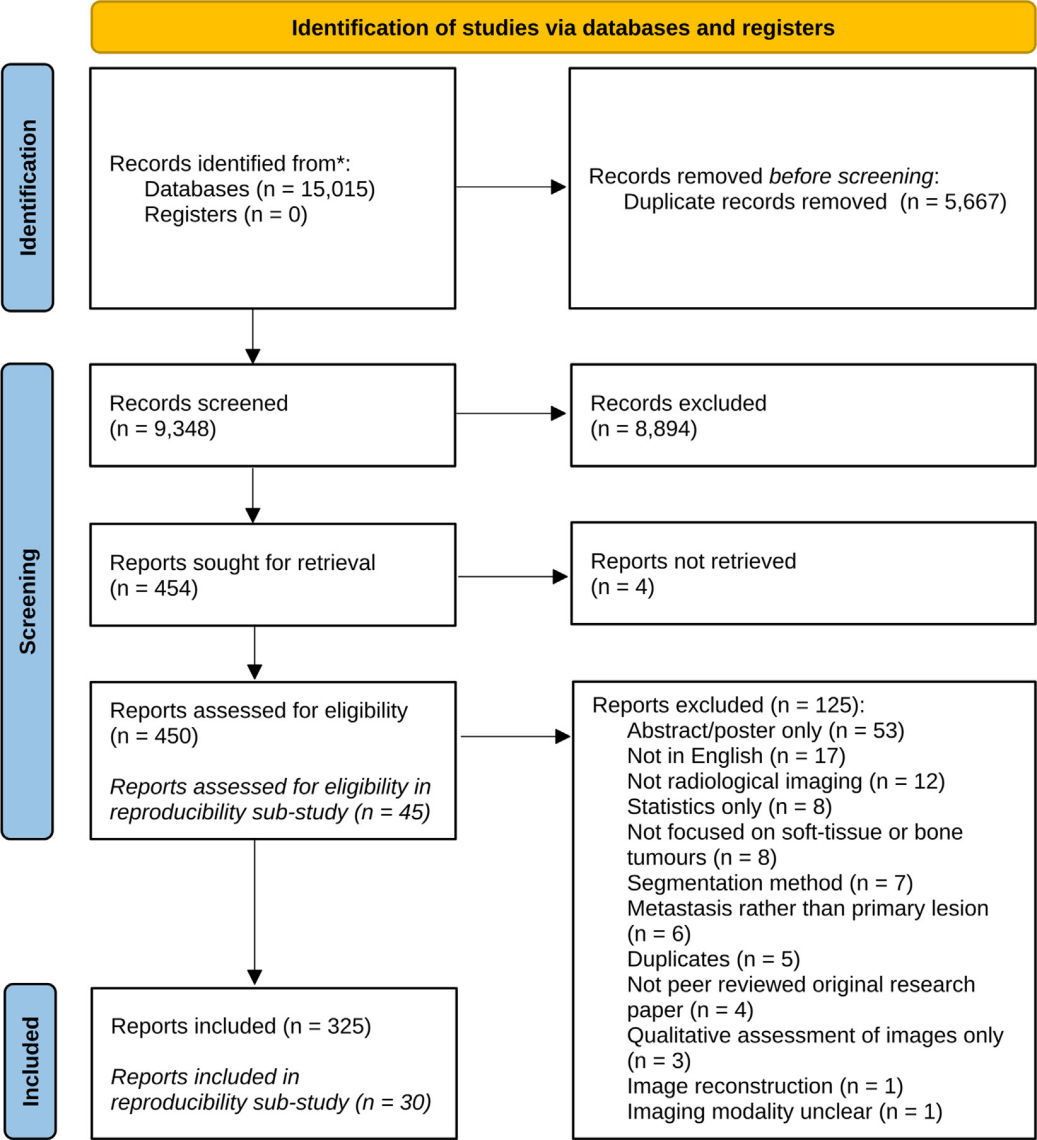
PRISMA flow diagram.

Database searches identified 15,015 published studies, with 5667 duplicates. After screening, 454 articles were retained for full-text review. After excluding 129 studies a total of 325 unique studies were included in the systematic review (Fig. 2). Fifteen of the excluded papers were part of the reproducibility subgroup, meaning 30 articles were independently reviewed by all reviewers. A complete reference list of the final 325 included papers is provided in Appendix 3. Main reasons for exclusion were focusing on different entities (e.g. renal cancer), no use of radiological imaging, or lacking AI-based analysis.

**Fig. 3:**
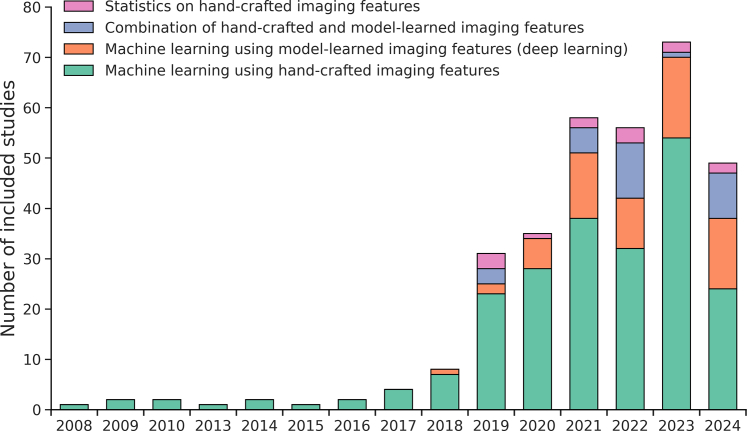
Number of included studies (n = 325) between 2008 and July 2024, color coded for the various AI methodologies used.

Included studies were published between 2008 and 2024, mostly in the last five years (Fig. 3). Of the 325 included studies, most AI methods used hand-crafted imaging features with machine learning (n = 221, 68%). Recently, more AI methods used model-learned imaging features (n = 62, 19%), i.e. deep learning, or a combination of model-learned and hand-crafted imaging features with machine learning (n = 29, 9%). Thirteen studies used hand-crafted imaging features without machine learning.

**Fig. 4:**
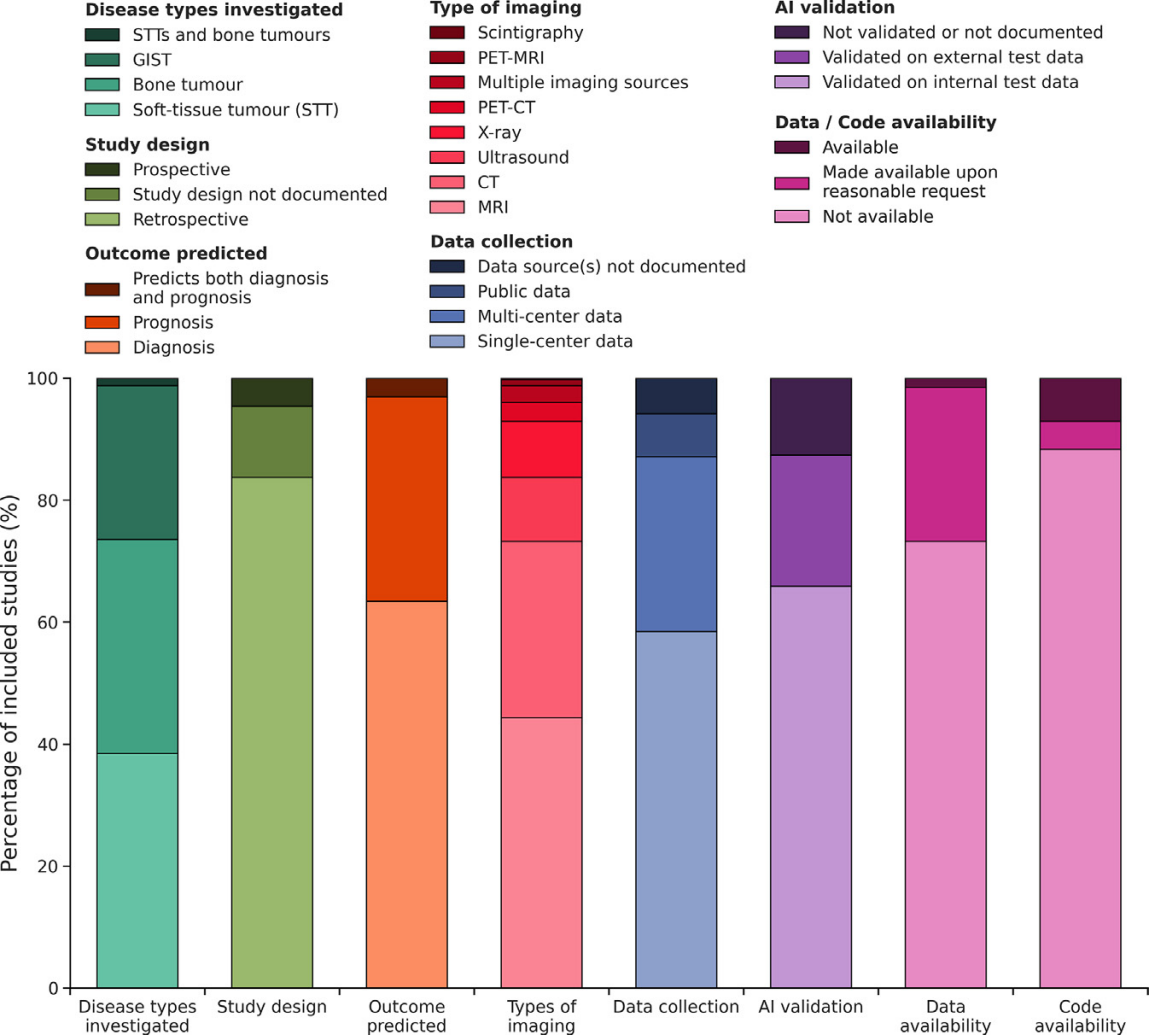
Characteristics of the studies included (n = 325) as percentages.

Study characteristics are illustrated in Fig. 4. Disease types included soft tissue tumours (n = 125, 38.5%), bone tumours (n = 114, 35.1%), and GIST (n = 82, 25.2%). Only four studies included both soft tissue and bone tumours (1.2%). Study design was mostly retrospective (n = 272, 83.7%), with fewer prospective studies (n = 38, 11.7%), and a minority where study design was not clearly documented (n = 15, 4.6%). The majority of reports focused on developing AI methods to predict diagnosis (n = 206, 63.4%), 109 (33.5%) evaluated prognosis, and 10 (3.1%) studied a combination of diagnosis and prognosis of the disease. Various radiological techniques were evaluated, with 144 (44.3%) studies using MRI, 94 (28.9%) CT, 34 (10.5%) ultrasound, 30 (9.2%) X-ray, 10 (3.1%) PET-CT, 3 (0.9%) PET-MRI, and 1 (0.3%) scintigraphy, and 9 (2.8%) multiple modalities. One-hundred-and-ninety (58.5%) studies collected data from a single centre, whereas 93 (28.6%) utilised imaging from multiple centres. Nineteen studies did not clearly document data provenance (5.8%). Furthermore, 23 (7.1%) studies used publicly available data from two sources (Table 1). AI methods were most often validated with separate internal test data (n = 214, 65.8%), and sometimes additionally with external test data (n = 70, 21.5%). Several AI methods were not validated with independent data or validation was not clearly documented (n = 41, 12.6%). Only 5 (1.5%) studies made data available, with 238 (73.2%) studies not providing or not specifying data availability, and 82 (25.2%) studies stating data would be made available on reasonable request. Similarly, AI source code to facilitate reproducibility was only made available in 23 (7.1%) studies, with 287 (88.3%) not providing or not specifying code availability, and 15 (4.6%) studies indicating code would be made available on reasonable request.

**Table 1:** Open-access datasets available with imaging for soft-tissue and bone tumours.

Data	Vallières et al. (2015)^16^	Starmans et al. (2021) [preprint^17^]
Origin	Canada	the Netherlands
Disease type	Various soft-tissue sarcoma (Extremities)	Various soft-tissue tumours
Imaging modality	MR and PET-CT	MR or CT
Number of patients	51	564
Additional data	Tumour segmentation and clinical outcome (lung metastasis)	Tumour segmentation and clinical outcome (phenotype)

Kappa statistics for inter-reader variability increased from 0.58 (95% CI: [0.55, 0.62]) to 0.68 (95% CI: [0.61, 0.75]) for CLAIM and FUTURE-AI before consensus discussion, to 0.80 (95% CI: [0.78, 0.83]) and 0.92 (95% CI: [0.88, 0.95]) after, showing excellent agreement (Supplementary Figures S1 and S2).

**Fig. 5:**
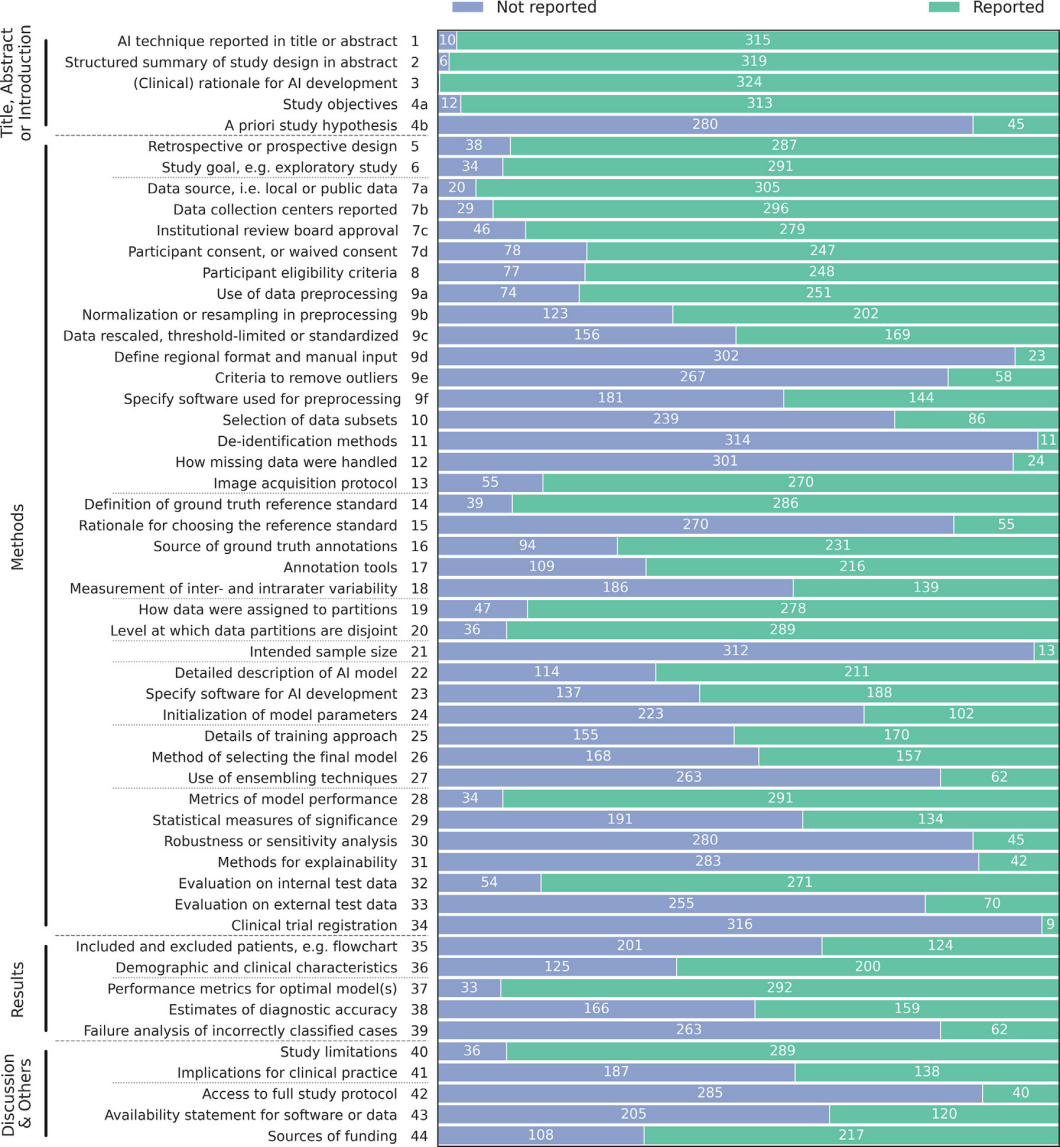
Reported and unreported criteria for the included studies (n = 325) from the Checklist for Artificial Intelligence in Medical Imaging (CLAIM). Gray bars between criteria within categories indicate subcategories.

**Fig. 6:**
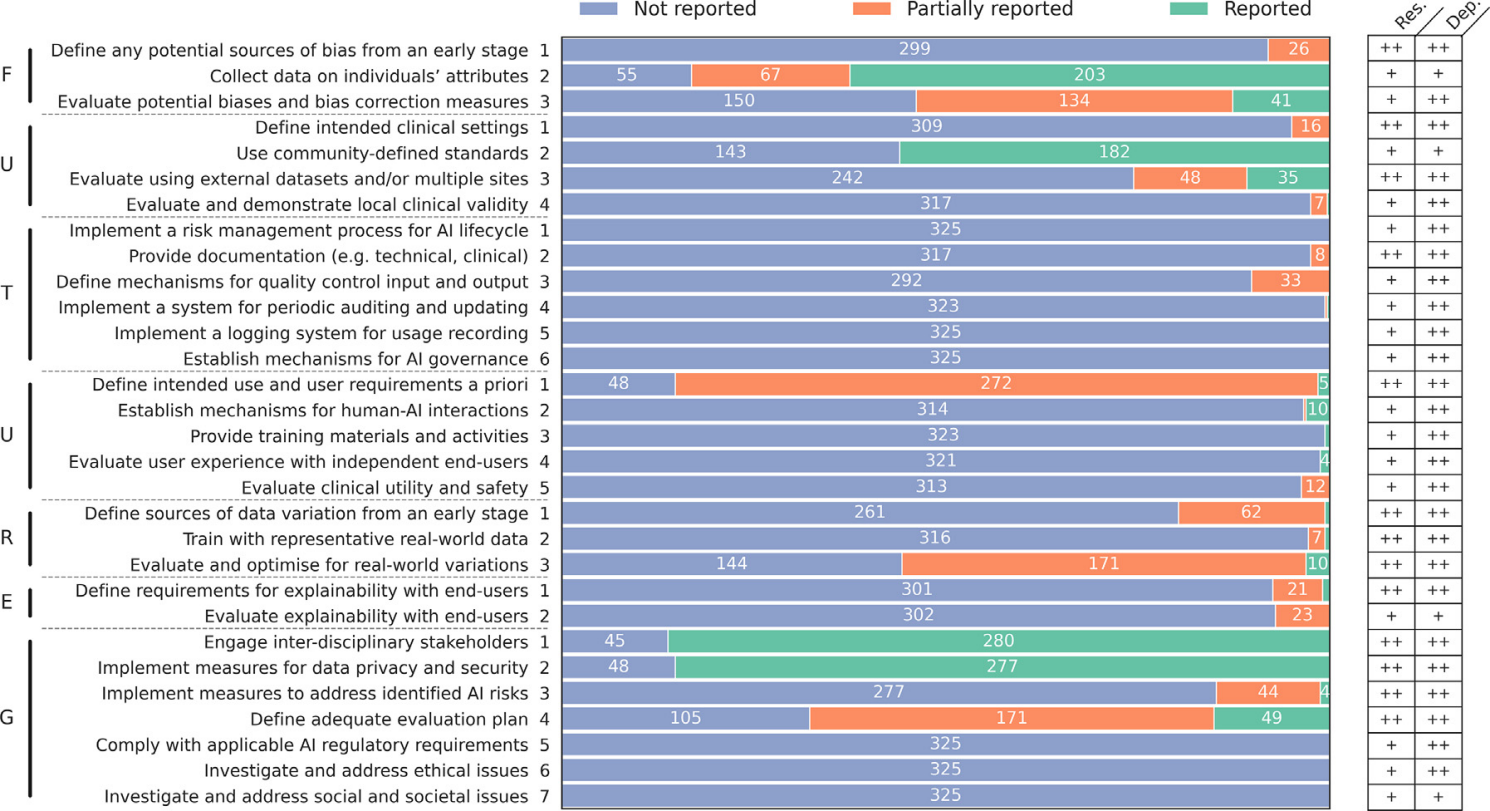
Scores of the included studies (n = 325) for each criterion from the FUTURE-AI international consensus guideline for trustworthy and deployable AI. For each criterion, expected compliance for both research (Res.) and deployable (Dep.) AI tools is reported. F, Fairness; U, Universality; T, Traceability; U, Usability; R, Robustness; E, Explainability; G, General recommendations.

**Supplementary Figure S7:**
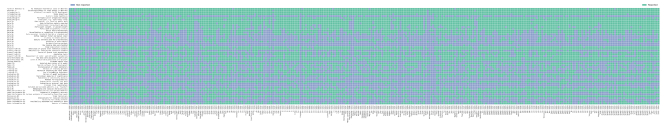


Individual scores for each item in Fig. 5 for CLAIM and Fig. 6 for FUTURE-AI. Section level scores are provided in Tables 2 and 3. Scores by year are available in Supplementary Figures S3 and S4, both showing an increasing trend. Scores by tumour type, method type, and outcome are available in Supplementary Figures S5 and S6, all showing no clear distinction between groups. Individual paper scores for each item are documented in Supplementary Figures S7 and S8, and are also available online as interactive figures and tables.^18^

**Table 2:** Summary scores of the included studies for each (sub)section of the Checklist for Artificial Intelligence in Medical Imaging (CLAIM).

(Sub)section	Maximum achievable score	Score (Mean ± SD)	Max score	Min score	Adherence rate (Mean ± SD)
**Title/Abstract**	**2.0**	**2.0 ± 0.2**	**2.0**	**0.0**	**98% ± 12%**
**Introduction**	**3.0**	**2.1 ± 0.4**	**3.0**	**0.0**	**70% ± 14%**
**Methods**	**38.0**	**19.8 ± 5.8**	**34.0**	**0.0**	**52% ± 15%**
Study design	2.0	1.8 ± 0.5	2.0	0.0	89% ± 24%
Data	15.0	8.0 ± 2.8	14.0	0.0	54% ± 18%
Ground truth	5.0	2.9 ± 1.4	5.0	0.0	57% ± 29%
Data partitions	2.0	1.7 ± 0.6	2.0	0.0	87% ± 30%
Testing data	1.0	0.0 ± 0.2	1.0	0.0	4% ± 20%
Model	3.0	1.5 ± 1.0	3.0	0.0	51% ± 33%
Training	3.0	1.2 ± 0.9	3.0	0.0	40% ± 31%
Evaluation	7.0	2.7 ± 1.3	6.0	0.0	38% ± 18%
**Results**	**5.0**	**2.6 ± 1.2**	**5.0**	**0.0**	**52% ± 24%**
Data	2.0	1.0 ± 0.8	2.0	0.0	50% ± 39%
Model performance	3.0	1.6 ± 0.8	3.0	0.0	53% ± 25%
**Discussion**	**2.0**	**1.3 ± 0.6**	**2.0**	**0.0**	**66% ± 32%**
**Other information**	**3.0**	**1.2 ± 0.9**	**3.0**	**0.0**	**39% ± 31%**
**Overall**	**53.0**	**28.9 ± 7.5**	**48.0**	**4.0**	**55% ± 14%**

Bold values represent CLAIM sections, while non-bold values indicate subsections.

**Table 3:** Summary scores of the included studies for each principle from the FUTURE-AI international consensus guideline for trustworthy and deployable AI.

Principle	Maximum achievable score	Score (Mean ± SD)	Max score	Min score	Adherence rate (Mean ± SD)
Fairness	3.0	1.1 ± 0.7	2.5	0.0	37% ± 22%
Universality	4.0	0.8 ± 0.7	3.0	0.0	20% ± 17%
Traceability	6.0	0.1 ± 0.2	1.0	0.0	1% ± 3%
Usability	5.0	0.5 ± 0.3	3.0	0.0	10% ± 7%
Robustness	3.0	0.4 ± 0.4	2.5	0.0	14% ± 12%
Explainability	2.0	0.1 ± 0.2	1.5	0.0	4% ± 12%
General	7.0	2.2 ± 0.8	3.5	0.0	32% ± 11%
Overall	30.0	5.1 ± 2.1	11.5	0.0	17% ± 7%

The included studies performed moderately on the CLAIM checklist, with a mean score of 28.9 out of 53 (SD: 7.5, min–max: 4.0–48.0, AR mean ± SD: 55% ± 14%). All items were reported at least once, but several were only reported in less than 15% of the papers (n ≤ 50 papers) including: define a study hypothesis at the design phase (CLAIM-4b, 13.8%), data de-identification methods (CLAIM-11, 3.4%), how missing data were handled (CLAIM-12, 8.2%), intended sample size and how it was determined (CLAIM-21, 4%), robustness or sensitivity analysis (CLAIM-30, 13.8%), methods for explainability or interpretability (CLAIM-31, 12.9%), registration number and name of registry (CLAIM-34, 2.8%), and documented where full study protocol can be accessed (CLAIM-42, 12.3%).

The included studies rarely adhered to FUTURE-AI, with a mean score of 5.1 out of 30 (SD: 2.1, min–max: 0–11.5, AR: 17% ± 7%). From the 30 items, 5 were never reported. Only 6 items were partially reported in over half of the reviewed papers (n > 162) including: collecting and reporting on individuals' attributes (Fairness-2, 83.1%), using community-defined standards (Universality-2, 56%), defining use and user requirements (Usability-1, 85.2%), engaging interdisciplinary stakeholders (General-1, 86.2%), implementing measures for data privacy and security (General-2, 85.2%), and defining an adequate evaluation plan (General-4, 67.7%).

Strongly recommended items by FUTURE-AI for proof-of-concept AI studies (Research), were reported more frequently than recommended items, with mean scores of 2.9 out of 12 (SD: 1.1, min–max: 0–7, AR: 24% ± 9%) and 2.3 out of 16 (SD: 1.2, min–max: 0–6.5, AR: 14% ± 8%), respectively. However, this trend was not observed in items intended to assess studies for clinical deployability (Deployable), where the mean scores were 3.8 out of 24 (SD: 1.7, min–max: 0–10, AR: 16% ± 7%) for strongly recommended items and 1.3 out of 4 (SD: 0.7, min–max: 0–3, AR: 33% ± 18%) for recommended items.

Performance measurements of the top 20 performing papers (ranked by the combined CLAIM and FUTURE-AI scores) which included external validation are provided in Table 4. These studies covered diverse disease types (soft-tissue tumours: n = 9, bone tumours: n = 8, GIST: n = 3), imaging modalities (MRI: n = 11, CT: n = 4, X-ray: n = 4, ultrasound: n = 1), outcomes (diagnosis: n = 12, prognosis: n = 7 and both diagnosis and prognosis: n = 1), and AI methodologies (machine learning model using a combination of hand-crafted and model-learned imaging features: n = 3; machine learning using model-learned features: n = 6; machine learning using hand-crafted imaging features: n = 11). Overall, AI methods demonstrated strong performance for their respective tasks, however there is a wide range in performance between models (AUC range: 0.64–0.95). However, most studies relied on a single centre for external validation (n = 12), and only a few included prospective validation (n = 2). These studies had a mean score of 40.4 out of 53 (SD: 3.0, AR mean ± SD: 76% ± 5.8%) for CLAIM and 8.4 out of 30 (SD: 1.6, AR mean ± SD: 28% ± 5.4%) for FUTURE-AI. Finally, among these top 20 studies, we explored potential associations between performance metrics, individual guideline scores, and three main study categories, as summarised in Supplementary Table S1. This showed no obvious differences in scores and performance metrics between any of the groups.

**Table 4:** Performance measurements of the top 20 performing papers, as determined by their combined CLAIM and FUTURE-AI scores, among those that performed external validation.

Author	Short description	Validation	Performance (Proportion, 95% CI)
Ye et al.^19^	A multi-task machine learning model using learned imaging features (deep learning) for the segmentation, detection, and differentiation of malignant and benign primary bone tumours, as well as bone infections, leveraging multi-modal inputs including T1-weighted MRI, T2-weighted MRI, and clinical data.	*External validation* 53 patients from 1 centre	AUC: 0.900 (0.773–1.000) Accuracy: 0.783 (0.581–0.903) Sensitivity: 0.756 (0.552–0.886) Specificity: 0.886 (0.764–0.950)
Dong et al.^20^	Machine learning model using learned imaging features (deep learning) differentiating gastrointestinal stromal tumours (GISTs) and leiomyomas on endoscopic ultrasonography.	*External validation* 241 patients from 1 centre *Prospective validation* 59 patients from 1 centre	*External validation* AUC: 0.948 (0.921–0.969) Accuracy: 0.917 (0.875–0.946) Sensitivity: 0.903 (0.834–0.945) Specificity: 0.930 (0.872–0.963) Precision: 0.919 (0.853–0.957) NPV: 0.915 (0.855–0.952) *Prospective validation (for GISTs and leiomyomas, respectively)* AUC: 0.865 (0.782–0.977) and 0.864 (0.762–0.966) Accuracy: 0.865 and 0.864 Sensitivity: 0.897 and 0.857 Specificity: 0.833 and 0.871 Precision: 0.839 and 0.857 NPV: 0.893 and 0.881
Xie et al.^21^	Machine learning model using learned imaging features (deep learning) to classify histological types of primary bone tumours on radiographs.	*External validation* 89 patients from 1 centre	AUC: 0.873 (0.812–0.920) Accuracy: 0.687 (0.614–0.783) Sensitivity: 0.572 (0.457–0.685) Specificity: 0.916 (0.893–0.938)
Xu et al.^22^	Machine learning model using a combination of hand-crafted and model-learned imaging features to differentiate between retroperitoneal lipomas and well-differentiated liposarcomas based on MDM2 status on contrast-enhanced CT.	*External validation* 63 patients from 2 centre	AUC: 0.861 (0.737–0.985) Accuracy: 0.810
Arthur et al.^23^	Machine learning model using hand-crafted imaging features classifying histological type and tumour grade in retroperitoneal sarcoma on CT.	*External validation* 89 patients from 8 centres^a^	*Histology and Grade* AUC: 0.928 and 0.882 Accuracy: 0.843 and 0.823 Sensitivity: 0.923 and 0.800 Specificity: 0.829 and 0.848 Precision: 0.480 and 0.865 NPV: 0.984, 0.778
Guo et al.^24^	Machine learning model using a combination of hand-crafted and model-learned imaging features to classify histological grade and predict prognosis of soft-tissue tumours on MRI.	*External validation* 125 and 44 patients from 2 centres *Prospective validation* 12 patients from 1 centre	*External validation (Centre 1 and Centre 2)* AUC: 0.860 (0.787–0.916) and 0.838 (0.696–0.932) Accuracy: 0.840 and 0.750 Sensitivity: 0.835 and 0.840 Specificity: 0.794 and 0.737 Hazard ratio: 4.624 (1.924–11.110) and 2.920 (0.603–14.150) *Prospective validation* AUC: 0.819 (0.501–0.974) Accuracy: 0.667 Sensitivity: 0.667 Specificity: 1.000
Gitto et al.^25^	Machine learning model using hand-crafted imaging features differentiating atypical cartilaginous tumour and grade II chondrosarcoma of long bones on MRI.	*External validation* 65 patients from 1 centre	AUC: 0.94 for atypical cartilaginous tumour and 0.90 for grade II chondrosarcoma Accuracy: 0.92 Sensitivity: 0.92 Precision: 0.92
Von Schaky et al.^26^	Machine learning model using hand-crafted imaging features to distinguish between benign and malignant bone lesions on radiography.	*External validation* 96 patients from 1 centre	AUC: 0.90 Accuracy: 0.75 (0.65–0.83) Sensitivity: 0.90 (0.74–0.98) Specificity: 0.68 (0.55–0.79) Precision: 0.57 (0.42–0.71) NPV: 0.94 (0.82–0.99)
Gitto et al.^27^	Machine learning model using hand-crafted imaging features differentiating atypical cartilaginous tumour and high-grade chondrosarcoma of long bones on radiography.	*External validation* 30 patients from 1 centre	AUC: 0.90 Accuracy: 0.80 Sensitivity: 0.89 Specificity: 0.67
Cao et al.^28^	Machine learning model using hand-crafted imaging features predicting the local recurrence after surgical treatment of primary dermatofibrosarcoma protuberans, based on MRI.	*External validation* 42 patients from 1 centre	AUC: 0.865 (0.732–0.998) for 3-year and 0.931 (0.849–1.00) for 5 year C-index: 0.866 (0.786–0.946)
Yang et al.^29^	Machine learning model using hand-crafted imaging features predicting progression-free survival after imatinib therapy in patients with liver metastatic gastrointestinal stromal tumours on multi-sequence MRI.	*External validation* 45 patients from 1 centre	AUC: 0.766 for 1-year, 0.776 for 3-year, and 0.893 for 5-year C-index: 0.718 (0.618–0.818)
Chen et al.^30^	Machine learning model using hand-crafted imaging features predicting pathologic response to neoadjuvant chemotherapy (NAC) in patients with osteosarcoma on MRI.	*External validation* 34 patients from 3 centres	AUC: 0.842 (0.793–0.883) Accuracy: 0.765 ± 0.020^b^ Sensitivity: 0.739 ± 0.032^b^ Specificity: 0.909 ± 0.026^b^
Liang et al.^31^	Machine learning model using a combination of hand-crafted and model-learned imaging features for predicting lung metastases in patients with soft-tissue sarcoma on MRI.	*External validation* 126 patients from 2 centre	AUC: 0.833 (0.732–0.933) Accuracy: 0.897 Sensitivity: 0.474 Specificity: 0.972 Precision: 0.750 NPV: 0.912
Kang et al.^32^	Machine learning model using learned imaging features (deep learning) to predict preoperative risk of gastrointestinal stromal tumours on CT.	*External validation* 388 patients from 1 centre	*Low-malignant, intermediate-malignant, and high-malignant* AUC: 0.87 (0.83–0.91), 0.64 (0.60–0.68), and 0.85 (0.81–0.89) Accuracy: 0.81 (0.77–0.85), 0.75 (0.71–0.79), and 0.77 (0.73–0.81) Sensitivity: 0.72 (0.64–0.79), 0.24 (0.14–0.34), and 0.79 (0.73–0.85) Specificity: 0.86 (0.83–0.90), 0.86 (0.82–0.90), and 0.75 (0.70–0.81)
He et al.^33^	Machine learning model using learned imaging features (deep learning) for classification of benign, intermediate or malignant primary bone tumours on radiography.	*External validation* 291 patients from 2 centre	AUC: 0.877 (0.833–0.918) benign vs not benign and 0.916 (0.877–0.949) malignant vs not malignant Accuracy: 0.734
Peeken et al.^34^	Machine learning model using hand-crafted imaging features from different timepoints (delta radiomics) predicting pathologic complete response to neoadjuvant therapy in high grade soft tissue sarcoma of trunk and extremity, based on MRI.	*External validation* 53 patients from 1 centre	AUC: 0.75 (0.56–0.93) Accuracy: 0.86 Balanced accuracy: 0.57 Sensitivity: 0.20 Specificity: 0.95 Precision: 0.33 NPV: 0.90
Foreman et al.^35^	Machine learning model using hand-crafted imaging features predicting the MDM2 gene amplification status in order to differentiate between atypical lipomatous tumours (ALT) and lipomas on MRI.	*External validation* 50 patients from 1 centre	AUC: 0.88 (0.85–0.91) Accuracy: 0.76 Sensitivity: 0.70 Specificity: 0.81
Spraker et al.^36^	Machine learning model using hand-crafted imaging features predicting overall survival of grade II and III soft-tissue tumours on MRI.	*External validation* 61 patients from 1 centre	Sensitivity: 0.79 Specificity: 0.68 C-index: 0.78 Hazard ratio: 2.4
Fradet et al.^37^	Machine learning model using a combination of hand-crafted and model-learned imaging features predicting malignancy for lipomatous soft-tissue lesions on MRI.	*External validation* 60 patients from 35 centres	AUC: 0.80 Specificity: 0.63
Gitto et al.^38^	Machine learning model using hand-crafted imaging features differentiating atypical cartilaginous tumours and high-grade chondrosarcomas of long bones on CT.	*External validation* 36 patients from 1 centre	AUC: 0.784 Accuracy: 0.75

AUC, area under the curve; CI, confidence interval; NPV, negative predictive value.

a AI development centre was also included as one of the eight external validation centres.

b Values are mean ± standard deviation.

## Discussion

This work has systematically identified and summarised radiological imaging-AI research on STBT and conducted comprehensive evaluation of published literature against two best-practice guidelines: CLAIM and FUTURE-AI. These guidelines were developed to ensure that AI tools target unmet clinical needs, are transferrable, generalisable, and can be used in real-world clinical practice. Analysis revealed a rapid increase in experimental AI tools for imaging-based STBT evaluation over the past five years. Studies performed moderately against CLAIM (28.9 ± 7.5 out of 53) and poorly against FUTURE-AI evaluations (5.1 ± 2.1 out of 30). The poor results in FUTURE-AI are expected as these guidelines are recent and set high requirements. Several papers do show higher scores in both CLAIM and FUTURE-AI (Table 4) and show promising results in external validation cohorts (AUC range: 0.784–0.948). However, the highest scoring paper achieved only a 11.5 out of 30 in FUTURE-AI, highlighting room for improvement. These results suggest that while progress has been made in developing AI tools for STBT, most studies are still at the proof-of-concept stage and there remains substantial room for improvement to guide future clinical translation. Summaries of the authors’ recommendations can be found at the end of the discussion, focusing on five key topics: design, development, evaluation, reproducibility, and data availability.

In the design stage, several critical aspects warrant more attention. Intended clinical settings (Universality-1) and prior hypotheses (CLAIM-4b) should be reported. On a positive note, over 85% of studies involved interdisciplinary teams (Usability-1, General-1), which is recommended for effective AI tool development.^9^ However, most studies did not comprehensively identify possible sources of bias at an early stage (Fairness-1, Robustness-1), which could limit the applicability of these AI tools. To overcome this, interdisciplinary stakeholders should work together from the design stage to identify the clinical role of the AI tool, ensure it integrates into the clinical workflow, and any possible sources of bias.

In the development stage, studies generally reported dataset source and conducted research with appropriate ethical approvals (CLAIM-7). However, almost half of studies did not assess biases during AI development (Fairness-3) and very few studies trained with representative real-world data (Robustness-2), which can hinder the transferability of AI tools, especially given the highly heterogeneous imaging characteristics of STBT. Another notable gap is a lack of focus on explainability and traceability. Few studies addressed items under FUTURE-AI Explainability (1–2) and Traceability (1–3), similar shortcoming was observed in the CLAIM checklist (CLAIM-31). While accuracy is crucial in medical practice, it is often argued that AI methods should go beyond pure performance metrics by addressing other factors such as prediction uncertainties, explaining their outputs, and providing clinicians with detailed information.^39^ For AI tools to be effective in clinical decision-making, explainability is vital to ensure clinicians understand and can trust the AI's reasoning.^40^ Additionally, to assist with AI development, research should build on previous work where possible. To assist with this, researchers should continue to adhere to community-defined standards, which is currently done in over half of the reviewed papers, and ensure their code is available. This review shows that almost all included studies developed new models rather than adapting or enhancing existing ones, even when promising results were achieved. Finally, it is integral that AI tools are easy for the end-user to use in the clinical workflow, however only two studies developed a graphical user interface for user experience testing (Usability-3).^20^^,^^41^

Regarding evaluation, while over 85% of studies adopted relevant metrics and reported AI algorithm performance (CLAIM-28 and 37), only 22% conducted external validation (CLAIM-33), and most used single-institute datasets (Universality-3). Furthermore, several studies lacked thorough internal validation (Robustness-3, General-4). AI tools should be tested against independent external data, ideally from multiple sources, to assess the tool's universality and prevent site-specific bias. Accuracy metrics should also be compared against current best practice (i.e. compared to radiologists) to ensure AI tools offer improvements in outcomes. Less than 20% of studies reported failure analysis or incorrectly classified cases (CLAIM-39). Including failure analysis is crucial to identify potential pitfalls, helping users understand when it is appropriate to use the tool. Developers should also ensure that the tool is robust against the biases identified during the design stage.

Regarding reproducibility, most studies fail to provide adequate materials (code, model, and data) to reproduce published results. Only around 10% of studies offered a full study protocol, including comprehensive methodology or code. Making protocols and code available enables others to reproduce the study across multiple steps, such as data preprocessing, ground truth acquisition, model construction, and training procedure. The lack of accessible and reproducible AI research in STBT could impede the adoption of these tools, as sarcoma centres may struggle to reproduce the tools performance locally. Adhering to guidelines such as CLAIM could enhance the quality and accessibility of these protocols.

Regarding data availability, there is a lack of freely accessible annotated imaging datasets of STBT, as highlighted in Table 1. Although 25% of published research stated that data used was available by request, a recent study by Gabelica et al. (2022) investigating compliance with data sharing statements showed a response rate of 14%, with only 6.8% supplying the data.^42^ One challenge in creating these datasets is the time required and the need for an easy-to-use format. Structured and standardised reporting in clinical practice could help reduce the effort needed for retrospective data collection. However, AI developers often struggle to collate data themselves, especially since STBT are rare and only treated at tertiary sarcoma centres. This underscores the importance of collaborating with clinical professionals. Increasing data availability would accelerate AI tool development and allow for external validation of models. Potential solutions include hosting “grand challenges” where clinicians provide data for AI developers to tackle a real-world clinical problem, or employing federated learning, which has proven effective for training AI models on rare tumours across international networks.^43^, ^44^, ^45^

Several reviews described the use of AI or radiomics in STBT management.^46^, ^47^, ^48^, ^49^ This study expands and complements these previous reviews, including a substantially larger volume of included publications (325 vs 21–52 reports) primarily due to our extended scope and search strategy, including benign soft-tissue tumours, bone tumours, and a broad range of AI methods (i.e. not limiting to radiomics with hand-crafted features). Furthermore, most previous reviews only examined the accuracy and performance of published AI tools in the field; the current systematic review instead examined the methodological quality of published literature by assessing this against best-practice guidelines. The only other systematic reviews that, to the authors knowledge, have assessed quality of AI research in radiology imaging for STBT are Crombé et al. (2020) (52 studies) and De Angelis et al. (2024) (49 studies), both scoring against the Radiomics Quality Score (RQS).^46^^,^^47^ In this study, different scoring systems were deliberately chosen as CLAIM and FUTURE-AI are independent but complementary guidelines, providing a broader assessment of overall quality than using only one.^10^ FUTURE-AI allows assessment of trustworthiness, deployability, and translation to clinical practice, while CLAIM guidelines, which are endorsed by the RSNA, ensures that studies are reported according to a standard set of information especially designed for medical imaging AI. Findings indicate that the field continues to produce promising proof-of-concept results but is not ready to make the jump to clinical application. This agrees with earlier work in the field.

To better understand the relationship between adherence to reporting guidelines and model performance, we examined the top 20 studies with the highest combined CLAIM and FUTURE-AI scores. Our analyses suggest that no particular subfield demonstrates consistently superior performance, with reported metrics varying widely—even among similar models. This underscores the need for further external validation and standardization. Whilst some studies show promising results, the overall heterogeneity highlights the complexity of AI performance assessment.

Subgroup analysis in which CLAIM and FUTURE-AI scores were investigated by tumour type, method type and outcome, showed no obvious differences between groups although papers performing statistics on hand crafted features scored worse than studies which used some form of machine learning. This is not surprising as the guidelines we chose focus on the use of AI. There was a general trend for a small increase in scores for both guidelines over time. This implies that whilst the quality of AI-based research is improving over time no field assessed in this review is ahead than any other.

There are limitations to this study. First, due to the large volume of literature, most papers were scored by a single reviewer. However, a sub-group of papers were scored by three reviewers followed by consensus analysis, showing excellent agreement, and reviewers remained in discussion if they had doubts about how best to score a paper for a particular category. Two or more reviewers per paper might have provided more robust results but would have required a significant time investment for likely only marginal gains. Secondly, in the reproducibility study with subgroups, papers were selected by alphabetical order based on the first author's name. While this approach introduces a degree of randomness, a fully randomised selection process would have been more robust to minimise potential biases. Third, future studies could benefit from integrating additional scoring guidelines such as APPRAISE AI, TRIPOD-AI, or RQS, alongside CLAIM and FUTURE-AI.^50^, ^51^, ^52^ Integrating more guidelines may provide a more comprehensive evaluation of both reporting adherence and study quality.

In conclusion, this review discusses the growing volume of published work evaluating imaging-related AI tools to aid in diagnosis, prognosis, and management of soft tissue and bone tumours. The top performing papers, as determined by both guidelines, may represent encouraging steps toward bringing AI in radiology closer to clinical translation, however even these have some limitations. The identified limitations of the reviewed studies with respect to CLAIM and FUTURE-AI guidelines will need to be addressed before such tools can translate into the clinical domain. Several opportunities have been identified and the authors’ recommendations to promote translation of AI methods into clinical practice are summarised below. Addressing these points may help drive clinical adoption of AI tools into the radiology workflow in a responsible and effective way.

### Recommendations to promote clinical translation of AI methods for soft-tissue and bone tumours

#### Design

• Interdisciplinary stakeholders should define: (A) the unmet clinical need, (B) the intended use of AI, (C) intended clinical setting in which AI should operate, (D) the end-user requirements, (E) how AI would operate in clinical workflow.
• Possible types and sources of bias (e.g. sex, age, ethnicity, socioeconomics, geography) should be identified at the early design stage.

#### Development

• Data used for AI development should reflect real-world data used in the intended clinical setting or preferably retrieved from the clinical setting. Additionally, sources of variation and potential biases should be investigated early in the development process.
• Explainability of AI methods should be developed and implemented in a way that it is possible to understand why an AI tool has arrived at its predictions.
• AI development should build on previous work by: (A) adhering to community-defined standards, and (B) considering previous existing methods by validating or improving them whenever possible.
• Ensure that AI tools are easy for the end-user to use in a clinical setting.

#### Evaluation

• AI tools should be evaluated using independent external test data. Limits on universality of the external test sets should be discussed.
• AI tools should be evaluated against current best practices, e.g. classification by radiologist or histology results from biopsy, and evaluated with intended end-users.
• Failure analysis of incorrect classified cases should be conducted.
• The robustness and sensitivity to variations and biases in data, identified prior to AI development, should be thoroughly investigated.

#### Reproducibility

• Code should be made publicly available, readable, usable and traceable to increase confidence in the method.
• The Methods section should comprehensively cover all aspects of AI development, including: (A) data preprocessing, (B) ground truth acquisition, (C) a detailed description of the AI methodology, and (D) the training procedures. To this end, the Checklist for Artificial Intelligence in Medical Imaging (CLAIM) could be followed.

#### Data availability

• Structured and standardised reporting should be introduced in clinical practice to limit the manual work required in retrospective data collection.
• Tertiary sarcoma centres should collect labelled data and make this publicly available, preferably in the context of a “grand challenge”, while protecting patient details and respecting privacy.
• To protect patient privacy and avoid excessive data-sharing, researchers could work together using a federated learning approach.

## Contributors

D.J.S., M.M., X.W.: conceptualisation, data curation, formal analysis, investigation, methodology, project administration, visualisation, writing—original draft, and writing—review & editing; S.K., M.P.A.S: conceptualisation, investigation, methodology, supervision, writing—review & editing; A.F.S., P.R., E.H.G.O., J.J.V., R.H., K.L., D.F.H., G.J.L.H.L., C.V., D.J.G., W.J.N.: methodology, supervision, writing—review & editing; S.K., M.P.A.S., M.M., E.H.G.O., J.J.V., C.V., D.J.G., W.J.N.: funding acquisition. All authors read and approved the final version of the manuscript. D.J.S, M.M., X.W., S.K., M.P.A.S. have accessed and verified the data. D.J.S, M.M., X.W. have contributed equally. S.K. and M.P.A.S. have contributed equally.

## Data sharing statement

Empty checklists for this review are included in the supplementary material. All data collected and analysed in this study are available online.^18^ A website (https://douwe-spaanderman.github.io/AI-STTandBoneTumour-Review/) with interactive figures and tables with scores for each paper is also available online.

## Declaration of interests

WJN is the founder of Quantib and was scientific lead until 31-1-2023. JJV received a grant to institution from Qure.ai/Enlitic; consulting fees from Tegus; payment to institution for lectures from Roche; travel grant from Qure.ai; participation on a data safety monitoring board or advisory board from Contextflow, Noaber Foundation, and NLC Ventures; leadership or fiduciary role on the steering committee of the PINPOINT Project (payment to institution from AstraZeneca) and RSNA Common Data Elements Steering Committee (unpaid); phantom shares in Contextflow and Quibim; chair scientific committee EuSoMII (unpaid); chair ESR value-based radiology subcommittee (unpaid); member editorial board European Journal of Radiology (unpaid). SK and EHGO are scientific directors of the ICAI lab “Trustworthy AI for MRI”, a public-private research program partially funded by General Electric Healthcare. The other authors do not have any conflicts of interest.

## Figures and Tables

**Supplementary Figure S8:**