# Supplementary material for: AI in radiological imaging of soft-tissue and bone tumours: a systematic review evaluating against CLAIM and FUTURE-AI guidelines
Source: eBioMedicine. 2025 Mar 20;114:105642. doi: 10.1016/j.ebiom.2025.105642 (PMC11976239; doi:10.1016/j.ebiom.2025.105642)
Supplement: Appendix 1 [file mmc1.docx]

**Appendix 1: Search strategy**

| **Database searched** | **Platform** | **Coverage period** |
| --- | --- | --- |
| Medline ALL | Ovid | 1946 – 07/2024 |
| Embase | Embase.com | 1971 - 07/2024 |
| Web of Science Core Collection* | Web of Knowledge | 1975 - 07/2024 |
| Cochrane Central Register of Controlled Trials** | Wiley | 1992 - 07/2024 |
| Additional Search Engines: Google Scholar*** | | |
| **Total** | | |

*Science Citation Index Expanded (1975- 07/2024); Social Sciences Citation Index (1975- 07/2024); Arts & Humanities Citation Index (1975- 07/2024); Conference Proceedings Citation Index- Science (1990- 07/2024); Conference Proceedings Citation Index- Social Science & Humanities (1990- 07/2024); Emerging Sources Citation Index (2005- 07/2024)

** Manually deleted abstracts from trial registries

***Google Scholar was searched via "Publish or Perish" to download the results in EndNote.

No other database limits were used than those specified in the search strategies

**Embase**

('artificial intelligence'/exp OR 'machine learning'/exp OR 'pattern recognition'/exp OR 'radiomics'/exp OR (CNN OR (artificial* NEAR/3 intelligen*) OR ((machine OR deep) NEAR/3 learning) OR (neural* NEAR/3 network*) OR (classification* NEAR/3 (algorithm OR binary OR multiclass OR multilabel)) OR (classifier*) OR (data-mining*) OR (feature NEAR/3 detection*) OR (feature* NEAR/3 (extraction OR learning OR ranking OR selection OR analysis OR fusion*)) OR (k-nearest* NEAR/3 neighbo*) OR (kernel* NEAR/3 method*) OR (learning* NEAR/3 algorithm*) OR (least* NEAR/3 absolute* NEAR/3 shrinkage* NEAR/3 selection* NEAR/3 operator*) OR (Markov* NEAR/3 model*) OR (memristor*) OR (network* NEAR/3 learning*) OR (perceptron*) OR (radial* NEAR/3 basis* NEAR/3 function*) OR (random* NEAR/3 forest*) OR (recursive* NEAR/3 feature* NEAR/3 elimination*) OR (recursive* NEAR/3 partitioning*) OR (support* NEAR/3 vector* NEAR/3 machine*) OR ((recognition* OR detection* OR classification* OR predict* OR comput* OR diagnos*) NEAR/3 (algorithm* OR network* OR computer-aided* OR automatic* OR automated*)) OR bayesian* OR radiomic* OR pattern-recognit* OR ((AI) NEXT/1 (tool* OR model*))):ab,ti,kw OR AI:ti) **AND** ('musculoskeletal tumor'/exp OR 'bone cyst'/exp OR 'fibrous dysplasia'/exp OR 'lipoma'/exp OR 'hibernoma'/exp OR 'mesenchymoma'/exp OR 'lymphoma'/exp OR 'histiocytosis'/exp OR 'sinus histiocytosis'/exp OR 'sarcoma'/exp OR 'soft tissue tumor'/exp OR 'nerve tumor'/exp OR 'lymphangioma'/exp OR 'lipoblastoma'/exp OR 'ganglion cyst'/exp OR (GCTB OR DDLS OR GIST OR GISTs OR ((soft-tissue* OR adipos*-tissue* OR glomus* OR gastrointest-stroma* OR gastr*-intest*-stroma* OR spinal* OR rib OR skull OR sternal* OR tibial* OR sacrum* OR jaw OR maxillar* OR mandibular* OR odontogenic* OR connective-tissue* OR subcutan*-tissue* OR vein* OR muscle* OR musculoskeletal* OR bone* OR benign-notochordal-cell OR fibrous* OR osteoblast* OR osteoclast* OR synov* OR granular-cell* OR cartilag* OR joint* OR femoral* OR humerus* OR lympho* OR rhabdoid OR non-ossifying OR extramedullary-myeloid* OR atypical-lipmatous* OR nerve* OR giant-cell* OR schwann-cell* OR desmoplastic* OR myofibroblastic*) NEAR/3 (tumor* OR tumour* OR cancer* OR neoplas* OR maligna* OR lesion* OR plasmacytom* OR metasta*)) OR ((vascular* OR arter* OR vessel* OR venal*) NEXT/1 (tumor* OR tumour* OR cancer* OR neoplas* OR maligna* OR lesion* OR plasmacytom* OR metasta*)) OR ((lymph-node*) NEAR/3 (tumor* OR tumour* OR cancer* OR neoplas* OR maligna* OR lesion* OR plasmacytom*)) OR adamantin* OR plasma-cell-granulom* OR glomangiom* OR myoma* OR desmoid* OR Bessel-Hagen OR diaphyseal-aclas* OR ((subungual OR multipl* OR dysplas* OR familial*) NEAR/3 (exosto*)) OR osteocyst* OR ecchondrosis-ossificans OR chondrodysplasia OR adenosarcom* OR sarcom* OR gliosarcoma* OR adenosarcom* OR osteosarcom* OR chondrosarcom* OR chondrom* OR enchondrom* OR chondroblastom* OR chondromatosis* OR osteom* OR osteoblastom* OR osteochondrom* OR maffucci* OR hemangiom* OR haemangiom* OR hemangioendotheliom* OR angiosarcom* OR bone-cyst* OR osseous-cyst* OR intraosseous-gangli* OR intra-osseous-gangli* OR ganglion-cyst* OR jaw-cyst* OR subchondral-cyst* OR chordom* OR synoviom* OR ((fibro*) NEAR/2 (dysplas* OR dystroph* OR osteodys*)) OR cherubism* OR osteofibrous-dysplasi* OR lipom* OR angiolipom* OR angiom* OR lipomatos* OR fetal-lipoma* OR Bannayan OR fatty-kidney OR fatty-pancreas* OR hibernom* OR mesenchym* OR adamantinom* OR hodgkin* OR erdheim-Chester* OR chester-erdheim* OR eosinophil*-granulom* OR histiocytos* OR dorfman-rosai-disease* OR nora-s-lesion* OR chondromesenchymal-hamartoma-of-chest-wall* OR lymphom* OR fibroma* OR osteoclastom* OR histioblastom* OR histiosarcom* OR leiomyosarcom* OR angioendotheliom* OR angioendotheliosarcom* OR hemangiosarcom* OR haemangiosarcom* OR haemangioendotheliom* OR hemangio-endotheliosarcom* OR hemangioendotheliom* OR hemangioendotheliosarcom* OR hemangio-endotheliom OR haemangio-endotheliom* OR lymphangiosarcom* OR Stewart-Treves OR rhabdomysarcom* OR myxofibrosarcom* OR myxosarcom* OR myofibrom* OR myofibroblastom* OR synoviom* OR myxom* OR myopericytom* OR fibrosarcom* OR fibroadenosarcom* OR dermatofibrosarcom* OR neurofibrosarcom* OR chloroma* OR extramedullary-leukaemia* OR extramedullary-leukemia* OR leukosarcom* OR liposarcom* OR neurom* OR perineurom* OR ganglionneurom* OR neurilemom* OR neurofibrom* OR neurothekeom* OR leiomyom* OR rhabdomyom* OR elastofibroma* OR lymphangiom* OR hemangiopericytom* OR haemangiopericytom* OR pericytom* OR myopericytom* OR glomangiopericytom* OR lipoblastom* OR schwannom* OR neurilemmom* OR neurinom* OR neurolemmom* OR neurilemom* OR neurolilemmon* OR ((pigment* OR arthritis*) NEAR/3 (villonodular* OR villous*)) OR ((arthritis*) NEAR/3 (pigment* OR schueller*)) OR ((synovitis*) NEAR/3 (pigment* OR dendritic* OR villonodular*)) OR lymphosarcom* OR reticulosarcom* OR rhabdomyosarcom* OR ameloblastom* OR myosarcom* OR fibrosarcom* OR myoblastom* OR fibrous-histiocytom* OR histiomatos* OR reticulohistiocyt*):ab,ti,kw) **AND** ('radiomics'/exp OR 'radiogenomics'/exp OR 'diagnostic imaging'/de OR 'radiodiagnosis'/exp OR 'nuclear magnetic resonance imaging'/exp OR 'diffusion coefficient'/de OR 'diffusion weighted imaging'/de OR 'Doppler flowmetry'/de OR 'echography'/exp OR (radiogenomic* OR ((radio OR radiat*) NEXT/1 (genomic* OR diagnos*)) OR radiomic* OR ((diagnos* OR medical*) NEAR/3 imag*) OR radio-genomic* OR radiomic* OR (diagnos* NEAR/3 imag*) OR radiodiagnos* OR ((comput* OR positron) NEAR/3 tomogra*) OR spect OR ct OR pet OR mri OR (magnetic NEAR/3 resonance) OR ((nuclear OR mr OR multimodalit*) NEAR/3 imaging*) OR rontgen OR roentgen OR ultraso* OR scintigra* OR (diffusion* NEAR/3 (coefficient* OR weighted OR tensor)) OR dwi OR dti OR Doppler OR echogra*):ab,ti,kw) NOT ([Conference Abstract]/lim AND [1800-2020]/py) NOT ('case report'/de OR (case-report):ti) NOT ((animal/exp OR animal*:de OR nonhuman/de) NOT ('human'/exp))

**Medline**

(exp Artificial Intelligence/ OR exp Machine Learning/ OR Pattern Recognition, Automated/ OR (CNN OR (artificial* ADJ3 intelligen*) OR ((machine OR deep) ADJ3 learning) OR (neural* ADJ3 network*) OR (classification* ADJ3 (algorithm OR binary OR multiclass OR multilabel)) OR (classifier*) OR (data-mining*) OR (feature ADJ3 detection*) OR (feature* ADJ3 (extraction OR learning OR ranking OR selection OR analysis OR fusion*)) OR (k-nearest* ADJ3 neighbo*) OR (kernel* ADJ3 method*) OR (learning* ADJ3 algorithm*) OR (least* ADJ3 absolute* ADJ3 shrinkage* ADJ3 selection* ADJ3 operator*) OR (Markov* ADJ3 model*) OR (memristor*) OR (network* ADJ3 learning*) OR (perceptron*) OR (radial* ADJ3 basis* ADJ3 function*) OR (random* ADJ3 forest*) OR (recursive* ADJ3 feature* ADJ3 elimination*) OR (recursive* ADJ3 partitioning*) OR (support* ADJ3 vector* ADJ3 machine*) OR ((recognition* OR detection* OR classification* OR predict* OR comput* OR diagnos*) ADJ3 (algorithm* OR network* OR computer-aided* OR automatic* OR automated*)) OR bayesian* OR radiomic* OR pattern-recognit* OR ((AI) ADJ (tool* OR model*))).ab,ti,kf. OR AI.ti.) **AND** (exp Bone Cysts/ OR exp Fibrous Dysplasia of Bone/ OR exp Lipoma/ OR exp Mesenchymoma/ OR exp Lymphoma/ OR exp Histiocytosis/ OR exp Histiocytosis, Sinus/ OR exp Sarcoma/ OR exp Soft Tissue Neoplasms/ OR exp Neuroma/ OR exp Lymphangioma/ OR exp Ganglion Cysts/ OR (GCTB OR DDLS OR GIST OR GISTs OR ((soft-tissue* OR adipos*-tissue* OR glomus* OR gastrointest-stroma* OR gastr*-intest*-stroma* OR spinal* OR rib OR skull OR sternal* OR tibial* OR sacrum* OR jaw OR maxillar* OR mandibular* OR odontogenic* OR connective-tissue* OR subcutan*-tissue* OR vein* OR muscle* OR musculoskeletal* OR bone* OR benign-notochordal-cell OR fibrous* OR osteoblast* OR osteoclast* OR synov* OR granular-cell* OR cartilag* OR joint* OR femoral* OR humerus* OR lympho* OR rhabdoid OR non-ossifying OR extramedullary-myeloid* OR atypical-lipmatous* OR nerve* OR giant-cell* OR schwann-cell* OR desmoplastic* OR myofibroblastic*) ADJ3 (tumor* OR tumour* OR cancer* OR neoplas* OR maligna* OR lesion* OR plasmacytom* OR metasta*)) OR ((vascular* OR arter* OR vessel* OR venal*) ADJ (tumor* OR tumour* OR cancer* OR neoplas* OR maligna* OR lesion* OR plasmacytom* OR metasta*)) OR ((lymph-node*) ADJ3 (tumor* OR tumour* OR cancer* OR neoplas* OR maligna* OR lesion* OR plasmacytom*)) OR adamantin* OR plasma-cell-granulom* OR glomangiom* OR myoma* OR desmoid* OR Bessel-Hagen OR diaphyseal-aclas* OR ((subungual OR multipl* OR dysplas* OR familial*) ADJ3 (exosto*)) OR osteocyst* OR ecchondrosis-ossificans OR chondrodysplasia OR adenosarcom* OR sarcom* OR gliosarcoma* OR adenosarcom* OR osteosarcom* OR chondrosarcom* OR chondrom* OR enchondrom* OR chondroblastom* OR chondromatosis* OR osteom* OR osteoblastom* OR osteochondrom* OR maffucci* OR hemangiom* OR haemangiom* OR hemangioendotheliom* OR angiosarcom* OR bone-cyst* OR osseous-cyst* OR intraosseous-gangli* OR intra-osseous-gangli* OR ganglion-cyst* OR jaw-cyst* OR subchondral-cyst* OR chordom* OR synoviom* OR ((fibro*) ADJ2 (dysplas* OR dystroph* OR osteodys*)) OR cherubism* OR osteofibrous-dysplasi* OR lipom* OR angiolipom* OR angiom* OR lipomatos* OR fetal-lipoma* OR Bannayan OR fatty-kidney OR fatty-pancreas* OR hibernom* OR mesenchym* OR adamantinom* OR hodgkin* OR erdheim-Chester* OR chester-erdheim* OR eosinophil*-granulom* OR histiocytos* OR dorfman-rosai-disease* OR nora-s-lesion* OR chondromesenchymal-hamartoma-of-chest-wall* OR lymphom* OR fibroma* OR osteoclastom* OR histioblastom* OR histiosarcom* OR leiomyosarcom* OR angioendotheliom* OR angioendotheliosarcom* OR hemangiosarcom* OR haemangiosarcom* OR haemangioendotheliom* OR hemangio-endotheliosarcom* OR hemangioendotheliom* OR hemangioendotheliosarcom* OR hemangio-endotheliom OR haemangio-endotheliom* OR lymphangiosarcom* OR Stewart-Treves OR rhabdomysarcom* OR myxofibrosarcom* OR myxosarcom* OR myofibrom* OR myofibroblastom* OR synoviom* OR myxom* OR myopericytom* OR fibrosarcom* OR fibroadenosarcom* OR dermatofibrosarcom* OR neurofibrosarcom* OR chloroma* OR extramedullary-leukaemia* OR extramedullary-leukemia* OR leukosarcom* OR liposarcom* OR neurom* OR perineurom* OR ganglionneurom* OR neurilemom* OR neurofibrom* OR neurothekeom* OR leiomyom* OR rhabdomyom* OR elastofibroma* OR lymphangiom* OR hemangiopericytom* OR haemangiopericytom* OR pericytom* OR myopericytom* OR glomangiopericytom* OR lipoblastom* OR schwannom* OR neurilemmom* OR neurinom* OR neurolemmom* OR neurilemom* OR neurolilemmon* OR ((pigment* OR arthritis*) ADJ3 (villonodular* OR villous*)) OR ((arthritis*) ADJ3 (pigment* OR schueller*)) OR ((synovitis*) ADJ3 (pigment* OR dendritic* OR villonodular*)) OR lymphosarcom* OR reticulosarcom* OR rhabdomyosarcom* OR ameloblastom* OR myosarcom* OR fibrosarcom* OR myoblastom* OR fibrous-histiocytom* OR histiomatos* OR reticulohistiocyt*).ab,ti,kf.) **AND** (exp Radiation Genomics/ OR Diagnostic Imaging/ OR exp Magnetic Resonance Imaging/ OR Laser-Doppler Flowmetry/ OR exp Ultrasonography/ OR (radiogenomic* OR ((radio OR radiat*) ADJ1 (genomic* OR diagnos*)) OR radiomic* OR ((diagnos* OR medical*) ADJ3 imag*) OR radiodiagnos* OR ((comput* OR positron) ADJ3 tomogra*) OR spect OR ct OR pet OR mri OR (magnetic ADJ3 resonance) OR ((nuclear OR mr OR multimodalit*) ADJ3 imaging*) OR rontgen OR roentgen OR ultraso* OR scintigra* OR (diffusion* ADJ3 (coefficient* OR weighted OR tensor)) OR dwi OR dti OR Doppler OR echogra*).ab,ti,kf.) NOT (news OR congres* OR abstract* OR book* OR chapter* OR dissertation abstract*).pt. NOT (Case Reports/ OR (case-report).ti.) NOT (exp animals/ NOT humans/)

**Cochrane**

((CNN OR (artificial* NEAR/3 intelligen*) OR ((machine OR deep) NEAR/3 learning) OR (neural* NEAR/3 network*) OR (classification* NEAR/3 (algorithm OR binary OR multiclass OR multilabel)) OR (classifier*) OR (data NEXT/1 mining*) OR (feature NEAR/3 detection*) OR (feature* NEAR/3 (extraction OR learning OR ranking OR selection OR analysis OR fusion*)) OR (k NEXT/1 nearest* NEAR/3 neighbo*) OR (kernel* NEAR/3 method*) OR (learning* NEAR/3 algorithm*) OR (least* NEAR/3 absolute* NEAR/3 shrinkage* NEAR/3 selection* NEAR/3 operator*) OR (Markov* NEAR/3 model*) OR (memristor*) OR (network* NEAR/3 learning*) OR (perceptron*) OR (radial* NEAR/3 basis* NEAR/3 function*) OR (random* NEAR/3 forest*) OR (recursive* NEAR/3 feature* NEAR/3 elimination*) OR (recursive* NEAR/3 partitioning*) OR (support* NEAR/3 vector* NEAR/3 machine*) OR ((recognition* OR detection* OR classification* OR predict* OR comput* OR diagnos*) NEAR/3 (algorithm* OR network* OR computer NEXT/1 aided* OR automatic* OR automated*)) OR bayesian* OR radiomic* OR pattern NEXT/1 recognit* OR ((AI) NEXT/1 (tool* OR model*))):ab,ti,kw OR AI:ti) **AND** ((GCTB OR DDLS OR GIST OR GISTs OR ((soft NEXT/1 tissue* OR adipos* NEXT/1 tissue* OR glomus* OR gastrointest NEXT/1 stroma* OR gastr* NEXT/1 intest* NEXT/1 stroma* OR spinal* OR rib OR skull OR sternal* OR tibial* OR sacrum* OR jaw OR maxillar* OR mandibular* OR odontogenic* OR connective NEXT/1 tissue* OR subcutan* NEXT/1 tissue* OR vein* OR muscle* OR musculoskeletal* OR bone* OR benign NEXT/1 notochordal NEXT/1 cell OR fibrous* OR osteoblast* OR osteoclast* OR synov* OR granular NEXT/1 cell* OR cartilag* OR joint* OR femoral* OR humerus* OR lympho* OR rhabdoid OR non NEXT/1 ossifying OR extramedullary NEXT/1 myeloid* OR atypical NEXT/1 lipmatous* OR nerve* OR giant NEXT/1 cell* OR schwann NEXT/1 cell* OR desmoplastic* OR myofibroblastic*) NEAR/3 (tumor* OR tumour* OR cancer* OR neoplas* OR maligna* OR lesion* OR plasmacytom* OR metasta*)) OR ((vascular* OR arter* OR vessel* OR venal*) NEXT/1 (tumor* OR tumour* OR cancer* OR neoplas* OR maligna* OR lesion* OR plasmacytom* OR metasta*)) OR ((lymph NEXT/1 node*) NEAR/3 (tumor* OR tumour* OR cancer* OR neoplas* OR maligna* OR lesion* OR plasmacytom*)) OR adamantin* OR plasma NEXT/1 cell NEXT/1 granulom* OR glomangiom* OR myoma* OR desmoid* OR Bessel NEXT/1 Hagen OR diaphyseal NEXT/1 aclas* OR ((subungual OR multipl* OR dysplas* OR familial*) NEAR/3 (exosto*)) OR osteocyst* OR ecchondrosis NEXT/1 ossificans OR chondrodysplasia OR adenosarcom* OR sarcom* OR gliosarcoma* OR adenosarcom* OR osteosarcom* OR chondrosarcom* OR chondrom* OR enchondrom* OR chondroblastom* OR chondromatosis* OR osteom* OR osteoblastom* OR osteochondrom* OR maffucci* OR hemangiom* OR haemangiom* OR hemangioendotheliom* OR angiosarcom* OR bone NEXT/1 cyst* OR osseous NEXT/1 cyst* OR intraosseous NEXT/1 gangli* OR intra NEXT/1 osseous NEXT/1 gangli* OR ganglion NEXT/1 cyst* OR jaw NEXT/1 cyst* OR subchondral NEXT/1 cyst* OR chordom* OR synoviom* OR ((fibro*) NEAR/2 (dysplas* OR dystroph* OR osteodys*)) OR cherubism* OR osteofibrous NEXT/1 dysplasi* OR lipom* OR angiolipom* OR angiom* OR lipomatos* OR fetal NEXT/1 lipoma* OR Bannayan OR fatty NEXT/1 kidney OR fatty NEXT/1 pancreas* OR hibernom* OR mesenchym* OR adamantinom* OR hodgkin* OR erdheim NEXT/1 Chester* OR chester NEXT/1 erdheim* OR eosinophil* NEXT/1 granulom* OR histiocytos* OR dorfman NEXT/1 rosai NEXT/1 disease* OR nora NEXT/1 s NEXT/1 lesion* OR chondromesenchymal NEXT/1 hamartoma NEXT/1 of NEXT/1 chest NEXT/1 wall* OR lymphom* OR fibroma* OR osteoclastom* OR histioblastom* OR histiosarcom* OR leiomyosarcom* OR angioendotheliom* OR angioendotheliosarcom* OR hemangiosarcom* OR haemangiosarcom* OR haemangioendotheliom* OR hemangio NEXT/1 endotheliosarcom* OR hemangioendotheliom* OR hemangioendotheliosarcom* OR hemangio NEXT/1 endotheliom OR haemangio NEXT/1 endotheliom* OR lymphangiosarcom* OR Stewart NEXT/1 Treves OR rhabdomysarcom* OR myxofibrosarcom* OR myxosarcom* OR myofibrom* OR myofibroblastom* OR synoviom* OR myxom* OR myopericytom* OR fibrosarcom* OR fibroadenosarcom* OR dermatofibrosarcom* OR neurofibrosarcom* OR chloroma* OR extramedullary NEXT/1 leukaemia* OR extramedullary NEXT/1 leukemia* OR leukosarcom* OR liposarcom* OR neurom* OR perineurom* OR ganglionneurom* OR neurilemom* OR neurofibrom* OR neurothekeom* OR leiomyom* OR rhabdomyom* OR elastofibroma* OR lymphangiom* OR hemangiopericytom* OR haemangiopericytom* OR pericytom* OR myopericytom* OR glomangiopericytom* OR lipoblastom* OR schwannom* OR neurilemmom* OR neurinom* OR neurolemmom* OR neurilemom* OR neurolilemmon* OR ((pigment* OR arthritis*) NEAR/3 (villonodular* OR villous*)) OR ((arthritis*) NEAR/3 (pigment* OR schueller*)) OR ((synovitis*) NEAR/3 (pigment* OR dendritic* OR villonodular*)) OR lymphosarcom* OR reticulosarcom* OR rhabdomyosarcom* OR ameloblastom* OR myosarcom* OR fibrosarcom* OR myoblastom* OR fibrous NEXT/1 histiocytom* OR histiomatos* OR reticulohistiocyt*):ab,ti,kw) **AND** ((radiogenomic* OR ((radio OR radiat*) NEXT/1 (genomic* OR diagnos*)) OR radiomic* OR ((diagnos* OR medical*) NEAR/3 imag*) OR radio NEXT/1 genomic* OR radiomic* OR (diagnos* NEAR/3 imag*) OR radiodiagnos* OR ((comput* OR positron) NEAR/3 tomogra*) OR spect OR ct OR pet OR mri OR (magnetic NEAR/3 resonance) OR ((nuclear OR mr OR multimodalit*) NEAR/3 imaging*) OR rontgen OR roentgen OR ultraso* OR scintigra* OR (diffusion* NEAR/3 (coefficient* OR weighted OR tensor)) OR dwi OR dti OR Doppler OR echogra*):ab,ti,kw) NOT "conference abstract":pt

**Web of Science**

TS=(((CNN OR (artificial* NEAR/2 intelligen*) OR ((machine OR deep) NEAR/2 learning) OR (neural* NEAR/2 network*) OR (classification* NEAR/2 (algorithm OR binary OR multiclass OR multilabel)) OR (classifier*) OR (data-mining*) OR (feature NEAR/2 detection*) OR (feature* NEAR/2 (extraction OR learning OR ranking OR selection OR analysis OR fusion*)) OR (k-nearest* NEAR/2 neighbo*) OR (kernel* NEAR/2 method*) OR (learning* NEAR/2 algorithm*) OR (least* NEAR/2 absolute* NEAR/2 shrinkage* NEAR/2 selection* NEAR/2 operator*) OR (Markov* NEAR/2 model*) OR (memristor*) OR (network* NEAR/2 learning*) OR (perceptron*) OR (radial* NEAR/2 basis* NEAR/2 function*) OR (random* NEAR/2 forest*) OR (recursive* NEAR/2 feature* NEAR/2 elimination*) OR (recursive* NEAR/2 partitioning*) OR (support* NEAR/2 vector* NEAR/2 machine*) OR ((recognition* OR detection* OR classification* OR predict* OR comput* OR diagnos*) NEAR/2 (algorithm* OR network* OR computer-aided* OR automatic* OR automated*)) OR bayesian* OR radiomic* OR pattern-recognit* OR ((AI) NEAR/1 (tool* OR model*))) OR AI:ti) AND ((GCTB OR DDLS OR GIST OR GISTs OR ((soft-tissue* OR adipos*-tissue* OR glomus* OR gastrointest-stroma* OR gastr*-intest*-stroma* OR spinal* OR rib OR skull OR sternal* OR tibial* OR sacrum* OR jaw OR maxillar* OR mandibular* OR odontogenic* OR connective-tissue* OR subcutan*-tissue* OR vein* OR muscle* OR musculoskeletal* OR bone* OR benign-notochordal-cell OR fibrous* OR osteoblast* OR osteoclast* OR synov* OR granular-cell* OR cartilag* OR joint* OR femoral* OR humerus* OR lympho* OR rhabdoid OR non-ossifying OR extramedullary-myeloid* OR atypical-lipmatous* OR nerve* OR giant-cell* OR schwann-cell* OR desmoplastic* OR myofibroblastic*) NEAR/2 (tumor* OR tumour* OR cancer* OR neoplas* OR maligna* OR lesion* OR plasmacytom* OR metasta*)) OR ((vascular* OR arter* OR vessel* OR venal*) NEAR/1 (tumor* OR tumour* OR cancer* OR neoplas* OR maligna* OR lesion* OR plasmacytom* OR metasta*)) OR ((lymph-node*) NEAR/2 (tumor* OR tumour* OR cancer* OR neoplas* OR maligna* OR lesion* OR plasmacytom*)) OR adamantin* OR plasma-cell-granulom* OR glomangiom* OR myoma* OR desmoid* OR Bessel-Hagen OR diaphyseal-aclas* OR ((subungual OR multipl* OR dysplas* OR familial*) NEAR/2 (exosto*)) OR osteocyst* OR ecchondrosis-ossificans OR chondrodysplasia OR adenosarcom* OR sarcom* OR gliosarcoma* OR adenosarcom* OR osteosarcom* OR chondrosarcom* OR chondrom* OR enchondrom* OR chondroblastom* OR chondromatosis* OR osteom* OR osteoblastom* OR osteochondrom* OR maffucci* OR hemangiom* OR haemangiom* OR hemangioendotheliom* OR angiosarcom* OR bone-cyst* OR osseous-cyst* OR intraosseous-gangli* OR intra-osseous-gangli* OR ganglion-cyst* OR jaw-cyst* OR subchondral-cyst* OR chordom* OR synoviom* OR ((fibro*) NEAR/2 (dysplas* OR dystroph* OR osteodys*)) OR cherubism* OR osteofibrous-dysplasi* OR lipom* OR angiolipom* OR angiom* OR lipomatos* OR fetal-lipoma* OR Bannayan OR fatty-kidney OR fatty-pancreas* OR hibernom* OR mesenchym* OR adamantinom* OR hodgkin* OR erdheim-Chester* OR chester-erdheim* OR eosinophil*-granulom* OR histiocytos* OR dorfman-rosai-disease* OR nora-s-lesion* OR chondromesenchymal-hamartoma-of-chest-wall* OR lymphom* OR fibroma* OR osteoclastom* OR histioblastom* OR histiosarcom* OR leiomyosarcom* OR angioendotheliom* OR angioendotheliosarcom* OR hemangiosarcom* OR haemangiosarcom* OR haemangioendotheliom* OR hemangio-endotheliosarcom* OR hemangioendotheliom* OR hemangioendotheliosarcom* OR hemangio-endotheliom OR haemangio-endotheliom* OR lymphangiosarcom* OR Stewart-Treves OR rhabdomysarcom* OR myxofibrosarcom* OR myxosarcom* OR myofibrom* OR myofibroblastom* OR synoviom* OR myxom* OR myopericytom* OR fibrosarcom* OR fibroadenosarcom* OR dermatofibrosarcom* OR neurofibrosarcom* OR chloroma* OR extramedullary-leukaemia* OR extramedullary-leukemia* OR leukosarcom* OR liposarcom* OR neurom* OR perineurom* OR ganglionneurom* OR neurilemom* OR neurofibrom* OR neurothekeom* OR leiomyom* OR rhabdomyom* OR elastofibroma* OR lymphangiom* OR hemangiopericytom* OR haemangiopericytom* OR pericytom* OR myopericytom* OR glomangiopericytom* OR lipoblastom* OR schwannom* OR neurilemmom* OR neurinom* OR neurolemmom* OR neurilemom* OR neurolilemmon* OR ((pigment* OR arthritis*) NEAR/2 (villonodular* OR villous*)) OR ((arthritis*) NEAR/2 (pigment* OR schueller*)) OR ((synovitis*) NEAR/2 (pigment* OR dendritic* OR villonodular*)) OR lymphosarcom* OR reticulosarcom* OR rhabdomyosarcom* OR ameloblastom* OR myosarcom* OR fibrosarcom* OR myoblastom* OR fibrous-histiocytom* OR histiomatos* OR reticulohistiocyt*)) AND ((radiogenomic* OR ((radio OR radiat*) NEAR/1 (genomic* OR diagnos*)) OR radiomic* OR ((diagnos* OR medical*) NEAR/2 imag*) OR radio-genomic* OR radiomic* OR (diagnos* NEAR/2 imag*) OR radiodiagnos* OR ((comput* OR positron) NEAR/2 tomogra*) OR spect OR ct OR pet OR mri OR (magnetic NEAR/2 resonance) OR ((nuclear OR mr OR multimodalit*) NEAR/2 imaging*) OR rontgen OR roentgen OR ultraso* OR scintigra* OR (diffusion* NEAR/2 (coefficient* OR weighted OR tensor)) OR dwi OR dti OR Doppler OR echogra*)) NOT ((animal* OR rat OR rats OR mouse OR mice OR murine OR dog OR dogs OR canine OR cat OR cats OR feline OR rabbit OR cow OR cows OR bovine OR rodent* OR sheep OR ovine OR pig OR swine OR porcine OR veterinar* OR chick* OR zebrafish* OR baboon* OR nonhuman* OR primate* OR cattle* OR goose OR geese OR duck OR macaque* OR avian* OR bird* OR fish*) NOT (human* OR patient* OR women OR woman OR men OR man))) NOT DT=(Meeting Abstract OR Meeting Summary) NOT TI=(case-report)

**Google Scholar**

“artificial intelligence”|”machine|deep learning”|”neural network”|radiomics “musculoskeletal|bone|nerve tumor|tumour|neoplasm|cancer”|“soft tissue tumor|tumour|neoplasm|cancer” radiomics|radiogenomics|”diagnostic imaging”|”radio diagnosis”|MRI|doppler
