# Supplementary material for: AI in radiological imaging of soft-tissue and bone tumours: a systematic review evaluating against CLAIM and FUTURE-AI guidelines
Source: eBioMedicine. 2025 Mar 20;114:105642. doi: 10.1016/j.ebiom.2025.105642 (PMC11976239; doi:10.1016/j.ebiom.2025.105642)
Supplement: Appendix 3 [file mmc3.docx]

1. Alabdulkreem E, Saeed MK, Alotaibi SS, Allafi R, Mohamed A, Hamza MA. Bone Cancer Detection and Classification Using Owl Search Algorithm With Deep Learning on X-Ray Images. *Ieee Access* 2023; **11**: 109095-103.

2. Alaoui EA, Tekouabou SCK, Hartini S, Rustam Z, Silkan H, Agoujil S. Improvement in Automated Diagnosis of Soft Tissues Tumors Using Machine Learning. *Big Data Min Anal* 2021; **4**(1): 33-46.

3. Alge O, Lu L, Li Z, Hua Y, Gryak J, Najarian K. Automated Classification of Osteosarcoma and Benign Tumors using RNA-seq and Plain X-ray. *Annu Int Conf IEEE Eng Med Biol Soc* 2020; **2020**: 1165-8.

4. Altameem T. Fuzzy rank correlation-based segmentation method and deep neural network for bone cancer identification. *Neural Computing and Applications* 2020.

5. Amini B, Chenglei L, Duran-Sierra E, Wang WL, Canjirathinkal MA, Moradi H, et al. Role of Apparent Diffusion Coefficient Map-Based First- and High-Order Radiomic Features for the Discrimination of Sacral Chordomas and Chondrosarcomas With Overlapping Conventional Imaging Features. *JCO precis oncol* 2023; **7**: e2300243.

6. Ao W, Cheng G, Lin B, Yang R, Liu X, Zhou C, et al. A novel CT-based radiomic nomogram for predicting the recurrence and metastasis of gastric stromal tumors. *Am J Cancer Res* 2021; **11**(6): 3123-34.

7. Arthur A, Orton MR, Emsley R, Vit S, Kelly-Morland C, Strauss D, et al. A CT-based radiomics classification model for the prediction of histological type and tumour grade in retroperitoneal sarcoma (RADSARC-R): a retrospective multicohort analysis. *Lancet Oncol* 2023; **24**(11): 1277-86.

8. Ba-Ssalamah A, Muin D, Schernthaner R, Kulinna-Cosentini C, Bastati N, Stift J, et al. Texture-based classification of different gastric tumors at contrast-enhanced CT. *Eur J Radiol* 2013; **82**(10): e537-43.

9. Bandyopadhyay O, Biswas A, Bhattacharya BB. Bone-Cancer Assessment and Destruction Pattern Analysis in Long-Bone X-ray Image. *J Digit Imaging* 2019; **32**(2): 300-13.

10. Banerjee I, Crawley A, Bhethanabotla M, Daldrup-Link HE, Rubin DL. Transfer learning on fused multiparametric MR images for classifying histopathological subtypes of rhabdomyosarcoma. *Comput Med Imaging Graph* 2018; **65**: 167-75.

11. Baskaran K, Malathi R, Thirusakthimurugan P. Feature Fusion for FDG-PET and MRI for Automated Extra Skeletal Bone Sarcoma Classification. *Mater Today-Proc* 2018; **5**(1): 1879-89.

12. Blackledge MD, Winfield JM, Miah A, Strauss D, Thway K, Morgan VA, et al. Supervised Machine-Learning Enables Segmentation and Evaluation of Heterogeneous Post-treatment Changes in Multi-Parametric MRI of Soft-Tissue Sarcoma. *Front oncol* 2019; **9**: 941.

13. Bouhamama A, Leporq B, Khaled W, Nemeth A, Brahmi M, Dufau J, et al. Prediction of Histologic Neoadjuvant Chemotherapy Response in Osteosarcoma Using Pretherapeutic MRI Radiomics. *Radiol Imaging Cancer* 2022; **4**(5): e210107.

14. Breden S, Hinterwimmer F, Consalvo S, Neumann J, Knebel C, von Eisenhart-Rothe R, et al. Deep Learning-Based Detection of Bone Tumors around the Knee in X-rays of Children. *J Clin Med* 2023; **12**(18).

15. Cao C, Yi Z, Xie M, Xie Y, Tang X, Tu B, et al. Machine learning-based radiomics analysis for predicting local recurrence of primary dermatofibrosarcoma protuberans after surgical treatment. *Radiother Oncol* 2023; **186**: 109737.

16. Cao Y, Wang Z, Ren J, Liu W, Da H, Yang X, Bao H. Differentiation of retroperitoneal paragangliomas and schwannomas based on computed tomography radiomics. *Sci rep* 2023; **13**(1): 9253.

17. Cappello G, Giannini V, Cannella R, Tabone E, Ambrosini I, Molea F, et al. A mutation-based radiomics signature predicts response to imatinib in Gastrointestinal Stromal Tumors (GIST). *Eur J Radiol Open* 2023; **11**: 100505.

18. Casale R, De Angelis R, Coquelet N, Mokhtari A, Bali MA. The Impact of Edema on MRI Radiomics for the Prediction of Lung Metastasis in Soft Tissue Sarcoma. *Diagnostics* 2023; **13(19)**.

19. Casale R, Varriano G, Santone A, Messina C, Casale C, Gitto S, et al. Predicting risk of metastases and recurrence in soft-tissue sarcomas via Radiomics and Formal Methods. *Jamia Open* 2023; **6**(2): ooad025.

20. Cay N, Mendi BAR, Batur H, Erdogan F. Discrimination of lipoma from atypical lipomatous tumor/well-differentiated liposarcoma using magnetic resonance imaging radiomics combined with machine learning. *Jpn J Radiol* 2022; **40**(9): 951-60.

21. Chen CY, Chiou HJ, Chou SY, Chiou SY, Wang HK, Chou YH, Chiang HK. Computer-aided diagnosis of soft-tissue tumors using sonographic morphologic and texture features. *Acad Radiol* 2009; **16**(12): 1531-8.

22. Chen G, Fan L, Liu J, Wu S. Machine learning-based predictive model for the differential diagnosis of <= 5 cm gastric stromal tumor and gastric schwannoma based on CT images. *Discov Oncol* 2023; **14**(1): 186.

23. Chen H, Liu J, Cheng Z, Lu X, Wang X, Lu M, et al. Development and external validation of an MRI-based radiomics nomogram for pretreatment prediction for early relapse in osteosarcoma: A retrospective multicenter study. *Eur J Radiol* 2020; **129**: 109066.

24. Chen H, Zhang X, Wang X, Quan X, Deng Y, Lu M, et al. MRI-based radiomics signature for pretreatment prediction of pathological response to neoadjuvant chemotherapy in osteosarcoma: a multicenter study. *Eur Radiol* 2021; **31**(10): 7913-24.

25. Chen S, Li N, Tang Y, Chen B, Fang H, Qi S, et al. Radiomics Analysis of Fat Saturated T2-Weighted MRI Sequences for Prognostic Prediction to Soft-Tissue Sarcoma of the Extremities and Trunk Treated With Neoadjuvant Radiotherapy. *Int J Radiat Oncol Biol Phys* 2021; **111**(3): e315.

26. Chen T, Liu S, Li Y, Feng X, Xiong W, Zhao X, et al. Developed and validated a prognostic nomogram for recurrence-free survival after complete surgical resection of local primary gastrointestinal stromal tumors based on deep learning. *EBioMedicine* 2019; **39**: 272-9.

27. Chen T, Ning Z, Xu L, Feng X, Han S, Roth HR, et al. Radiomics nomogram for predicting the malignant potential of gastrointestinal stromal tumours preoperatively. *Eur Radiol* 2019; **29**(3): 1074-82.

28. Chen W, Ayoub M, Liao M, Shi R, Zhang M, Su F, et al. A fusion of VGG-16 and ViT models for improving bone tumor classification in computed tomography. *J Bone Oncol* 2023; **43**: 100508.

29. Chen X, Huang Y, He L, Zhang T, Zhang L, Ding H. CT-Based Radiomics to Differentiate Pelvic Rhabdomyosarcoma From Yolk Sac Tumors in Children. *Front oncol* 2020; **10**: 584272.

30. Chen Z, Xu L, Zhang C, Huang C, Wang M, Feng Z, Xiong Y. CT Radiomics Model for Discriminating the Risk Stratification of Gastrointestinal Stromal Tumors: A Multi-Class Classification and Multi-Center Study. *Front oncol* 2021; **11**: 654114.

31. Cheng Y, Yang L, Wang Y, Kuang L, Pan X, Chen L, et al. Development and validation of a radiomics model based on T2-weighted imaging for predicting the efficacy of high intensity focused ultrasound ablation in uterine fibroids. *Quant Imaging Med Surg* 2024; **14**(2): 1803-19.

32. Chianca V, Cuocolo R, Gitto S, Albano D, Merli I, Badalyan J, et al. Radiomic Machine Learning Classifiers in Spine Bone Tumors: A Multi-Software, Multi-Scanner Study. *Eur J Radiol* 2021; **137**: 109586.

33. Chiappa V, Interlenghi M, Salvatore C, Bertolina F, Bogani G, Ditto A, et al. Using rADioMIcs and machine learning with ultrasonography for the differential diagnosis of myometRiAL tumors (the ADMIRAL pilot study). Radiomics and differential diagnosis of myometrial tumors. *Gynecol Oncol* 2021; **161**(3): 838-44.

34. Chiou HJ, Chen CY, Liu TC, Chiou SY, Wang HK, Chou YH, Chiang HK. Computer-aided diagnosis of peripheral soft tissue masses based on ultrasound imaging. *Comput Med Imaging Graph* 2009; **33**(5): 408-13.

35. Chu H, Pang P, He J, Zhang D, Zhang M, Qiu Y, et al. Value of radiomics model based on enhanced computed tomography in risk grade prediction of gastrointestinal stromal tumors. *Sci rep* 2021; **11**(1): 12009.

36. Cilengir AH, Evrimler S, Serel TA, Uluc E, Tosun O. The diagnostic value of magnetic resonance imaging-based texture analysis in differentiating enchondroma and chondrosarcoma. *Skeletal Radiol* 2023; **52**(5): 1039-49.

37. Consalvo S, Hinterwimmer F, Neumann J, Steinborn M, Salzmann M, Seidl F, et al. Two-Phase Deep Learning Algorithm for Detection and Differentiation of Ewing Sarcoma and Acute Osteomyelitis in Paediatric Radiographs. *Anticancer Res* 2022; **42**(9): 4371-80.

38. Corino VDA, Montin E, Messina A, Casali PG, Gronchi A, Marchiano A, Mainardi LT. Radiomic analysis of soft tissues sarcomas can distinguish intermediate from high-grade lesions. *J Magn Reson Imaging* 2018; **47**(3): 829-40.

39. Crombe A, Fadli D, Buy X, Italiano A, Saut O, Kind M. High-Grade Soft-Tissue Sarcomas: Can Optimizing Dynamic Contrast-Enhanced MRI Postprocessing Improve Prognostic Radiomics Models? *J Magn Reson Imaging* 2020; **52**(1): 282-97.

40. Crombe A, Lucchesi C, Bertolo F, Kind M, Spalato-Ceruso M, Toulmonde M, et al. Integration of pre-treatment computational radiomics, deep radiomics, and transcriptomics enhances soft-tissue sarcoma patient prognosis. *NPJ Precis Oncol* 2024; **8**(1): 129.

41. Crombe A, Perier C, Kind M, De Senneville BD, Le Loarer F, Italiano A, et al. T2 -based MRI Delta-radiomics improve response prediction in soft-tissue sarcomas treated by neoadjuvant chemotherapy. *J Magn Reson Imaging* 2019; **50**(2): 497-510.

42. Dai M, Liu Y, Hu Y, Li G, Zhang J, Xiao Z, Lv F. Combining multiparametric MRI features-based transfer learning and clinical parameters: application of machine learning for the differentiation of uterine sarcomas from atypical leiomyomas. *Eur Radiol* 2022; **32**(11): 7988-97.

43. Dai Y, Yin P, Mao N, Sun C, Wu J, Cheng G, Hong N. Differentiation of Pelvic Osteosarcoma and Ewing Sarcoma Using Radiomic Analysis Based on T2-Weighted Images and Contrast-Enhanced T1-Weighted Images. *Biomed Res Int* 2020; **2020**: 9078603.

44. Deng J, Zeng W, Shi Y, Kong W, Guo S. Fusion of FDG-PET Image and Clinical Features for Prediction of Lung Metastasis in Soft Tissue Sarcomas. *Comput math methods med* 2020; **2020**: 8153295.

45. Deng KH, Wang B, Ma SS, Xue Z, Cao XH. A Bone Lesion Identification Network (BLIN) in CT Images with Weakly Supervised Learning. 2024; **14349**: 243-52.

46. Ding Y, Wang Z, Xu P, Ma Y, Yao W, Li K, Gong Y. MRI-based radiomics in distinguishing Kaposiform hemangioendothelioma (KHE) and fibro-adipose vascular anomaly (FAVA) in extremities: A preliminary retrospective study. *J Pediatr Surg* 2022; **57**(7): 1228-34.

47. Djuricic GJ, Ahammer H, Rajkovic S, Kovac JD, Milosevic Z, Sopta JP, Radulovic M. Directionally Sensitive Fractal Radiomics Compatible With Irregularly Shaped Magnetic Resonance Tumor Regions of Interest: Association With Osteosarcoma Chemoresistance. *J Magn Reson Imaging* 2023; **57**(1): 248-58.

48. Do BH, Langlotz C, Beaulieu CF. Bone Tumor Diagnosis Using a Naïve Bayesian Model of Demographic and Radiographic Features. *J Digit Imaging* 2017; **30**(5): 640-7.

49. Do NT, Jung ST, Yang HJ, Kim SH. Multi-level seg-unet model with global and patch-based x-ray images for knee bone tumor detection. *Diagn* 2021; **11**(4).

50. Dong J, Yu M, Miao Y, Shen H, Sui Y, Liu Y, et al. Differential Diagnosis of Solitary Fibrous Tumor/Hemangiopericytoma and Angiomatous Meningioma Using Three-Dimensional Magnetic Resonance Imaging Texture Feature Model. *Biomed Res Int* 2020; **2020**: 5042356.

51. Dong Z, Zhao X, Zheng H, Zheng H, Chen D, Cao J, et al. Efficacy of real-time artificial intelligence-aid endoscopic ultrasonography diagnostic system in discriminating gastrointestinal stromal tumors and leiomyomas: a multicenter diagnostic study. *EClinicalMedicine* 2024; **73**: 102656.

52. Dou Y, Li X, Tao J, Dong Y, Xu N, Wang S. Prediction of high-grade soft-tissue sarcoma using a combined intratumoural and peritumoural MRI-based radiomics nomogram. *Clin Radiol* 2023; **78**(12): e1032-e40.

53. Erdem F, Tamsel I, Demirpolat G. The use of radiomics and machine learning for the differentiation of chondrosarcoma from enchondroma. *J Clin Ultrasound* 2023; **51**(6): 1027-35.

54. Escobars T, Vauclin S, Orlhac F, Nioche C, Pineau P, Buvat I. An original voxel-wise supervised analysis of tumors with multimodal radiomics to highlight predictive biologicalpatterns. *J Nucl Med* 2021; **62**(SUPPL 1).

55. Eweje FR, Bao B, Wu J, Dalal D, Liao WH, He Y, et al. Deep Learning for Classification of Bone Lesions on Routine MRI. *EBioMedicine* 2021; **68**: 103402.

56. Fadli D, Kind M, Michot A, Le Loarer F, Crombe A. Natural Changes in Radiological and Radiomics Features on MRIs of Soft-Tissue Sarcomas Naive of Treatment: Correlations With Histology and Patients' Outcomes. *J Magn Reson Imaging* 2022; **56**(1): 77-96.

57. Fan L, Gong X, Zheng C, Li J. Data pyramid structure for optimizing EUS-based GISTs diagnosis in multi-center analysis with missing label. *Comput Biol Med* 2024; **169**.

58. Fares R, Atlan LD, Druckmann I, Factor S, Gortzak Y, Segal O, et al. Imaging-Based Deep Learning for Predicting Desmoid Tumor Progression. *J Imaging* 2024; **10**(5).

59. Farhidzadeh H, Goldgof DB, Hall LO, Scott JG, Gatenby RA, Gillies RJ, Raghavan M. A Quantitative Histogram-based Approach to Predict Treatment Outcome for Soft Tissue Sarcomas Using Pre- and Post-treatment MRIs. 2016: 4549-54.

60. Feng N, Chen HY, Wang XJ, Lu YF, Zhou JP, Zhou QM, et al. A CT-based nomogram established for differentiating gastrointestinal heterotopic pancreas from gastrointestinal stromal tumor: compared with a machine-learning model. *BMC med imaging* 2023; **23**(1): 131.

61. Feng Q, Tang B, Zhang Y, Liu X. Prediction of the Ki-67 expression level and prognosis of gastrointestinal stromal tumors based on CT radiomics nomogram. *Int j comput assist radiol surg* 2022; **17**(6): 1167-75.

62. Fichera G, Giraudo C, Stramare R, Bisogno G, Motta R, Evangelista L, et al. Radiomic features as biomarkers of soft tissue paediatric sarcomas: results of a PET/MR study. *Insights Imaging* 2022; **14**: 112.

63. Fields BKK, Demirjian NL, Cen SY, Varghese BA, Hwang DH, Lei X, et al. Predicting Soft Tissue Sarcoma Response to Neoadjuvant Chemotherapy Using an MRI-Based Delta-Radiomics Approach. *Mol Imaging Biol* 2023; **25**(4): 776-87.

64. Fields BKK, Demirjian NL, Hwang DH, Varghese BA, Cen SY, Lei X, et al. Whole-tumor 3D volumetric MRI-based radiomics approach for distinguishing between benign and malignant soft tissue tumors. *Eur Radiol* 2021; **31**(11): 8522-35.

65. Findlay MC, Yost S, Bauer SZ, Cole KL, Henson JC, Lucke-Wold B, et al. Application of Radiomics to the Differential Diagnosis of Temporal Bone Skull Base Lesions: A Pilot Study. *World Neurosurg* 2023; **172**: e540-e54.

66. Foreman SC, Llorián-Salvador O, David DE, Rösner VKN, Rischewski JF, Feuerriegel GC, et al. Development and Evaluation of MR-Based Radiogenomic Models to Differentiate Atypical Lipomatous Tumors from Lipomas. *Cancers (Basel)* 2023; **15**(7).

67. Fradet G, Ayde R, Bottois H, El Harchaoui M, Khaled W, Drape JL, et al. Prediction of lipomatous soft tissue malignancy on MRI: comparison between machine learning applied to radiomics and deep learning. *Eur Radiol Exp* 2022; **6**(1): 41.

68. Gao Y, Ghodrati V, Kalbasi A, Fu J, Ruan D, Cao M, et al. Prediction of soft tissue sarcoma response to radiotherapy using longitudinal diffusion MRI and a deep neural network with generative adversarial network-based data augmentation. *Med Phys* 2021; **48**(6): 3262-372.

69. Gao Y, Kalbasi A, Hsu W, Ruan D, Fu J, Shao J, et al. Treatment effect prediction for sarcoma patients treated with preoperative radiotherapy using radiomics features from longitudinal diffusion-weighted MRIs. *Phys Med Biol* 2020; **65**(17): 175006.

70. Gawade S, Bhansali A, Patil K, Shaikh D. Application of the convolutional neural networks and supervised deep-learning methods for osteosarcoma bone cancer detection. *Healthc Anal* 2023; **3**.

71. George A, Ayshwarya B. Adaptive FLAME based segmentation and classification for bone cancer detection. *… Conference on Artificial Intelligence …* 2023.

72. Georgeanu V, Mamuleanu ML. Convolutional neural networks for automated detection and classification of bone tumors in magnetic resonance imaging. *… on Artificial Intelligence …* 2021.

73. Georgeanu VA, Mămuleanu M, Ghiea S, Selișteanu D. Malignant Bone Tumors Diagnosis Using Magnetic Resonance Imaging Based on Deep Learning Algorithms. *Medicina (Kaunas)* 2022; **58**(5).

74. Gitto S, Annovazzi A, Nulle K, Interlenghi M, Salvatore C, Anelli V, et al. X-rays radiomics-based machine learning classification of atypical cartilaginous tumour and high-grade chondrosarcoma of long bones. *EBioMedicine* 2024; **101**: 105018.

75. Gitto S, Bologna M, Corino VDA, Emili I, Albano D, Messina C, et al. Diffusion-weighted MRI radiomics of spine bone tumors: feature stability and machine learning-based classification performance. *Radiol Med (Torino)* 2022; **127**(5): 518-25.

76. Gitto S, Corino VDA, Annovazzi A, Milazzo Machado E, Bologna M, Marzorati L, et al. 3D vs. 2D MRI radiomics in skeletal Ewing sarcoma: Feature reproducibility and preliminary machine learning analysis on neoadjuvant chemotherapy response prediction. *Front oncol* 2022; **12**: 1016123.

77. Gitto S, Cuocolo R, Albano D, Chianca V, Messina C, Gambino A, et al. MRI radiomics-based machine-learning classification of bone chondrosarcoma. *Eur J Radiol* 2020; **128**: 109043.

78. Gitto S, Cuocolo R, Annovazzi A, Anelli V, Acquasanta M, Cincotta A, et al. CT radiomics-based machine learning classification of atypical cartilaginous tumours and appendicular chondrosarcomas. *EBioMedicine* 2021; **68**: 103407.

79. Gitto S, Cuocolo R, van Langevelde K, van de Sande MAJ, Parafioriti A, Luzzati A, et al. MRI radiomics-based machine learning classification of atypical cartilaginous tumour and grade II chondrosarcoma of long bones. *EBioMedicine* 2022; **75**: 103757.

80. Gitto S, Interlenghi M, Cuocolo R, Salvatore C, Giannetta V, Badalyan J, et al. MRI radiomics-based machine learning for classification of deep-seated lipoma and atypical lipomatous tumor of the extremities. *Radiol Med (Torino)* 2023; **128**(8): 989-98.

81. Gruber L, Gruber H, Luger AK, Glodny B, Henninger B, Loizides A. Diagnostic hierarchy of radiological features in soft tissue tumours and proposition of a simple diagnostic algorithm to estimate malignant potential of an unknown mass. *Eur J Radiol* 2017; **95**: 102-10.

82. Grueneisen J, Schaarschmidt B, Demircioglu A, Chodyla M, Martin O, Bertram S, et al. (18)F-FDG PET/MRI for Therapy Response Assessment of Isolated Limb Perfusion in Patients with Soft-Tissue Sarcomas. *J Nucl Med* 2019; **60**(11): 1537-42.

83. Guo C, Zhou H, Chen X, Feng Z. Computed tomography texture-based models for predicting KIT exon 11 mutation of gastrointestinal stromal tumors. *Heliyon* 2023; **9**(10): e20983.

84. Guo J, Li YM, Guo H, Hao DP, Xu JX, Huang CC, et al. Parallel CNN-Deep Learning Clinical-Imaging Signature for Assessing Pathologic Grade and Prognosis of Soft Tissue Sarcoma Patients. *J Magn Reson Imaging* 2024.

85. Hajianfar G, Sabouri M, Salimi Y, Amini M, Bagheri S, Jenabi E, et al. Artificial intelligence-based analysis of whole-body bone scintigraphy: The quest for the optimal deep learning algorithm and comparison with human observer performance. *Z Med Phys* 2024; **34**(2): 242-57.

86. Hao J, Liu S, Wang T, Han X, Gao A, Wang H, Hao D. Differentiation of malignant from benign soft tissue tumors using radiomics based on pharmacokinetic parameter maps obtained from dynamic contrast-enhanced magnetic resonance imaging data. *Chin J Acad Radiol* 2024.

87. He F, Xie L, Sun X, Xu J, Li Y, Liu R, et al. A Scoring System for Predicting Neoadjuvant Chemotherapy Response in Primary High-Grade Bone Sarcomas: A Multicenter Study. *Orthop Surg* 2022; **14**(10): 2499-509.

88. He J, Bi X. Automatic classification of spinal osteosarcoma and giant cell tumor of bone using optimized DenseNet. *J Bone Oncol* 2024; **46**: 100606.

89. He Q, Bano S, Liu J, Liu W, Stoyanov D, Zuo S. Query2: Query over queries for improving gastrointestinal stromal tumour detection in an endoscopic ultrasound. *Comput Biol Med* 2023; **152**: 106424.

90. He Y, Guo J, Ding X, van Ooijen PMA, Zhang Y, Chen A, et al. Convolutional neural network to predict the local recurrence of giant cell tumor of bone after curettage based on pre-surgery magnetic resonance images. *Eur Radiol* 2019; **29**(10): 5441-51.

91. He Y, Pan I, Bao B, Halsey K, Chang M, Liu H, et al. Deep learning-based classification of primary bone tumors on radiographs: A preliminary study. *EBioMedicine* 2020; **62**.

92. Hermessi H, Mourali O, Zagrouba E. Deep feature learning for soft tissue sarcoma classification in MR images via transfer learning. *Expert Syst Appl* 2019; **120**: 116-27.

93. Hu P, Chen L, Zhou Z. Machine Learning in the Differentiation of Soft Tissue Neoplasms: Comparison of Fat-Suppressed T2WI and Apparent Diffusion Coefficient (ADC) Features-Based Models. *J Digit Imaging* 2021; **34**(5): 1146-55.

94. Hu Y, Tang J, Zhao SH, Li Y. Diffusion-Weighted Imaging-Magnetic Resonance Imaging Information under Class-Structured Deep Convolutional Neural Network Algorithm in the Prognostic Chemotherapy of Osteosarcoma. *Sci Program* 2021; **2021**.

95. Hu Y, Wang H, Yue Z, Wang X, Wang Y, Luo Y, Jiang W. A contrast-enhanced MRI-based nomogram to identify lung metastasis in soft-tissue sarcoma: A multi-centre study. *Med Phys* 2023; **50**(5): 2961-70.

96. Hu Z, Liang H, Zhao H, Hou F, Hao D, Ji Q, et al. Preoperative contrast-enhanced CT-based radiomics signature for predicting hypoxia-inducible factor 1alpha expression in retroperitoneal sarcoma. *Clin Radiol* 2023; **78**(8): e543-e51.

97. Huang B, Wang J, Sun M, Chen X, Xu D, Li ZP, et al. Feasibility of multi-parametric magnetic resonance imaging combined with machine learning in the assessment of necrosis of osteosarcoma after neoadjuvant chemotherapy: a preliminary study. *BMC Cancer* 2020; **20**(1): 322.

98. Iwai T, Kida M, Okuwaki K, Watanabe M, Adachi K, Ishizaki J, et al. Deep learning analysis for differential diagnosis and risk classification of gastrointestinal tumors. *Scand J Gastroenterol* 2024: 1-8.

99. Jansma C, Wan X, Acem I, Spaanderman DJ, Visser JJ, Hanff D, et al. Preoperative Classification of Peripheral Nerve Sheath Tumors on MRI Using Radiomics. *Cancers (Basel)* 2024; **16**(11).

100. Jayachandran JJB, Ambigapathy S. X-Ray Image Analysis in Identification of Bone Cancer Using Laws Features and Machine Learning Model. *… Artificial Intelligence …* 2022.

101. Jeong SY, Kim W, Byun BH, Kong CB, Song WS, Lim I, et al. Prediction of Chemotherapy Response of Osteosarcoma Using Baseline 18F-FDG Textural Features Machine Learning Approaches with PCA. *Contrast Media Mol Imaging* 2019; **2019**: 3515080.

102. Ji X, Shang Y, Tan L, Hu Y, Liu J, Song L, et al. Prediction of High-Risk Gastrointestinal Stromal Tumor Recurrence Based on Delta-CT Radiomics Modeling: A 3-Year Follow-up Study After Surgery. *Clin Med Insights Oncol* 2024; **18**: 11795549241245698.

103. Jia X, Wan L, Chen X, Ji W, Huang S, Qi Y, et al. Risk stratification for 1- to 2-cm gastric gastrointestinal stromal tumors: visual assessment of CT and EUS high-risk features versus CT radiomics analysis. *Eur Radiol* 2023; **33**(4): 2768-78.

104. Joo DC, Kim GH, Lee MW, Lee BE, Kim JW, Kim KB. Artificial Intelligence-Based Diagnosis of Gastric Mesenchymal Tumors Using Digital Endosonography Image Analysis. *J Clin Med* 2024; **13**(13).

105. Juntu J, Sijbers J, De Backer S, Rajan J, Van Dyck D. Machine learning study of several classifiers trained with texture analysis features to differentiate benign from malignant soft-tissue tumors in T1-MRI images. *J Magn Reson Imaging* 2010; **31**(3): 680-9.

106. Kang B, Yuan X, Wang H, Qin S, Song X, Yu X, et al. Preoperative CT-Based Deep Learning Model for Predicting Risk Stratification in Patients With Gastrointestinal Stromal Tumors. *Front oncol* 2021; **11**: 750875.

107. Kim BC, Kim J, Kim K, Byun BH, Lim I, Kong CB, et al. Preliminary Radiogenomic Evidence for the Prediction of Metastasis and Chemotherapy Response in Pediatric Patients with Osteosarcoma Using (18)F-FDF PET/CT, EZRIN and KI67. *Cancers (Basel)* 2021; **13**(11).

108. Kim H, Rha SE, Shin YR, Kim EH, Park SY, Lee SL, et al. Differentiating Uterine Sarcoma From Atypical Leiomyoma on Preoperative Magnetic Resonance Imaging Using Logistic Regression Classifier: Added Value of Diffusion-Weighted Imaging-Based Quantitative Parameters. *Korean J Radiol* 2024; **25**(1): 43-54.

109. Kim J, Jeong SY, Kim BC, Byun BH, Lim I, Kong CB, et al. Prediction of Neoadjuvant Chemotherapy Response in Osteosarcoma Using Convolutional Neural Network of Tumor Center (18)F-FDG PET Images. *Diagnostics (Basel)* 2021; **11**(11).

110. Kim W, Park J, Sheen H, Byun BH, Lim I, Kong CB, et al. Development of deep learning model for prediction of chemotherapy response using PET images and radiomics features. 2018.

111. Kim YH, Kim GH, Kim KB, Lee MW, Lee BE, Baek DH, et al. Application of A Convolutional Neural Network in The Diagnosis of Gastric Mesenchymal Tumors on Endoscopic Ultrasonography Images. *J Clin Med* 2020; **9**(10).

112. Kumar R, Suhas MV. Classification of Benign and Malignant bone lesions on CT ImagesUsing Support Vector Machine: A Comparison of Kernel Functions. 2016: 821-4.

113. Kumar VDA. Bone Cancer Detection Using Feature Extraction with Classification Using K-Nearest Neighbor and Decision Tree Algorithm. *Smart Intelligent Computing and Communication …* 2021.

114. Lee S, Jung JY, Nam Y, Jung CK, Lee SY, Lee J, et al. Diagnosis of Marginal Infiltration in Soft Tissue Sarcoma by Radiomics Approach Using T2-Weighted Dixon Sequence. *J Magn Reson Imaging* 2023; **57**(3): 752-60.

115. Lee S, Lee SY, Jung JY, Nam Y, Jeon HJ, Jung CK, et al. Ensemble learning-based radiomics with multi-sequence magnetic resonance imaging for benign and malignant soft tissue tumor differentiation. *PLoS ONE* 2023; **18**(5): e0286417.

116. Lee SE, Jung JY, Nam Y, Lee SY, Park H, Shin SH, et al. Radiomics of diffusion-weighted MRI compared to conventional measurement of apparent diffusion-coefficient for differentiation between benign and malignant soft tissue tumors. *Sci rep* 2021; **11**(1): 15276.

117. Leporq B, Bouhamama A, Pilleul F, Lame F, Bihane C, Sdika M, et al. MRI-based radiomics to predict lipomatous soft tissue tumors malignancy: a pilot study. *Cancer Imaging* 2020; **20**(1): 78.

118. Li A, Hu Y, Cui XW, Ye XH, Peng XJ, Lv WZ, Zhao CK. Predicting the malignancy of extremity soft-tissue tumors by an ultrasound-based radiomics signature. *Acta Radiol* 2024; **65**(5): 470-81.

119. Li J, Li S, Li X, Miao S, Dong C, Gao C, et al. Primary bone tumor detection and classification in full-field bone radiographs via YOLO deep learning model. *Eur Radiol* 2023; **33**(6): 4237-48.

120. Li L, Wang K, Ma X, Liu Z, Wang S, Du J, et al. Radiomic analysis of multiparametric magnetic resonance imaging for differentiating skull base chordoma and chondrosarcoma. *Eur J Radiol* 2019; **118**: 81-7.

121. Li Q, Wang N, Wang Y, Li X, Su Q, Zhang J, et al. Intratumoral and peritumoral CT radiomics in predicting prognosis in patients with chondrosarcoma: a multicenter study. *Insights imaging* 2024; **15**(1): 9.

122. Li X, Jiang F, Guo Y, Jin Z, Wang Y. Computer-aided diagnosis of gastrointestinal stromal tumors: a radiomics method on endoscopic ultrasound image. *Int j comput assist radiol surg* 2019; **14**(10): 1635-45.

123. Li X, Lan M, Wang X, Zhang J, Gong L, Liao F, et al. Development and validation of a MRI-based combined radiomics nomogram for differentiation in chondrosarcoma. *Front oncol* 2023; **13**: 1090229.

124. Li X, Shi X, Wang Y, Pang J, Zhao X, Xu Y, et al. A CT-based radiomics nomogram for predicting histologic grade and outcome in chondrosarcoma. *Cancer Imaging* 2024; **24**(1): 50.

125. Li X, Zhang J, Leng Y, Liu J, Li L, Wan T, et al. Preoperative prediction of histopathological grading in patients with chondrosarcoma using MRI-based radiomics with semantic features. *BMC med imaging* 2024; **24**(1): 171.

126. Liang HY, Yang SF, Zou HM, Hou F, Duan LS, Huang CC, et al. Deep Learning Radiomics Nomogram to Predict Lung Metastasis in Soft-Tissue Sarcoma: A Multi-Center Study. *Front oncol* 2022; **12**: 897676.

127. Lin JX, Wang FH, Wang ZK, Wang JB, Zheng CH, Li P, et al. Prediction of the mitotic index and preoperative risk stratification of gastrointestinal stromal tumors with CT radiomic features. *Radiol Med (Torino)* 2023; **128**(6): 644-54.

128. Lin P, Yang PF, Chen S, Shao YY, Xu L, Wu Y, et al. A Delta-radiomics model for preoperative evaluation of Neoadjuvant chemotherapy response in high-grade osteosarcoma. *Cancer Imaging* 2020; **20**(1): 7.

129. Lingappa E, Parvathy LR. Image Classification with Deep Learning Methods for Detecting and Staging Bone Cancer from MRI. *2023 International Conference on …* 2023.

130. Liu B, Liu H, Zhang L, Song Y, Yang S, Zheng Z, et al. Value of contrast-enhanced CT based radiomic machine learning algorithm in differentiating gastrointestinal stromal tumors with KIT exon 11 mutation: a two-center study. *Diagn Interv Radiol* 2022; **28**(1): 29-38.

131. Liu C, Guo Y, Jiang F, Xu L, Shen F, Jin Z, Wang Y. Gastrointestinal stromal tumors diagnosis on multi-center endoscopic ultrasound images using multi-scale image normalization and transfer learning. *Technol Health Care* 2022; **30**(S1): 47-59.

132. Liu C, Qiao M, Jiang F, Guo Y, Jin Z, Wang Y. TN-USMA Net: Triple normalization-based gastrointestinal stromal tumors classification on multicenter EUS images with ultrasound-specific pretraining and meta attention. *Med Phys* 2021; **48**(11): 7199-214.

133. Liu H, Jiao M, Yuan Y, Ouyang H, Liu J, Li Y, et al. Benign and malignant diagnosis of spinal tumors based on deep learning and weighted fusion framework on MRI. *Insights imaging* 2022; **13**(1): 87.

134. Liu J, Huang J, Song Y, He Q, Fang W, Wang T, et al. Differentiating Gastrointestinal Stromal Tumors From Leiomyomas of Upper Digestive Tract Using Convolutional Neural Network Model by Endoscopic Ultrasonography. *J Clin Gastroenterol* 2024; **58**(6): 574-9.

135. Liu J, Lian T, Chen H, Wang X, Quan X, Deng Y, et al. Pretreatment Prediction of Relapse Risk in Patients with Osteosarcoma Using Radiomics Nomogram Based on CT: A Retrospective Multicenter Study. *Biomed Res Int* 2021; **2021**: 6674471.

136. Liu L, Chen J, Shan J, Sun X. Development and validation of an EUS-based nomogram for prediction of the malignant potential in gastrointestinal stromal tumors. *Scand J Gastroenterol* 2023; **58**(7): 830-7.

137. Liu M, Bian J. Radiomics signatures based on contrast-enhanced CT for preoperative prediction of the Ki-67 proliferation state in gastrointestinal stromal tumors. *Jpn J Radiol* 2023; **41**(7): 741-51.

138. Liu R, Pan D, Xu Y, Zeng H, He Z, Lin J, et al. A deep learning–machine learning fusion approach for the classification of benign, malignant, and intermediate bone tumors. *Eur Radiol* 2022; **32**(2): 1371-83.

139. Liu S, Sun W, Yang S, Duan L, Huang C, Xu J, et al. Deep learning radiomic nomogram to predict recurrence in soft tissue sarcoma: a multi-institutional study. *Eur Radiol* 2022; **32**(2): 793-805.

140. Liu X, Guo L, Wang H, Guo J, Yang S, Duan L. Research on imbalance machine learning methods for MR[Formula: see text]WI soft tissue sarcoma data. *BMC med imaging* 2022; **22**(1): 149.

141. Liu X, Yin Y, Wang X, Yang C, Wan S, Yin X, et al. Gastrointestinal stromal tumors: associations between contrast-enhanced CT images and KIT exon 11 gene mutation. *Ann transl med* 2021; **9**(19): 1496.

142. Liu Y, He C, Fang W, Peng L, Shi F, Xia Y, et al. Prediction of Ki-67 expression in gastrointestinal stromal tumors using radiomics of plain and multiphase contrast-enhanced CT. *Eur Radiol* 2023; **33**(11): 7609-17.

143. Liu Y, Yin P, Cui J, Sun C, Chen L, Hong N. Postoperative Relapse Prediction in Patients With Ewing Sarcoma Using Computed Tomography-Based Radiomics Models Covering Tumor Per Se and Peritumoral Signatures. *J Comput Assist Tomogr* 2023; **47**(5): 766-73.

144. Liu Y, Yin P, Cui J, Sun C, Chen L, Hong N, Li Z. Radiomics analysis based on CT for the prediction of pulmonary metastases in ewing sarcoma. *BMC med imaging* 2023; **23**(1): 147.

145. Lombardi A, Arezzo F, Di Sciascio E, Ardito C, Mongelli M, Di Lillo N, et al. A human-interpretable machine learning pipeline based on ultrasound to support leiomyosarcoma diagnosis. *Artif Intell Med* 2023; **146**: 102697.

146. Long B, Zhang H, Zhang H, Chen W, Sun Y, Tang R, et al. Deep learning models of ultrasonography significantly improved the differential diagnosis performance for superficial soft-tissue masses: a retrospective multicenter study. *BMC Med* 2023; **21**(1): 405.

147. Lu Y, Chen L, Wu J, Er L, Shi H, Cheng W, et al. Artificial intelligence in endoscopic ultrasonography: risk stratification of gastric gastrointestinal stromal tumors. *Therap adv in gastroenterol* 2023; **16**: 17562848231177156.

148. Lu Y, Wu J, Hu M, Zhong Q, Er L, Shi H, et al. Artificial Intelligence in the Prediction of Gastrointestinal Stromal Tumors on Endoscopic Ultrasonography Images: Development, Validation and Comparison with Endosonographers. *Gut Liver* 2023; **17**(6): 874-83.

149. Luo Z, Li J, Liao Y, Huang W, Li Y, Shen X. Prediction of response to preoperative neoadjuvant chemotherapy in extremity high-grade osteosarcoma using X-ray and multiparametric MRI radiomics. *JOURNAL OF X-RAY SCIENCE AND TECHNOLOGY* 2023; **31**(3): 611-26.

150. Luo Z, Li J, Liao Y, Liu R, Shen X, Chen W. Radiomics Analysis of Multiparametric MRI for Prediction of Synchronous Lung Metastases in Osteosarcoma. *Front oncol* 2022; **12**: 802234.

151. Lv C, Chen H, Huang P, Chen Y, Liu B. Application of Computer-Assisted Endoscopic Ultrasonography Based on Texture Features in Differentiating Gastrointestinal Stromal Tumors from Benign Gastric Mesenchymal Tumors. *Turk J Gastroenterol* 2024; **35**(5): 366-73.

152. Malek M, Gity M, Alidoosti A, Oghabian Z, Rahimifar P, Seyed Ebrahimi SM, et al. A machine learning approach for distinguishing uterine sarcoma from leiomyomas based on perfusion weighted MRI parameters. *Eur J Radiol* 2019; **110**: 203-11.

153. Malinauskaite I, Hofmeister J, Burgermeister S, Neroladaki A, Hamard M, Montet X, Boudabbous S. Radiomics and Machine Learning Differentiate Soft-Tissue Lipoma and Liposarcoma Better than Musculoskeletal Radiologists. *SARCOMA* 2020; **2020**: 7163453.

154. Mao H, Zhang B, Zou M, Huang Y, Yang L, Wang C, et al. MRI-Based Radiomics Models for Predicting Risk Classification of Gastrointestinal Stromal Tumors. *Front oncol* 2021; **11**: 631927.

155. Martin-Carreras T, Li H, Cooper K, Fan Y, Sebro R. Radiomic features from MRI distinguish myxomas from myxofibrosarcomas. *BMC med imaging* 2019; **19**(1): 67.

156. Mayerhoefer ME, Breitenseher M, Amann G, Dominkus M. Are signal intensity and homogeneity useful parameters for distinguishing between benign and malignant soft tissue masses on MR images? Objective evaluation by means of texture analysis. *Magn Reson Imaging* 2008; **26**(9): 1316-22.

157. Mazumder MH, Singh MP. Bone Cancer Detection Using Deep Learning. *… on Innovations in Computer Science and …* 2022.

158. Megala G, Swarnalatha P, Venkatesan R. Detecting Bone Tumor on Applying Edge Computational Deep Learning Approach. *International Conference on …* 2023.

159. Minoda Y, Ihara E, Fujimori N, Nagatomo S, Esaki M, Hata Y, et al. Efficacy of ultrasound endoscopy with artificial intelligence for the differential diagnosis of non-gastric gastrointestinal stromal tumors. *Sci rep* 2022; **12**(1): 16640.

160. Minoda Y, Ihara E, Komori K, Ogino H, Otsuka Y, Chinen T, et al. Efficacy of endoscopic ultrasound with artificial intelligence for the diagnosis of gastrointestinal stromal tumors. *J Gastroenterol* 2020; **55**(12): 1119-26.

161. Mutlu IN, Kocak B, Kus EA, Ulusan MB, Kilickesmez O. Machine Learning-Based Computed Tomography Texture Analysis of Lytic Bone Lesions Needing Biopsy: A Preliminary Study. *Istanb Med J* 2021; **22**(3): 223-31.

162. Nakagawa M, Nakaura T, Namimoto T, Iyama Y, Kidoh M, Hirata K, et al. A multiparametric MRI-based machine learning to distinguish between uterine sarcoma and benign leiomyoma: comparison with 18F-FDG PET/CT. *Clin Radiol* 2019; **74**(2): 167.e1-.e.

163. Nakagawa M, Nakaura T, Namimoto T, Iyama Y, Kidoh M, Hirata K, et al. Machine Learning to Differentiate T2-Weighted Hyperintense Uterine Leiomyomas from Uterine Sarcomas by Utilizing Multiparametric Magnetic Resonance Quantitative Imaging Features. *Acad Radiol* 2019; **26**(10): 1390-9.

164. Nakagawa M, Nakaura T, Yoshida N, Azuma M, Uetani H, Nagayama Y, et al. Performance of Machine Learning Methods Based on Multi-Sequence Textural Parameters Using Magnetic Resonance Imaging and Clinical Information to Differentiate Malignant and Benign Soft Tissue Tumors. *Acad Radiol* 2023; **30**(1): 83-92.

165. Navarro F, Dapper H, Asadpour R, Knebel C, Spraker MB, Schwarze V, et al. Development and External Validation of Deep-Learning-Based Tumor Grading Models in Soft-Tissue Sarcoma Patients Using MR Imaging. *Cancers (Basel)* 2021; **13**(12).

166. Nguyen VX, Nguyen CC, Li B, Das A. Digital image analysis is a useful adjunct to endoscopic ultrasonographic diagnosis of subepithelial lesions of the gastrointestinal tract. *J Ultrasound Med* 2010; **29**(9): 1345-51.

167. Nie P, Zhao X, Wang N, Ma J, Zuo P, Hao D, Yu T. A Computed Tomography Radiomics Nomogram in Differentiating Chordoma From Giant Cell Tumor in the Axial Skeleton. *J Comput Assist Tomogr* 2023; **47**(3): 453-9.

168. Ning Z, Luo J, Li Y, Han S, Feng Q, Xu Y, et al. Pattern Classification for Gastrointestinal Stromal Tumors by Integration of Radiomics and Deep Convolutional Features. *IEEE j biomed health inform* 2019; **23**(3): 1181-91.

169. Oh CK, Kim T, Cho YK, Cheung DY, Lee BI, Cho YS, et al. Convolutional neural network-based object detection model to identify gastrointestinal stromal tumors in endoscopic ultrasound images. *J Gastroenterol Hepatol* 2021; **36**(12): 3387-94.

170. Ouyang H, Meng F, Liu J, Song X, Li Y, Yuan Y, et al. Evaluation of Deep Learning-Based Automated Detection of Primary Spine Tumors on MRI Using the Turing Test. *Front Oncol* 2022; **12**.

171. Pan D, Liu R, Zheng B, Yuan J, Zeng H, He Z, et al. Using Machine Learning to Unravel the Value of Radiographic Features for the Classification of Bone Tumors. *BioMed Res Int* 2021; **2021**.

172. Pan J, Zhang K, Le H, Jiang Y, Li W, Geng Y, et al. Radiomics Nomograms Based on Non-enhanced MRI and Clinical Risk Factors for the Differentiation of Chondrosarcoma from Enchondroma. *J Magn Reson Imaging* 2021; **54**(4): 1314-23.

173. Park CW, Oh SJ, Kim KS, Jang MC, Kim IS, Lee YK, et al. Artificial intelligence-based classification of bone tumors in the proximal femur on plain radiographs: System development and validation. *PLoS ONE* 2022; **17**(2 February).

174. Peeken JC, Asadpour R, Specht K, Chen EY, Klymenko O, Akinkuoroye V, et al. MRI-based delta-radiomics predicts pathologic complete response in high-grade soft-tissue sarcoma patients treated with neoadjuvant therapy. *Radiother Oncol* 2021; **164**: 73-82.

175. Peeken JC, Bernhofer M, Spraker MB, Pfeiffer D, Devecka M, Thamer A, et al. CT-based radiomic features predict tumor grading and have prognostic value in patients with soft tissue sarcomas treated with neoadjuvant radiation therapy. *Radiother Oncol* 2019; **135**: 187-96.

176. Peeken JC, Neumann J, Asadpour R, Leonhardt Y, Moreira JR, Hippe DS, et al. Prognostic Assessment in High-Grade Soft-Tissue Sarcoma Patients: A Comparison of Semantic Image Analysis and Radiomics. *Cancers (Basel)* 2021; **13**(8).

177. Peeken JC, Spraker MB, Knebel C, Dapper H, Pfeiffer D, Devecka M, et al. Tumor grading of soft tissue sarcomas using MRI-based radiomics. *EBioMedicine* 2019; **48**: 332-40.

178. Peng Y, Bi L, Guo Y, Feng D, Fulham M, Kim J. Deep multi-modality collaborative learning for distant metastases predication in PET-CT soft-tissue sarcoma studies. *Annu Int Conf IEEE Eng Med Biol Soc* 2019; **2019**: 3658-88.

179. Peng Y, Bi L, Kumar A, Fulham M, Feng D, Kim J. Predicting distant metastases in soft-tissue sarcomas from PET-CT scans using constrained hierarchical multi-modality feature learning. *Phys Med Biol* 2021; **66**(24).

180. Pereira HM, Leite Duarte ME, Ribeiro Damasceno I, de Oliveira Moura Santos LA, Nogueira-Barbosa MH. Machine learning-based CT radiomics features for the prediction of pulmonary metastasis in osteosarcoma. *Br J Radiol* 2021; **94**(1124): 20201391.

181. Pressney I, Khoo M, Endozo R, Ganeshan B, O'Donnell P. Pilot study to differentiate lipoma from atypical lipomatous tumour/well-differentiated liposarcoma using MR radiomics-based texture analysis. *Skeletal Radiol* 2020; **49**(11): 1719-29.

182. Purnima S, Lashna V, Mahalakshmi R. An Approach to Detect and Classify Bone tumour using fast and Robust Fuzzy C Means Clustering technique. *Annals of the …* 2021.

183. Ren C, Wang S, Zhang S. Development and validation of a nomogram based on CT images and 3D texture analysis for preoperative prediction of the malignant potential in gastrointestinal stromal tumors. *Cancer Imaging* 2020; **20**(1): 5.

184. Rengo M, Onori A, Caruso D, Bellini D, Carbonetti F, De Santis D, et al. Development and Validation of Artificial-Intelligence-Based Radiomics Model Using Computed Tomography Features for Preoperative Risk Stratification of Gastrointestinal Stromal Tumors. *J Pers Med* 2023; **13**(5).

185. Ristow I, Madesta F, Well L, Shenas F, Wright F, Molwitz I, et al. Evaluation of magnetic resonance imaging-based radiomics characteristics for differentiation of benign and malignant peripheral nerve sheath tumors in neurofibromatosis type 1. *Neuro-oncol* 2022; **24**(10): 1790-8.

186. Roller LA, Wan Q, Liu X, Qin L, Chapel D, Burk KS, et al. MRI, clinical, and radiomic models for differentiation of uterine leiomyosarcoma and leiomyoma. *Abdom Radiol* 2024; **49**(5): 1522-33.

187. Sampath K, Rajagopal S, Chintanpalli A. A comparative analysis of CNN-based deep learning architectures for early diagnosis of bone cancer using CT images. *Sci rep* 2024; **14**(1): 2144.

188. Santoro M, Zybin V, Coada CA, Mantovani G, Paolani G, Di Stanislao M, et al. Machine Learning Applied to Pre-Operative Computed-Tomography-Based Radiomic Features Can Accurately Differentiate Uterine Leiomyoma from Leiomyosarcoma: A Pilot Study. *Cancers (Basel)* 2024; **16**(8).

189. Seven G, Silahtaroglu G, Kochan K, Ince AT, Arici DS, Senturk H. Use of Artificial Intelligence in the Prediction of Malignant Potential of Gastric Gastrointestinal Stromal Tumors. *Dig Dis Sci* 2022; **67**(1): 273-81.

190. Seven G, Silahtaroglu G, Seven OO, Senturk H. Differentiating Gastrointestinal Stromal Tumors from Leiomyomas Using a Neural Network Trained on Endoscopic Ultrasonography Images. *Dig Dis* 2022; **40**(4): 427-35.

191. Shang S, Sun J, Yue Z, Wang Y, Wang X, Luo Y, et al. Multi-parametric MRI based radiomics with tumor subregion partitioning for differentiating benign and malignant soft-tissue tumors. *Biomed Signal Process Control* 2021; **67**.

192. Shao J, Lin H, Ding L, Li B, Xu D, Sun Y, et al. Deep learning for differentiation of osteolytic osteosarcoma and giant cell tumor around the knee joint on radiographs: a multicenter study. *Insights imaging* 2024; **15**(1): 35.

193. Shao J, Wang C, Shu K, Zhou Y, Cheng N, Lai Z, et al. A contrast-enhanced CT-based radiomic nomogram for the differential diagnosis of intravenous leiomyomatosis and uterine leiomyoma. *Front oncol* 2023; **13**: 1239124.

194. Shao M, Niu Z, He L, Fang Z, He J, Xie Z, et al. Building Radiomics Models Based on Triple-Phase CT Images Combining Clinical Features for Discriminating the Risk Rating in Gastrointestinal Stromal Tumors. *Front oncol* 2021; **11**: 737302.

195. Sharma A, Yadav DP, Garg H, Kumar M, Sharma B, Koundal D. Bone Cancer Detection Using Feature Extraction Based Machine Learning Model. *Comput math methods med* 2021; **2021**: 7433186.

196. Sheen H, Kim W, Byun BH, Kong CB, Song WS, Cho WH, et al. Metastasis risk prediction model in osteosarcoma using metabolic imaging phenotypes: A multivariable radiomics model. *PLoS ONE* 2019; **14**(11): e0225242.

197. Shen R, Li Z, Zhang L, Hua Y, Mao M, Li Z, et al. Osteosarcoma Patients Classification Using Plain X-Rays and Metabolomic Data. *Conf Proc IEEE Eng Med Biol Soc* 2018; **2018**: 690-3.

198. Shrivastava A, Nag MK. Enhancing Bone Cancer Diagnosis Through Image Extraction and Machine Learning: A State-of-the-Art Approach. *Surg Innov* 2024; **31**(1): 58-70.

199. Sierra ED, Valenzuela R, Canjirathinkal MA, Costelloe CM, Moradi H, Madewell JE, et al. Cancer Radiomic and Perfusion Imaging Automated Framework: Validation on Musculoskeletal Tumors. *JCO Clin Cancer Inform* 2024; **8**: e2300118.

200. Singh M, Angurala M, Bala M. Bone Tumour detection Using Feature Extraction with Classification by Deep Learning Techniques. *Research Journal of Computer …* 2020.

201. Skorpil M, Ryden H, Berglund J, Brynolfsson P, Brosjo O, Tsagozis P. Soft-tissue fat tumours: differentiating malignant from benign using proton density fat fraction quantification MRI. *Clin Radiol* 2019; **74**(7): 534-8.

202. Song Y, Li J, Wang H, Liu B, Yuan C, Liu H, et al. Radiomics Nomogram Based on Contrast-enhanced CT to Predict the Malignant Potential of Gastrointestinal Stromal Tumor: A Two-center Study. *Acad Radiol* 2022; **29**(6): 806-16.

203. Spraker MB, Wootton LS, Hippe DS, Ball KC, Peeken JC, Macomber MW, et al. MRI Radiomic Features Are Independently Associated With Overall Survival in Soft Tissue Sarcoma. *Adv Radiat Oncol* 2019; **4**(2): 413-21.

204. Starmans MPA, Timbergen MJM, Vos M, Renckens M, Grunhagen DJ, van Leenders G, et al. Differential Diagnosis and Molecular Stratification of Gastrointestinal Stromal Tumors on CT Images Using a Radiomics Approach. *J Digit Imaging* 2022; **35**(2): 127-36.

205. Su Q, Wang N, Wang B, Wang Y, Dai Z, Zhao X, et al. Ct-based intratumoral and peritumoral radiomics for predicting prognosis in osteosarcoma: A multicenter study. *Eur J Radiol* 2024; **172**: 111350.

206. Sudjai N, Siriwanarangsun P, Lektrakul N, Saiviroonporn P, Maungsomboon S, Phimolsarnti R, et al. Robustness of Radiomic Features: Two-Dimensional versus Three-Dimensional MRI-Based Feature Reproducibility in Lipomatous Soft-Tissue Tumors. *Diagnostics (Basel)* 2023; **13**(2).

207. Sudjai N, Siriwanarangsun P, Lektrakul N, Saiviroonporn P, Maungsomboon S, Phimolsarnti R, et al. Tumor-to-bone distance and radiomic features on MRI distinguish intramuscular lipomas from well-differentiated liposarcomas. *J ORTHOP SURG* 2023; **18**(1): 255.

208. Sun K, Yu S, Wang Y, Jia R, Shi R, Liang C, et al. Development of a multi-phase CT-based radiomics model to differentiate heterotopic pancreas from gastrointestinal stromal tumor. *BMC med imaging* 2024; **24**(1): 44.

209. Sun W, Liu S, Guo J, Liu S, Hao D, Hou F, et al. A CT-based radiomics nomogram for distinguishing between benign and malignant bone tumours. *Cancer Imaging* 2021; **21**(1): 20.

210. Sun XF, Zhu HT, Ji WY, Zhang XY, Li XT, Tang L, Sun YS. Preoperative prediction of malignant potential of 2-5 cm gastric gastrointestinal stromal tumors by computerized tomography-based radiomics. *World J Gastrointest Oncol* 2022; **14**(5): 1014-26.

211. Tagliafico AS, Bignotti B, Rossi F, Valdora F, Martinoli C. Local recurrence of soft tissue sarcoma: a radiomic analysis. *RADIOL ONCOL* 2019; **53**(3): 300-6.

212. Tamehisa T, Sato S, Sakai T, Maekawa R, Tanabe M, Ito K, Sugino N. Establishment of Noninvasive Prediction Models for the Diagnosis of Uterine Leiomyoma Subtypes. *Obstet Gynecol* 2024; **143**(3): 358-65.

213. Tang Y, Cui J, Zhu J, Fan G. Differentiation Between Lipomas and Atypical Lipomatous Tumors of the Extremities Using Radiomics. *J Magn Reson Imaging* 2022; **56**(6): 1746-54.

214. Teo KY, Daescu O, Cederberg K, Sengupta A, Leavey PJ. Correlation of histopathology and multi-modal magnetic resonance imaging in childhood osteosarcoma: Predicting tumor response to chemotherapy. *PLoS ONE* 2022; **17**(2): e0259564.

215. Thornhill RE, Golfam M, Sheikh A, Cron GO, White EA, Werier J, et al. Differentiation of lipoma from liposarcoma on MRI using texture and shape analysis. *Acad Radiol* 2014; **21**(9): 1185-94.

216. Tian L, Li X, Zheng H, Wang L, Qin Y, Cai J. Fisher discriminant model based on LASSO logistic regression for computed tomography imaging diagnosis of pelvic rhabdomyosarcoma in children. *Sci rep* 2022; **12**(1): 15631.

217. Tian L, Zhang D, Bao S, Nie P, Hao D, Liu Y, et al. Radiomics-based machine-learning method for prediction of distant metastasis from soft-tissue sarcomas. *Clin Radiol* 2021; **76**(2): 158.e19-.e25.

218. Tian Z, Cheng Y, Zhao S, Li R, Zhou J, Sun Q, Wang D. Deep learning radiomics-based prediction model of metachronous distant metastasis following curative resection for retroperitoneal leiomyosarcoma: a bicentric study. *Cancer Imaging* 2024; **24**(1): 52.

219. Timbergen MJM, Starmans MPA, Padmos GA, Grunhagen DJ, van Leenders G, Hanff DF, et al. Differential diagnosis and mutation stratification of desmoid-type fibromatosis on MRI using radiomics. *Eur J Radiol* 2020; **131**: 109266.

220. Toyohara Y, Sone K, Noda K, Yoshida K, Kato S, Kaiume M, et al. The automatic diagnosis artificial intelligence system for preoperative magnetic resonance imaging of uterine sarcoma. *J gynecol oncol* 2024; **35**(3): e24.

221. Toyohara Y, Sone K, Noda K, Yoshida K, Kurokawa R, Tanishima T, et al. Development of a deep learning method for improving diagnostic accuracy for uterine sarcoma cases. *Sci rep* 2022; **12**(1): 19612.

222. Usuff R, Kothandapani S, Rangan R. Enhancing radiographic image interpretation: WARES-PRS model for knee bone tumour detection. *… in Neural Systems* 2024.

223. Vaiyapuri T, Balaji P, Shridevi S, Dharmarajlu SM, Alaseem NA. An attention-based bidirectional long short-term memory based optimal deep learning technique for bone cancer detection and classifications. *Aims Mathematics* 2024; **9**(6): 16704-20.

224. Vallieres M, Freeman CR, Skamene SR, El Naqa I. A radiomics model from joint FDG-PET and MRI texture features for the prediction of lung metastases in soft-tissue sarcomas of the extremities. *Phys Med Biol* 2015; **60**(14): 5471-96.

225. von Schacky CE, Wilhelm NJ, Schäfer VS, Leonhardt Y, Gassert FG, Foreman SC, et al. Multitask deep learning for segmentation and classification of primary bone tumors on radiographs. *Radiology* 2021; **301**(2): 398-406.

226. von Schacky CE, Wilhelm NJ, Schafer VS, Leonhardt Y, Jung M, Jungmann PM, et al. Development and evaluation of machine learning models based on X-ray radiomics for the classification and differentiation of malignant and benign bone tumors. *Eur Radiol* 2022; **32**(9): 6247-57.

227. Vos M, Starmans MPA, Timbergen MJM, van der Voort SR, Padmos GA, Kessels W, et al. Radiomics approach to distinguish between well differentiated liposarcomas and lipomas on MRI. *Br J Surg* 2019; **106**(13): 1800-9.

228. Wahab CA, Jannot AS, Bonaffini PA, Bourillon C, Cornou C, Lefrere-Belda MA, et al. Diagnostic Algorithm to Differentiate Benign Atypical Leiomyomas from Malignant Uterine Sarcomas with Diffusion-weighted MRI (vol 297, pg 361, 2020). *Radiology* 2020; **297**(3): E347-E.

229. Wang B, Perronne L, Burke C, Adler RS. Artificial intelligence for classification of soft-tissue masses at us. *Radiology: Art Int* 2021; **3**(1).

230. Wang C, Li H, Jiaerken Y, Huang P, Sun L, Dong F, et al. Building CT Radiomics-Based Models for Preoperatively Predicting Malignant Potential and Mitotic Count of Gastrointestinal Stromal Tumors. *Transl Oncol* 2019; **12**(9): 1229-36.

231. Wang C, Zhang Z, Dou Y, Liu Y, Chen B, Liu Q, Wang S. Development of clinical and magnetic resonance imaging-based radiomics nomograms for the differentiation of nodular fasciitis from soft tissue sarcoma. *Acta Radiol* 2023; **64**(9): 2578-89.

232. Wang FH, Zheng HL, Li JT, Li P, Zheng CH, Chen QY, et al. Prediction of recurrence-free survival and adjuvant therapy benefit in patients with gastrointestinal stromal tumors based on radiomics features. *Radiol Med (Torino)* 2022; **127**(10): 1085-97.

233. Wang H, Chen H, Duan S, Hao D, Liu J. Radiomics and Machine Learning With Multiparametric Preoperative MRI May Accurately Predict the Histopathological Grades of Soft Tissue Sarcomas. *J Magn Reson Imaging* 2020; **51**(3): 791-7.

234. Wang H, Nie P, Wang Y, Xu W, Duan S, Chen H, et al. Radiomics nomogram for differentiating between benign and malignant soft-tissue masses of the extremities. *J Magn Reson Imaging* 2020; **51**(1): 155-63.

235. Wang H, Zhang J, Bao S, Liu J, Hou F, Huang Y, et al. Preoperative MRI-Based Radiomic Machine-Learning Nomogram May Accurately Distinguish Between Benign and Malignant Soft-Tissue Lesions: A Two-Center Study. *J Magn Reson Imaging* 2020; **52**(3): 873-82.

236. Wang J, Shao M, Hu H, Xiao W, Cheng G, Yang G, et al. Convolutional neural network applied to preoperative venous-phase CT images predicts risk category in patients with gastric gastrointestinal stromal tumors. *BMC Cancer* 2024; **24**(1): 280.

237. Wang J, Xie Z, Zhu X, Niu Z, Ji H, He L, et al. Differentiation of gastric schwannomas from gastrointestinal stromal tumors by CT using machine learning. *Abdom Radiol* 2021; **46**(5): 1773-82.

238. Wang JK. Predictive value and modeling analysis of MSCT signs in gastrointestinal stromal tumors (GISTs) to pathological risk degree. *Eur Rev Med Pharmacol Sci* 2017; **21**(5): 999-1005.

239. Wang M, Feng Z, Zhou L, Zhang L, Hao X, Zhai J. Computed-Tomography-Based Radiomics Model for Predicting the Malignant Potential of Gastrointestinal Stromal Tumors Preoperatively: A Multi-Classifier and Multicenter Study. *Front oncol* 2021; **11**: 582847.

240. Wang P, Yan J, Qiu H, Huang J, Yang Z, Shi Q, Yan C. A radiomics-clinical combined nomogram-based on non-enhanced CT for discriminating the risk stratification in GISTs. *J Cancer Res Clin Oncol* 2023; **149**(14): 12993-3003.

241. Wang Q, Zhang Y, Zhang E, Xing X, Chen Y, Nie K, et al. A Multiparametric Method Based on Clinical and CT-Based Radiomics to Predict the Expression of p53 and VEGF in Patients With Spinal Giant Cell Tumor of Bone. *Front oncol* 2022; **12**: 894696.

242. Wang Q, Zhang Y, Zhang E, Xing X, Chen Y, Su MY, Lang N. Prediction of the early recurrence in spinal giant cell tumor of bone using radiomics of preoperative CT: Long-term outcome of 62 consecutive patients. *J Bone Oncol* 2021; **27**: 100354.

243. Wang S, Sun M, Sun J, Wang Q, Wang G, Wang X, et al. Advancing musculoskeletal tumor diagnosis: Automated segmentation and predictive classification using deep learning and radiomics. *Comput Biol Med* 2024; **175**: 108502.

244. Wang Y, Wang Y, Ren J, Jia L, Ma L, Yin X, et al. Malignancy risk of gastrointestinal stromal tumors evaluated with noninvasive radiomics: A multi-center study. *Front oncol* 2022; **12**: 966743.

245. Wei CJ, Yan C, Tang Y, Wang W, Gu YH, Ren JY, et al. Computed Tomography-Based Differentiation of Benign and Malignant Craniofacial Lesions in Neurofibromatosis Type I Patients: A Machine Learning Approach. *Front oncol* 2020; **10**: 1192.

246. Wei Y, Lu Z, Ren Y. Predictive Value of a Radiomics Nomogram Model Based on Contrast-Enhanced Computed Tomography for KIT Exon 9 Gene Mutation in Gastrointestinal Stromal Tumors. *Technol Cancer Res Treat* 2023; **22**: 15330338231181260.

247. White LM, Atinga A, Naraghi AM, Lajkosz K, Wunder JS, Ferguson P, et al. T2-weighted MRI radiomics in high-grade intramedullary osteosarcoma: predictive accuracy in assessing histologic response to chemotherapy, overall survival, and disease-free survival. *Skeletal Radiol* 2023; **52**(3): 553-64.

248. Wu Y, Xu L, Yang P, Lin N, Huang X, Pan W, et al. Survival Prediction in High-grade Osteosarcoma Using Radiomics of Diagnostic Computed Tomography. *EBioMedicine* 2018; **34**: 27-34.

249. Xie H, Hu J, Zhang X, Ma S, Liu Y, Wang X. Preliminary utilization of radiomics in differentiating uterine sarcoma from atypical leiomyoma: Comparison on diagnostic efficacy of MRI features and radiomic features. *Eur J Radiol* 2019; **115**: 39-45.

250. Xie H, Zhang X, Ma S, Liu Y, Wang X. Preoperative Differentiation of Uterine Sarcoma from Leiomyoma: Comparison of Three Models Based on Different Segmentation Volumes Using Radiomics. *Mol Imaging Biol* 2019; **21**(6): 1157-64.

251. Xie H, Zhang Y, Dong L, Lv H, Li X, Zhao C, et al. Deep learning driven diagnosis of malignant soft tissue tumors based on dual-modal ultrasound images and clinical indexes. *Front oncol* 2024; **14**: 1361694.

252. Xie Z, Suo S, Zhang W, Zhang Q, Dai Y, Song Y, et al. Prediction of high Ki-67 proliferation index of gastrointestinal stromal tumors based on CT at non-contrast-enhanced and different contrast-enhanced phases. *Eur Radiol* 2024; **34**(4): 2223-32.

253. Xie Z, Zhao H, Song L, Ye Q, Zhong L, Li S, et al. A radiograph-based deep learning model improves radiologists’ performance for classification of histological types of primary bone tumors: A multicenter study. *Eur J Radiol* 2024; **176**.

254. Xu F, Ma X, Wang Y, Tian Y, Tang W, Wang M, et al. CT texture analysis can be a potential tool to differentiate gastrointestinal stromal tumors without KIT exon 11 mutation. *Eur J Radiol* 2018; **107**: 90-7.

255. Xu J, Miao L, Wang CX, Wang HH, Wang QZ, Li M, et al. Preoperative Contrast-Enhanced CT-Based Deep Learning Radiomics Model for Distinguishing Retroperitoneal Lipomas and Well-Differentiated Liposarcomas. *Acad Radiol* 2024; **31**(12): 5042-53.

256. Xu L, Wang MY, Qi L, Zou YF, Fei-Yun WU, Sun XL. Radiomics approach to distinguish between benign and malignant soft tissue tumors on magnetic resonance imaging. *Eur J Radiol Open* 2024; **12**: 100555.

257. Xu L, Yang P, Hu K, Wu Y, Xu-Welliver M, Wan Y, et al. Prediction of neoadjuvant chemotherapy response in high-grade osteosarcoma: added value of non-tumorous bone radiomics using CT images. *Quant imaging med surg* 2021; **11**(4): 1184-95.

258. Xu R, Kido S, Suga K, Hirano Y, Tachibana R, Muramatsu K, et al. Texture analysis on (18)F-FDG PET/CT images to differentiate malignant and benign bone and soft-tissue lesions. *Ann Nucl Med* 2014; **28**(9): 926-35.

259. Xu W, Hao D, Hou F, Zhang D, Wang H. Soft Tissue Sarcoma: Preoperative MRI-Based Radiomics and Machine Learning May Be Accurate Predictors of Histopathologic Grade. *AJR Am J Roentgenol* 2020; **215**(4): 963-9.

260. Xu Z, Niu K, Tang S, Song T, Rong Y, Guo W, He Z. Bone tumor necrosis rate detection in few-shot X-rays based on deep learning. *Comput Med Imaging Graph* 2022; **102**.

261. Yamazawa E, Takahashi S, Shin M, Tanaka S, Takahashi W, Nakamoto T, et al. MRI-Based Radiomics Differentiates Skull Base Chordoma and Chondrosarcoma: A Preliminary Study. *Cancers (Basel)* 2022; **14**(13).

262. Yan J, Zhao X, Han S, Wang T, Miao F. Evaluation of Clinical Plus Imaging Features and Multidetector Computed Tomography Texture Analysis in Preoperative Risk Grade Prediction of Small Bowel Gastrointestinal Stromal Tumors. *J Comput Assist Tomogr* 2018; **42**(5): 714-20.

263. Yan M, Liu Y, You H, Zhao Y, Jin J, Wang J. Differentiation of Small Gastrointestinal Stromal Tumor and Gastric Leiomyoma with Contrast-Enhanced CT. *J healthc eng* 2023; **2023**: 6423617.

264. Yan R, Hao D, Li J, Liu J, Hou F, Chen H, et al. Magnetic Resonance Imaging-Based Radiomics Nomogram for Prediction of the Histopathological Grade of Soft Tissue Sarcomas: A Two-Center Study. *J Magn Reson Imaging* 2021; **53**(6): 1683-96.

265. Yang F, Feng Y, Sun P, Traverso A, Dekker A, Zhang B, et al. Preoperative prediction of high-grade osteosarcoma response to neoadjuvant therapy based on a plain CT radiomics model: A dual-center study. *J Bone Oncol* 2024; **47**: 100614.

266. Yang J, Chen Z, Liu W, Wang X, Ma S, Jin F, Wang X. Development of a Malignancy Potential Binary Prediction Model Based on Deep Learning for the Mitotic Count of Local Primary Gastrointestinal Stromal Tumors. *Korean J Radiol* 2021; **22**(3): 344-53.

267. Yang L, Du D, Zheng T, Liu L, Wang Z, Du J, et al. Deep learning and radiomics to predict the mitotic index of gastrointestinal stromal tumors based on multiparametric MRI. *Front oncol* 2022; **12**: 948557.

268. Yang L, Ma CF, Li Y, Zhang CR, Ren JL, Shi GF. Application of radiomics in predicting the preoperative risk stratification of gastric stromal tumors. *Diagn Interv Radiol* 2022; **28**(6): 532-9.

269. Yang L, Zhang D, Zheng T, Liu D, Fang Y. Predicting the progression-free survival of gastrointestinal stromal tumors after imatinib therapy through multi-sequence magnetic resonance imaging. *Abdom Radiol* 2024; **49**(3): 801-13.

270. Yang L, Zheng T, Dong Y, Wang Z, Liu D, Du J, et al. MRI Texture-Based Models for Predicting Mitotic Index and Risk Classification of Gastrointestinal Stromal Tumors. *J Magn Reson Imaging* 2021; **53**(4): 1054-65.

271. Yang P, Wu J, Liu M, Zheng Y, Zhao X, Mao Y. Preoperative CT-based radiomics and deep learning model for predicting risk stratification of gastric gastrointestinal stromal tumors. *Med Phys* 2024; **51**(10): 7257-68.

272. Yang X, Wang H, Dong Q, Xu Y, Liu H, Ma X, et al. An artificial intelligence system for distinguishing between gastrointestinal stromal tumors and leiomyomas using endoscopic ultrasonography. *Endoscopy* 2022; **54**(3): 251-61.

273. Yang Y, Ma X, Wang Y, Ding X. Prognosis prediction of extremity and trunk wall soft-tissue sarcomas treated with surgical resection with radiomic analysis based on random survival forest. *Updates Surg* 2022; **74**(1): 355-65.

274. Yang Y, Zhou Y, Zhou C, Ma X. Novel computer aided diagnostic models on multimodality medical images to differentiate well differentiated liposarcomas from lipomas approached by deep learning methods. *Orphanet J Rare Dis* 2022; **17**(1): 158.

275. Yang Y, Zhou Y, Zhou C, Zhang X, Ma X. MRI-Based Computer-Aided Diagnostic Model to Predict Tumor Grading and Clinical Outcomes in Patients With Soft Tissue Sarcoma. *J Magn Reson Imaging* 2022; **56**(6): 1733-45.

276. Ye Q, Yang H, Lin B, Wang M, Song L, Xie Z, et al. Automatic detection, segmentation, and classification of primary bone tumors and bone infections using an ensemble multi-task deep learning framework on multi-parametric MRIs: a multi-center study. *Eur Radiol* 2024; **34**(7): 4287-99.

277. Yildiz Potter I, Yeritsyan D, Mahar S, Wu J, Nazarian A, Vaziri A, Vaziri A. Automated Bone Tumor Segmentation and Classification as Benign or Malignant Using Computed Tomographic Imaging. *J Digit Imaging* 2023; **36**(3): 869-78.

278. Yin P, Mao N, Chen H, Sun C, Wang S, Liu X, Hong N. Machine and Deep Learning Based Radiomics Models for Preoperative Prediction of Benign and Malignant Sacral Tumors. *Front Oncol* 2020; **10**.

279. Yin P, Mao N, Liu X, Sun C, Wang S, Chen L, Hong N. Can clinical radiomics nomogram based on 3D multiparametric MRI features and clinical characteristics estimate early recurrence of pelvic chondrosarcoma? *J Magn Reson Imaging* 2020; **51**(2): 435-45.

280. Yin P, Mao N, Wang S, Sun C, Hong N. Clinical-radiomics nomograms for pre-operative differentiation of sacral chordoma and sacral giant cell tumor based on 3D computed tomography and multiparametric magnetic resonance imaging. *Br J Radiol* 2019; **92**(1101): 20190155.

281. Yin P, Mao N, Zhao C, Wu J, Chen L, Hong N. A Triple-Classification Radiomics Model for the Differentiation of Primary Chordoma, Giant Cell Tumor, and Metastatic Tumor of Sacrum Based on T2-Weighted and Contrast-Enhanced T1-Weighted MRI. *J Magn Reson Imaging* 2019; **49**(3): 752-9.

282. Yin P, Mao N, Zhao C, Wu J, Sun C, Chen L, Hong N. Comparison of radiomics machine-learning classifiers and feature selection for differentiation of sacral chordoma and sacral giant cell tumour based on 3D computed tomography features. *Eur Radiol* 2019; **29**(4): 1841-7.

283. Yin P, Sun C, Wang S, Chen L, Hong N. Clinical-Deep Neural Network and Clinical-Radiomics Nomograms for Predicting the Intraoperative Massive Blood Loss of Pelvic and Sacral Tumors. *Front Oncol* 2021; **11**.

284. Yin P, Wang W, Wang S, Liu T, Sun C, Liu X, et al. The potential for different computed tomography-based machine learning networks to automatically segment and differentiate pelvic and sacral osteosarcoma from Ewing's sarcoma. *Quant imaging med surg* 2023; **13**(5): 3174-84.

285. Yin P, Zhong J, Liu Y, Liu T, Sun C, Liu X, et al. Clinical-radiomics models based on plain X-rays for prediction of lung metastasis in patients with osteosarcoma. *BMC med imaging* 2023; **23**(1): 40.

286. Yin XN, Wang ZH, Zou L, Yang CW, Shen CY, Liu BK, et al. Computed tomography radiogenomics: A potential tool for prediction of molecular subtypes in gastric stromal tumor. *World J Gastrointest Oncol* 2024; **16**(4): 1296-308.

287. Yisheng X, Yueqin L, Ming Z. Diagnostic significance of multisequence MRI radiomics models in distinguishing benign and malignant spinal fractures. *J Radiat Res Appl Sci* 2024; **17**(3).

288. Yoon H, Choi WH, Joo MW, Ha S, Chung YA. SPECT/CT Radiomics for Differentiating between Enchondroma and Grade I Chondrosarcoma. *Tomography* 2023; **9**(5): 1868-75.

289. Yu Y, Guo H, Zhang M, Hou F, Yang S, Huang C, et al. Multi-institutional validation of a radiomics signature for identification of postoperative progression of soft tissue sarcoma. *Cancer Imaging* 2024; **24**(1): 59.

290. Yue Z, Wang X, Wang Y, Wang H, Jiang W. Clinical-Radiomics Nomogram from T1W, T1CE, and T2FS MRI for Improving Diagnosis of Soft-Tissue Sarcoma. *Mol Imaging Biol* 2022; **24**(6): 995-1006.

291. Yue Z, Wang X, Yu T, Shang S, Liu G, Jing W, et al. Multi-parametric MRI-based radiomics for the diagnosis of malignant soft-tissue tumor. *Magn Reson Imaging* 2022; **91**: 91-9.

292. Yuguang Y, Chen Y, Zhu D, Huang Y, Huang Y, Li X, Xiahou J. GHA-DenseNet prediction and diagnosis of malignancy in femoral bone tumors using magnetic resonance imaging. *J Bone Oncol* 2024; **44**: 100520.

293. Zhai Y, Bai J, Xue Y, Li M, Mao W, Zhang X, Zhang Y. Development and validation of a preoperative MRI-based radiomics nomogram to predict progression-free survival in patients with clival chordomas. *Front oncol* 2022; **12**: 996262.

294. Zhang C, Wang C, Mao G, Cheng G, Ji H, He L, et al. Radiomics analysis of contrast-enhanced computerized tomography for differentiation of gastric schwannomas from gastric gastrointestinal stromal tumors. *J Cancer Res Clin Oncol* 2024; **150**(2): 87.

295. Zhang C, Wang J, Yang Y, Dai B, Xu Z, Zhu F, Yu H. Machine learning for predicting the risk stratification of 1-5 cm gastric gastrointestinal stromal tumors based on CT. *BMC med imaging* 2023; **23**(1): 90.

296. Zhang L, Gao Q, Dou Y, Cheng T, Xia Y, Li H, Gao S. Evaluation of the neoadjuvant chemotherapy response in osteosarcoma using the MRI DWI-based machine learning radiomics nomogram. *Front oncol* 2024; **14**: 1345576.

297. Zhang L, Ge Y, Gao Q, Zhao F, Cheng T, Li H, Xia Y. Machine Learning-Based Radiomics Nomogram With Dynamic Contrast-Enhanced MRI of the Osteosarcoma for Evaluation of Efficacy of Neoadjuvant Chemotherapy. *Front oncol* 2021; **11**: 758921.

298. Zhang L, Kang L, Li G, Zhang X, Ren J, Shi Z, et al. Computed tomography-based radiomics model for discriminating the risk stratification of gastrointestinal stromal tumors. *Radiol Med (Torino)* 2020; **125**(5): 465-73.

299. Zhang L, Ren Z. Comparison of CT and MRI images for the prediction of soft-tissue sarcoma grading and lung metastasis via a convolutional neural networks model. *Clin Radiol* 2020; **75**(1): 64-9.

300. Zhang L, Yang Y, Wang T, Chen X, Tang M, Deng J, et al. Intratumoral and peritumoral MRI-based radiomics prediction of histopathological grade in soft tissue sarcomas: a two-center study. *Cancer Imaging* 2023; **23**(1): 103.

301. Zhang M, Tong E, Hamrick F, Lee EH, Tam LT, Pendleton C, et al. Machine-Learning Approach to Differentiation of Benign and Malignant Peripheral Nerve Sheath Tumors: A Multicenter Study. *Neurosurgery* 2021; **89**(3): 509-17.

302. Zhang M, Tong E, Wong S, Hamrick F, Mohammadzadeh M, Rao V, et al. Machine learning approach to differentiation of peripheral schwannomas and neurofibromas: A multi-center study. *Neuro-oncol* 2022; **24**(4): 601-9.

303. Zhang QW, Gao YJ, Zhang RY, Zhou XX, Chen SL, Zhang Y, et al. Personalized CT-based radiomics nomogram preoperative predicting Ki-67 expression in gastrointestinal stromal tumors: a multicenter development and validation cohort. *Clin Transl Med* 2020; **9**(1): 12.

304. Zhang QW, Zhang RY, Yan ZB, Zhao YX, Wang XY, Jin JZ, et al. Personalized radiomics signature to screen for KIT-11 mutation genotypes among patients with gastrointestinal stromal tumors: a retrospective multicenter study. *J transl med* 2023; **21**(1): 726.

305. Zhang QW, Zhou XX, Zhang RY, Chen SL, Liu Q, Wang J, et al. Comparison of malignancy-prediction efficiency between contrast and non-contract CT-based radiomics features in gastrointestinal stromal tumors: A multicenter study. *Clin Transl Med* 2020; **10**(3): e291.

306. Zhang XD, Zhang L, Gong TT, Wang ZR, Guo KL, Li J, et al. A combined radiomic model distinguishing GISTs from leiomyomas and schwannomas in the stomach based on endoscopic ultrasonography images. *J appl clin med phys* 2023; **24**(7): e14023.

307. Zhang Y, Yue X, Zhang P, Zhang Y, Wu L, Diao N, et al. Clinical-radiomics-based treatment decision support for KIT Exon 11 deletion in gastrointestinal stromal tumors: a multi-institutional retrospective study. *Front oncol* 2023; **13**: 1193010.

308. Zhang Y, Zhao H, Liu Y, Zeng M, Zhang J, Hao D. Diagnostic Performance of Dynamic Contrast-Enhanced MRI and 18F-FDG PET/CT for Evaluation of Soft Tissue Tumors and Correlation with Pathology Parameters. *Academic Radiology* 2022.

309. Zhang Y, Zhu Y, Shi X, Tao J, Cui J, Dai Y, et al. Soft Tissue Sarcomas: Preoperative Predictive Histopathological Grading Based on Radiomics of MRI. *Acad Radiol* 2019; **26**(9): 1262-8.

310. Zhao K, Zhang M, Xie Z, Yan X, Wu S, Liao P, et al. Deep Learning Assisted Diagnosis of Musculoskeletal Tumors Based on Contrast-Enhanced Magnetic Resonance Imaging. *J Magn Reson Imaging* 2022; **56**(1): 99-107.

311. Zhao S, Su Y, Duan J, Qiu Q, Ge X, Wang A, Yin Y. Radiomics signature extracted from diffusion-weighted magnetic resonance imaging predicts outcomes in osteosarcoma. *J Bone Oncol* 2019; **19**: 100263.

312. Zhao Y, Feng M, Wang M, Zhang L, Li M, Huang C. CT Radiomics for the Preoperative Prediction of Ki67 Index in Gastrointestinal Stromal Tumors: A Multi-Center Study. *Front oncol* 2021; **11**: 689136.

313. Zheng F, Yin P, Liang K, Wang Y, Hao W, Hao Q, Hong N. Fusion Radiomics-Based Prediction of Response to Neoadjuvant Chemotherapy for Osteosarcoma. *Acad Radiol* 2024; **31**(6): 2444-55.

314. Zheng F, Yin P, Liang KW, Liu T, Wang YJ, Hao WH, et al. Comparison of Different Fusion Radiomics for Predicting Benign and Malignant Sacral Tumors: A Pilot Study. *Journal of Imaging Informatics in Medicine* 2024.

315. Zheng J, Liao Q, Chen X, Hong M, Mazzocca A, Urbini M, et al. Development and validation of a computed tomography-based radiomics signature to predict "highest-risk" from patients with high-risk gastrointestinal stromal tumor. *J gastrointest oncol* 2024; **15**(1): 125-33.

316. Zheng J, Xia Y, Xu A, Weng X, Wang X, Jiang H, et al. Combined model based on enhanced CT texture features in liver metastasis prediction of high-risk gastrointestinal stromal tumors. *Abdom Radiol* 2022; **47**(1): 85-93.

317. Zheng Y, Chen L, Liu M, Wu J, Yu R, Lv F. Prediction of Clinical Outcome for High-Intensity Focused Ultrasound Ablation of Uterine Leiomyomas Using Multiparametric MRI Radiomics-Based Machine Leaning Model. *Front oncol* 2021; **11**: 618604.

318. Zheng Y, Chen L, Liu M, Wu J, Yu R, Lv F. Nonenhanced MRI-based radiomics model for preoperative prediction of nonperfused volume ratio for high-intensity focused ultrasound ablation of uterine leiomyomas. *Int J Hyperthermia* 2021; **38**(1): 1349-58.

319. Zhong J, Zhang C, Hu Y, Zhang J, Liu Y, Si L, et al. Automated prediction of the neoadjuvant chemotherapy response in osteosarcoma with deep learning and an MRI-based radiomics nomogram. *Eur Radiol* 2022; **32**(9): 6196-206.

320. Zhou Y, Zhang J, Chen J, Yang C, Gong C, Li C, Li F. Prediction using T2-weighted magnetic resonance imaging-based radiomics of residual uterine myoma regrowth after high-intensity focused ultrasound ablation. *Ultrasound Obstet Gynecol* 2022; **60**(5): 681-92.

321. Zhou Z, Xie P, Dai Z, Wu J. Self-supervised tumor segmentation and prognosis prediction in osteosarcoma using multiparametric MRI and clinical characteristics. *Comput Methods Programs Biomed* 2024; **244**: 107974.

322. Zhu MP, Ding QL, Xu JX, Jiang CY, Wang J, Wang C, Yu RS. Building contrast-enhanced CT-based models for preoperatively predicting malignant potential and Ki67 expression of small intestine gastrointestinal stromal tumors (GISTs). *Abdom Radiol* 2022; **47**(9): 3161-73.

323. Zhuo M, Chen X, Guo J, Qian Q, Xue E, Chen Z. Deep Learning-Based Segmentation and Risk Stratification for Gastrointestinal Stromal Tumors in Transabdominal Ultrasound Imaging. *J Ultrasound Med* 2024; **43**(9): 1661-72.

324. Zhuo M, Guo J, Tang Y, Tang X, Qian Q, Chen Z. Ultrasound radiomics model-based nomogram for predicting the risk Stratification of gastrointestinal stromal tumors. *Front oncol* 2022; **12**: 905036.

325. Zhuo M, Tang Y, Guo J, Qian Q, Xue E, Chen Z. Predicting the risk stratification of gastrointestinal stromal tumors using machine learning-based ultrasound radiomics. *J Med Ultrason (2001)* 2024; **51**(1): 71-82.
