# Supplementary material for: AI in radiological imaging of soft-tissue and bone tumours: a systematic review evaluating against CLAIM and FUTURE-AI guidelines
Source: eBioMedicine. 2025 Mar 20;114:105642. doi: 10.1016/j.ebiom.2025.105642 (PMC11976239; doi:10.1016/j.ebiom.2025.105642)
Supplement: Supplementary Figures [file mmc4.docx]

**Supplementary Figures**

**Figure S1:** Inter-reader variability sub-group analysis (n=30) for criteria of the Checklist for Artificial Intelligence in Medical Imaging (CLAIM). Agreement before (green) and after (orange) consensus discussion is reported between raters.


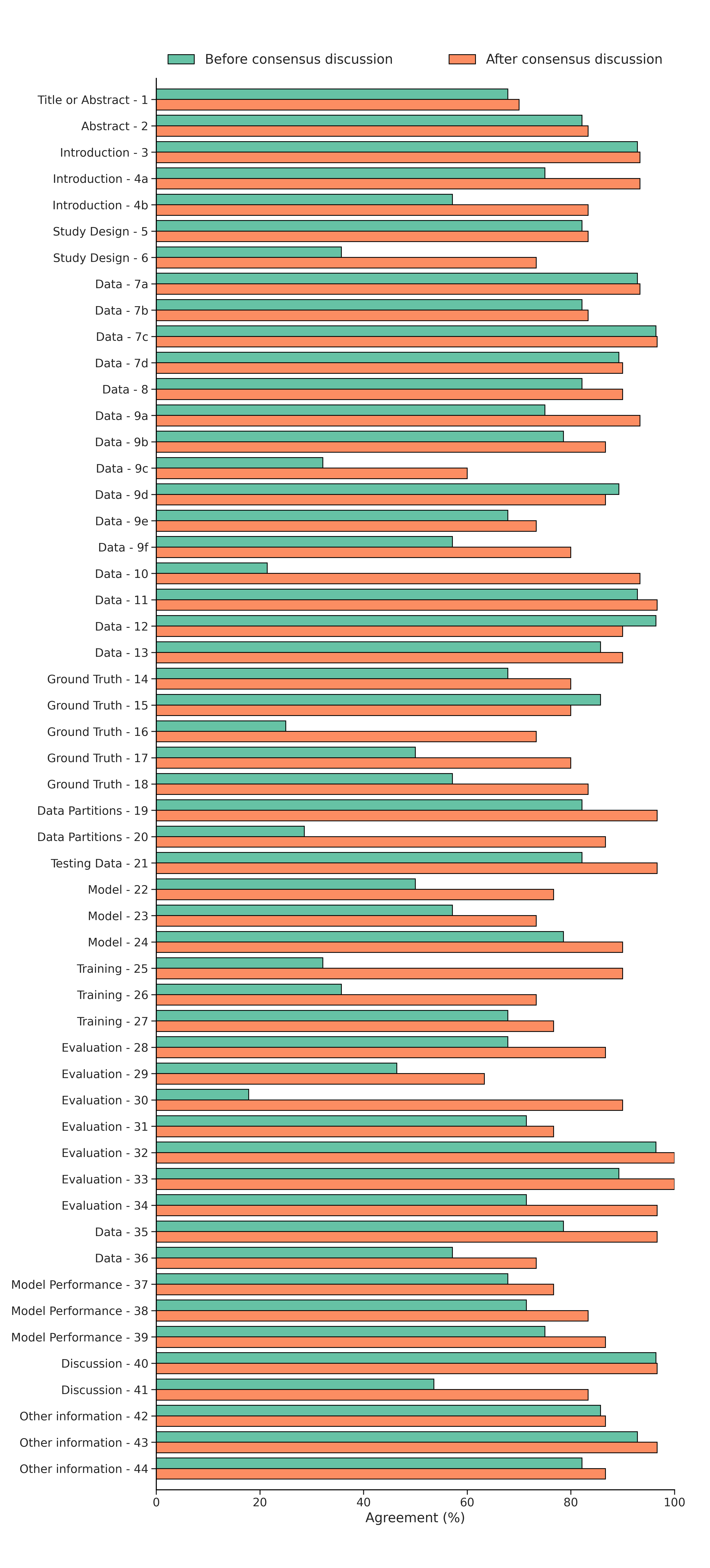


**Figure S2:** Inter-reader variability sub-group analysis (n=30) for criteria of the FUTURE-AI international consensus guideline for trustworthy and deployable AI. Agreement before (green) and after (orange) consensus discussion is reported between raters.


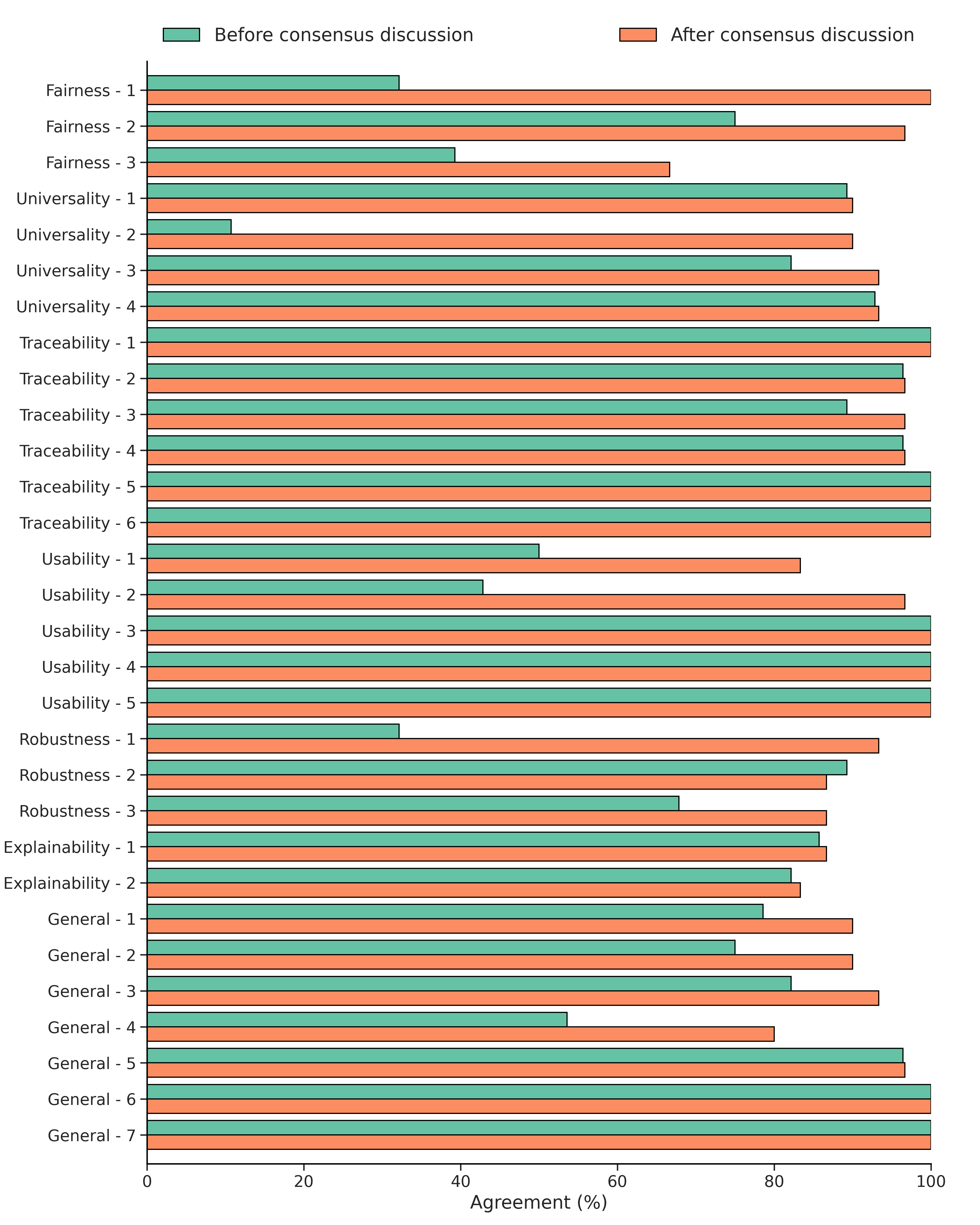


**Figure S3:** Trend of scores on the Checklist for Artificial Intelligence in Medical Imaging (CLAIM) for each year across included studies (n=325). Red dots represent the mean score for each year, while each blue dot corresponds to a single study, with their positions slightly adjusted to avoid overlap. The regression line is calculated with the starting point (x = 0) set to 2008.


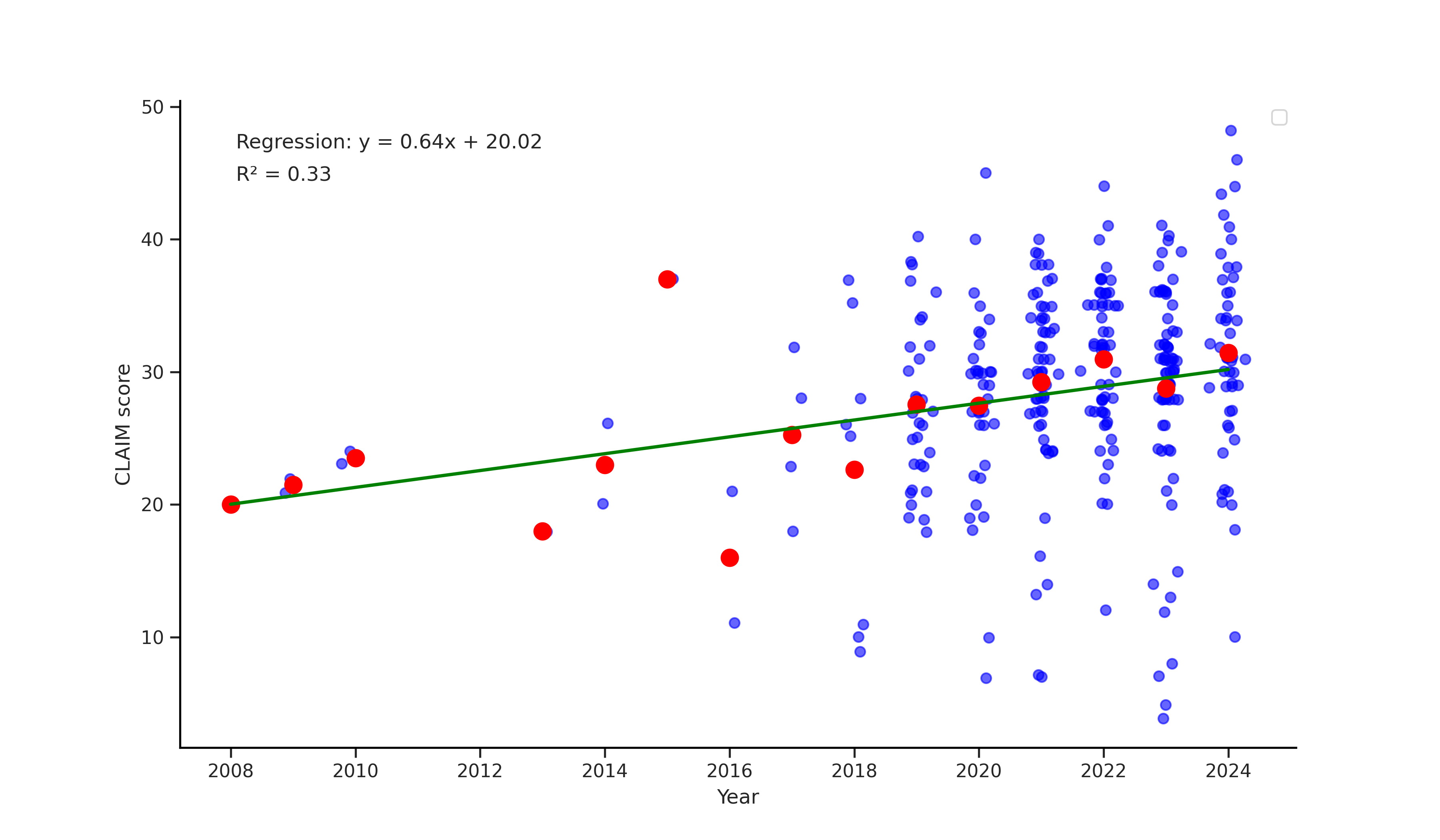


**Figure S4:** Trend of scores on the FUTURE-AI international consensus guideline for trustworthy and deployable AI for each year across included studies (n=325). Red dots represent the mean score for each year, while each blue dot corresponds to a single study, with their positions slightly adjusted to avoid overlap. The regression line is calculated with the starting point (x = 0) set to 2008.


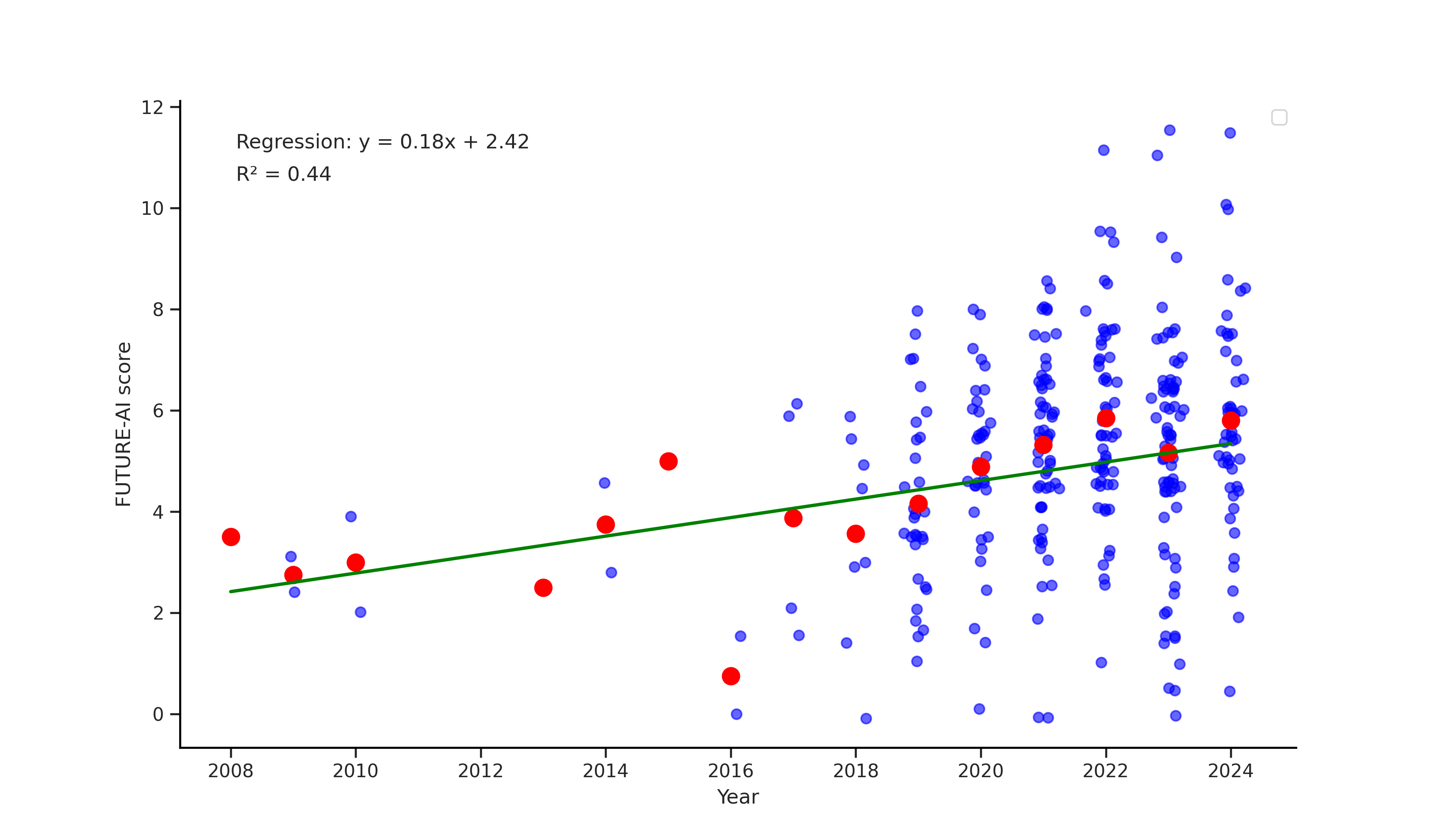


**Figure S5:** Scores on the Checklist for Artificial Intelligence in Medical Imaging (CLAIM) for different AI methods, disease types and predicted outcomes across included studies (n=325).


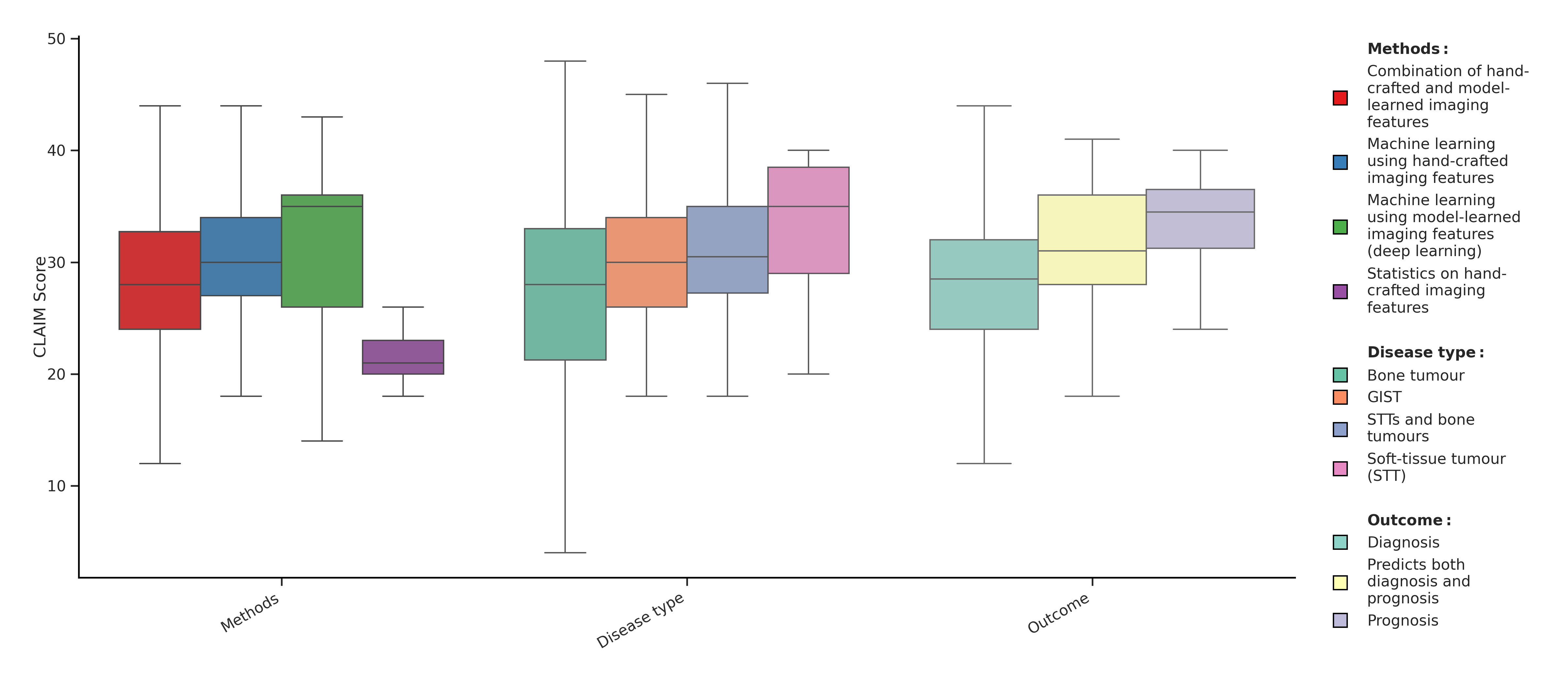


**Figure S6:** Scores on the FUTURE-AI international consensus guideline for trustworthy and deployable AI for different AI methods, disease types and predicted outcomes across included studies (n=325).


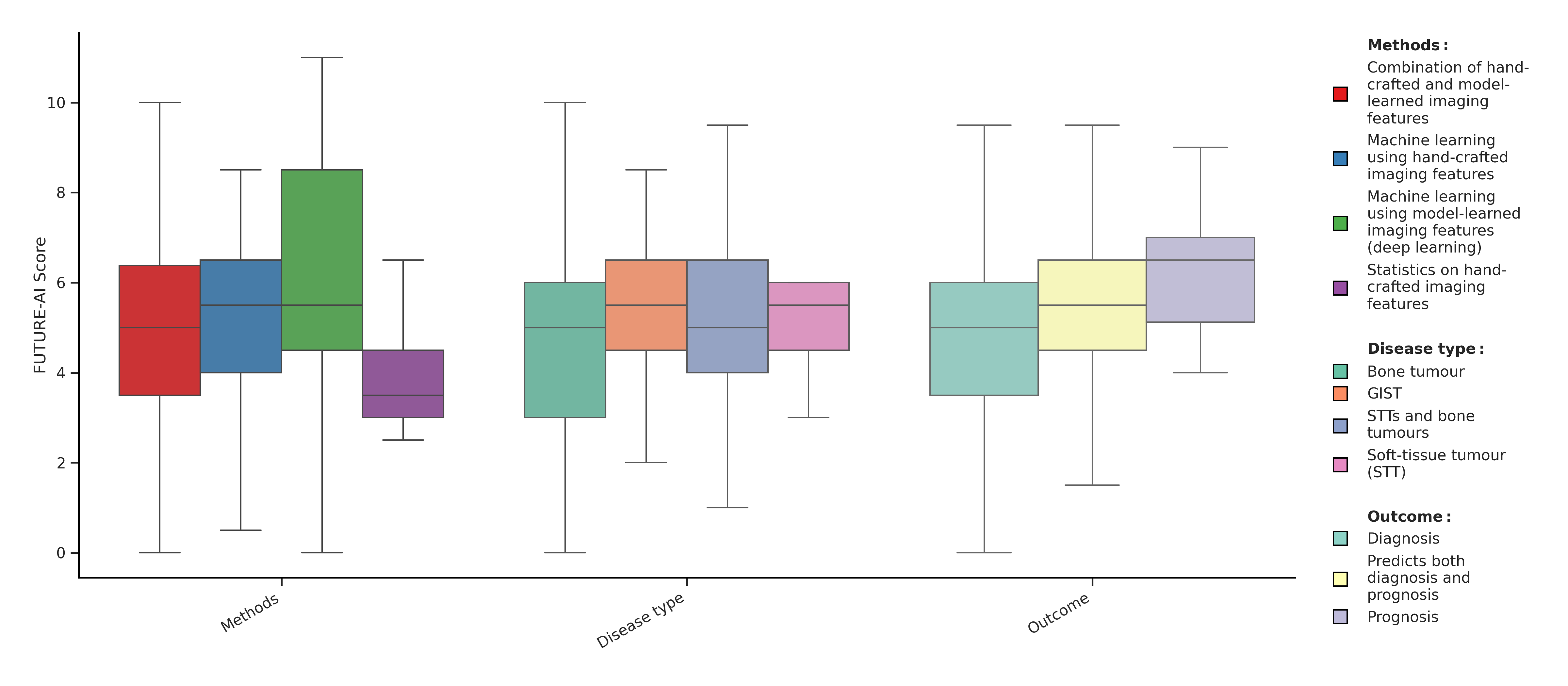


**Figure S7:** Reported and unreported criteria for each study (n=325) from the Checklist for Artificial Intelligence in Medical Imaging (CLAIM). An interactive version of this plot can be found at: <https://douwe-spaanderman.github.io/AI-STTandBoneTumour-Review/>

**Figure S8:** Scores of each study (n=325) for each criterion from the FUTURE-AI international consensus guideline for trustworthy and deployable AI. An interactive version of this plot can be found at: <https://douwe-spaanderman.github.io/AI-STTandBoneTumour-Review/>
