# Supplementary material for: AI in radiological imaging of soft-tissue and bone tumours: a systematic review evaluating against CLAIM and FUTURE-AI guidelines
Source: eBioMedicine. 2025 Mar 20;114:105642. doi: 10.1016/j.ebiom.2025.105642 (PMC11976239; doi:10.1016/j.ebiom.2025.105642)
Supplement: Supplementary Table S1 [file mmc5.docx]

**Supplementary Tables**

**Table S1:** Score analysis for different predicted outcomes, disease types, and AI methods of the top 20 studies in terms of highest combined CLAIM and FUTURE-AI score.

| Categories | | N | Mean CLAIM score | Mean FUTURE-AI score | AUC range | Accuracy range | Sensitivity range | Specificity range |
| --- | --- | --- | --- | --- | --- | --- | --- | --- |
| Outcome type | Diagnosis | 12 | 41·2 | 8·7 | 0·78-0·95 | 0·69-0·92 | 0·57-1·00 | 0·63-0·93 |
|  | Prognosis | 7 | 39·0 | 7·9 | 0·64-0·93 | 0·77-0·90 | 0·20-0·79 | 0·68-0·97 |
|  | Both^a^ | 1 | 40·0 | 9·0 | 0·82-0·86 | 0·67-0·84 | 0·67-0·84 | 0·74-1·00 |
| Disease type^b^ | Bone tumour | 8 | 41·4 | 8·1 | 0·78-0·94 | 0·69-0·92 | 0·57-0·90 | 0·67-0·92 |
|  | Soft-tissue tumour | 9 | 38·9 | 8·6 | 0·75-0·93 | 0·67-0·90 | 0·20-1·00 | 0·63-1·00 |
|  | GIST | 3 | 42·0 | 8·7 | 0·64-0·95 | 0·75-0·92 | 0·24-0·90 | 0·75-0·93 |
| Method type | Hand-crafted features | 11 | 39·5 | 7·6 | 0·75-0·94 | 0·75-0·92 | 0·20-0·92 | 0·67-0·95 |
|  | Model-learned features | 6 | 42·8 | 9·3 | 0·64-0·95 | 0·67-0·92 | 0·24-0·90 | 0·74-1·00 |
|  | Combined hand-crafted and model-learned features | 3 | 38·3 | 9·5 | 0·80-0·86 | 0·80-0·86 | 0·47-1·00 | 0·63-0·97 |

The ranges presented in the table are derived from the minimum and maximum values reported for each metric across the selected studies.
^a^ Performance metrics were reported from three external validation sites, contributing to the observed ranges.
^b^ No papers investigating both Soft-tissue tumour (STT) and Bone tumours were in the top 20 scoring papers.
